# Ancient genomics reveals tripartite origins of Japanese populations

**DOI:** 10.1126/sciadv.abh2419

**Published:** 2021-09-17

**Authors:** Niall P. Cooke, Valeria Mattiangeli, Lara M. Cassidy, Kenji Okazaki, Caroline A. Stokes, Shin Onbe, Satoshi Hatakeyama, Kenichi Machida, Kenji Kasai, Naoto Tomioka, Akihiko Matsumoto, Masafumi Ito, Yoshitaka Kojima, Daniel G. Bradley, Takashi Gakuhari, Shigeki Nakagome

**Affiliations:** 1 School of Medicine, Trinity College Dublin, Dublin, Ireland.; 2 Smurfit Institute of Genetics, Trinity College Dublin, Dublin, Ireland.; 3 Department of Anatomy, Faculty of Medicine, Tottori University, Japan.; 4 Kumakogen Board of Education, Kumakogen, Japan.; 5 Tobinodai Historic Site Park Museum, Funabashi, Japan.; 6 Toyama Prefectural Research Office for Archaeological Heritage, Toyama, Japan.; 7 Toyama Prefectural Center for Archaeological Operations, Toyama, Japan.; 8 Okayama University of Science, Okayama, Japan.; 9 Ainan Board of Education, Ainan, Japan.; 10 Foundation of Ishikawa Archaeological Artifacts Center, Kanazawa, Japan.; 11 Center for the Study of Ancient Civilizations and Cultural Resources, College of Human and Social Sciences, Kanazawa University, Kanazawa, Japan.

## Abstract

Prehistoric Japan underwent rapid transformations in the past 3000 years, first from foraging to wet rice farming and then to state formation. A long-standing hypothesis posits that mainland Japanese populations derive dual ancestry from indigenous Jomon hunter-gatherer-fishers and succeeding Yayoi farmers. However, the genomic impact of agricultural migration and subsequent sociocultural changes remains unclear. We report 12 ancient Japanese genomes from pre- and postfarming periods. Our analysis finds that the Jomon maintained a small effective population size of ~1000 over several millennia, with a deep divergence from continental populations dated to 20,000 to 15,000 years ago, a period that saw the insularization of Japan through rising sea levels. Rice cultivation was introduced by people with Northeast Asian ancestry. Unexpectedly, we identify a later influx of East Asian ancestry during the imperial Kofun period. These three ancestral components continue to characterize present-day populations, supporting a tripartite model of Japanese genomic origins.

## Teaser

Ancient genomes uncover multiple migratory pulses to Japan with the spread of wet rice farming and the rise of the state.

## INTRODUCTION

The Japanese archipelago has been occupied by humans for at least 38,000 years. However, its most radical cultural transformations have only occurred within the past 3000 years, during which time its inhabitants quickly transitioned from foraging to widespread rice farming to a technologically advanced imperial state (*1*, *2*). These rapid changes, coupled with geographical isolation from continental Eurasia, make Japan a unique microcosm in which to study the migratory patterns that accompanied agricultural spread and economic intensification in Asia. Before the arrival of farming cultures, the archipelago was occupied by diverse hunter-gatherer-fisher groups belonging to the Jomon culture, characterized by their use of pottery. The Jomon period began during the Oldest Dryas that followed the Last Glacial Maximum (LGM) (*3*), with the earliest pottery shards dating to ~16,500 years ago (ka ago), making these populations some of the oldest users of ceramics in the world (*2*). Jomon subsistence strategies varied and population densities fluctuated through space and time (*4*), with trends toward sedentism. This culture continued until the beginning of the Yayoi period (~3 ka ago), when the arrival of paddy field rice cultivation led to an agricultural revolution in the archipelago. This was followed by the Kofun period, starting ~1.7 ka ago, which saw the emergence of political centralization and the imperial reign that came to define the region (*1*).

An enduring hypothesis on the origin of modern Japanese populations proposes a dual-structure model (*5*), in which Japanese populations are the admixed descendants of the indigenous Jomon and later arrivals from the East Eurasian continent during the Yayoi period. This hypothesis was originally proposed on the basis of morphological data but has been widely tested and evaluated across disciplines [see a recent review in (*6*)]. Genetic studies have identified population stratifications within present-day Japanese populations, supporting at least two waves of migrations to the Japanese archipelago (*7*–*10*). Previous ancient DNA studies have also illustrated the genetic affinity of Jomon and Yayoi individuals to Japanese populations today (*11*–*15*). Still, the demographic origins and impact of the agricultural transition and later state formation phase are largely unknown. From a historical linguistic standpoint, the arrival of proto-Japonic language is theorized to map to the development of Yayoi culture and the spread of wet rice cultivation (*6*). However, archaeological contexts and their continental affiliations are distinct between the Yayoi and Kofun periods (*1*); whether the spread of knowledge and technology was accompanied by major genetic exchange remains elusive.

**Fig. 1.**
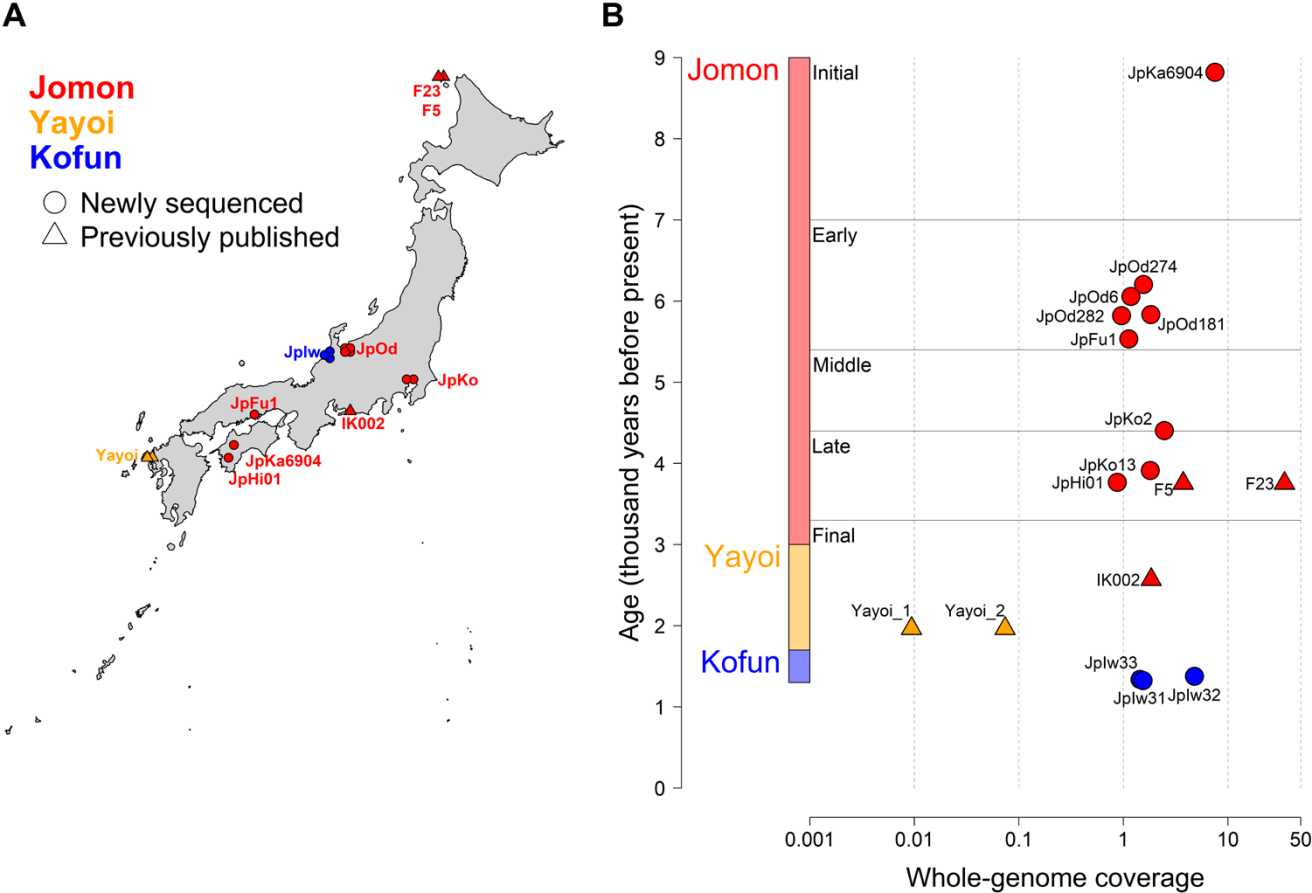
Sampling locations, dates, and genome coverage of ancient Japanese individuals. (**A**) Archaeological sites are marked with circles for individual genomes newly sequenced in this study and triangles if previously reported (see Table 1 and table S1). The colors represent three different periods of Japanese pre- and protohistory: Jomon, Yayoi, and Kofun. (**B**) Each individual is plotted with whole-genome coverage on the *x* axis and median age (years before present) on the *y* axis. The nine Jomon individuals are split into five different subperiods on the basis of their ages (see note S1): Initial (JpKa6904), Early (JpOd274, JpOd6, JpOd282, JpOd181, and JpFu1), Middle (JpKo2), Late (JpKo13, JpHi01, F23, and F5), and Final (IK002).

Here, we report 12 newly sequenced ancient Japanese genomes spanning 8000 years of the archipelago’s pre- and protohistory (Fig. 1 and Table 1). To our knowledge, this is the largest set of time-stamped genomes from the archipelago, including the oldest Jomon individual and the first genomic data from the imperial Kofun period. We also include five published prehistoric Japanese genomes in our analysis: three Jomon individuals (F5 and F23 from the Late Jomon period and IK002 from the Final Jomon period) (*12*–*14*), as well as two 2000-year-old individuals associated with the Yayoi culture from the northwestern part of Kyushu Island, where skeletal remains exhibit Jomon-like characters rather than immigrant types but other archaeological materials clearly support their association with the Yayoi culture (*15*, *16*). Despite this morphological assessment (*16*), these two Yayoi individuals show an increased genetic affinity to present-day Japanese populations compared with the Jomon, implying that admixture with continental groups was already advanced by the Late Yayoi period (*15*). Integrating these Japanese genomes with a larger ancient genomic dataset spanning the Central and Eastern Steppe (*17*, *18*), Siberia (*19*), Southeast Asia (*12*), and East Asia (*15*, *20*, *21*), our study aims to better characterize the preagricultural populations of the Jomon period, as well as the subsequent migrations and admixtures that have shaped the genetic profile of the archipelago today.

**Table 1.** Summary of ancient Japanese data.

**Associated** **culture**	**Sample ID**	**Date range** **and median** **(cal B.P.)**	**Coverage**	**mtDNA** **contamination rate** **(%)**	**Molecular sex**	**mtDNA** **haplogroup**	**Y chromosome** **haplogroup**	**Ref.**
Newly sequenced in this study								
Jomon	JpKa6904	8646–8991; 8819	7.51	1.46	XX	N9b3	–	–
	JpOd274	6119–6289; 6204	1.56	1.13	XY	M7a	D1b1d1	–
	JpOd6	5934–6179; 6057	1.18	1.55	XX	N9b3	–	–
	JpOd181	5751–5917; 5834	1.83	0.91	XY	N9b1	D1b1d1	–
	JpOd282	5737–5902; 5820	0.96	1.38	XY	M7a1	D1b1d1	–
	JpFu1	5478–5590; 5534	1.13	2.15	XX	M7a1	–	–
	JpKo2	4294–4514; 4404	2.47	1.44	XX	N9b	–	–
	JpKo13	3847–3978; 3913	1.81	1.50	XX	N9b1	–	–
	JpHi01	3685–3850; 3768	0.88	1.45	XX	M7a1a	–	–
Kofun	JpIw32	1347–1409; 1378	4.80	0.41	XY	B5a2a1b	O3a2c	–
	JpIw31	1303–1377; 1340	1.44	0.63	XX	D5c1a	–	–
	JpIw33	1295–1355; 1325	1.54	0.75	XX	M7b1a1a1	–	–
Previously published								
Jomon	F23	3550–3960; 3755	34.82	1.20	XX	N9b1	-	(*14*)
	F5	–	3.74	2.45	XY	N9b1	D1b2b	(*14*)
	IK002	2418–2720; 2569	1.85	0.50	XX	N9b1	–	(*12*)
Yayoi	Yayoi_1	–	0.01	2.92	XX	M7a1a4	–	(*15*)
	Yayoi_2	1931–2001; 1966	0.07	2.33	XY	D4a1	O	(*15*)

## RESULTS

### A time series of ancient genomes from pre- and protohistoric Japan

Our initial screening focused on 14 ancient skeletal remains excavated from six archaeological sites across the archipelago (see note S1). High levels of endogenous human DNA were preserved in 12 of these samples (Table 1), which were then further shotgun-sequenced to higher coverage, ranging from 0.88× to 7.51× (Fig. 1 and table S1). Nine of the 12 samples are associated with the Jomon culture, representing the west and central parts of the archipelago and four different stages of the Jomon period (Initial, Early, Middle, and Late Jomon) (Fig. 1). The remaining three samples date to ~1.3 ka ago, falling within the Kofun period. We confirm that all newly sequenced genomes show postmortem damage patterns (fig. S1) and a low level of modern human contamination (<2.15%) (Table 1 and table S2). Our kinship analysis confirms that all pairs of individuals are unrelated (fig. S2). Mitochondrial haplogroups for all Jomon individuals belong to the N9b or M7a clades, which are strongly associated with this population (*11*–*14*, *22*) and rare outside of Japan today (*23*). The three Jomon males (table S3) belong to the Y chromosome haplogroup D1b1, which is present in modern Japanese populations but almost absent in other East Asians (*24*). In contrast, the Kofun individuals all belong to mitochondrial haplogroups that are common in present-day East Asians (*25*), while the single Kofun male has the O3a2c Y chromosome haplogroup, which is also found throughout East Asia, particularly in mainland China (*26*). To place our data within the wider context of East Eurasian demography, we combined the ancient Japanese genomes with genomic data from previously published ancient (table S4 and fig. S3) and present-day individuals. Throughout this study, the modern Japanese population is represented by either data from the Simons Genome Diversity Project (SGDP) (*27*) or JPT (i.e., Japanese in Tokyo) in the 1000 Genome Project phase 3 (*28*). However, we note that ancestral heterogeneity exists across the archipelago today, which is not fully captured by this standard reference set. Other ancient and present-day populations analyzed in this study are primarily labeled by either geographic or cultural contexts.

### Genetic distinction between different cultural periods

**Fig. 2.**
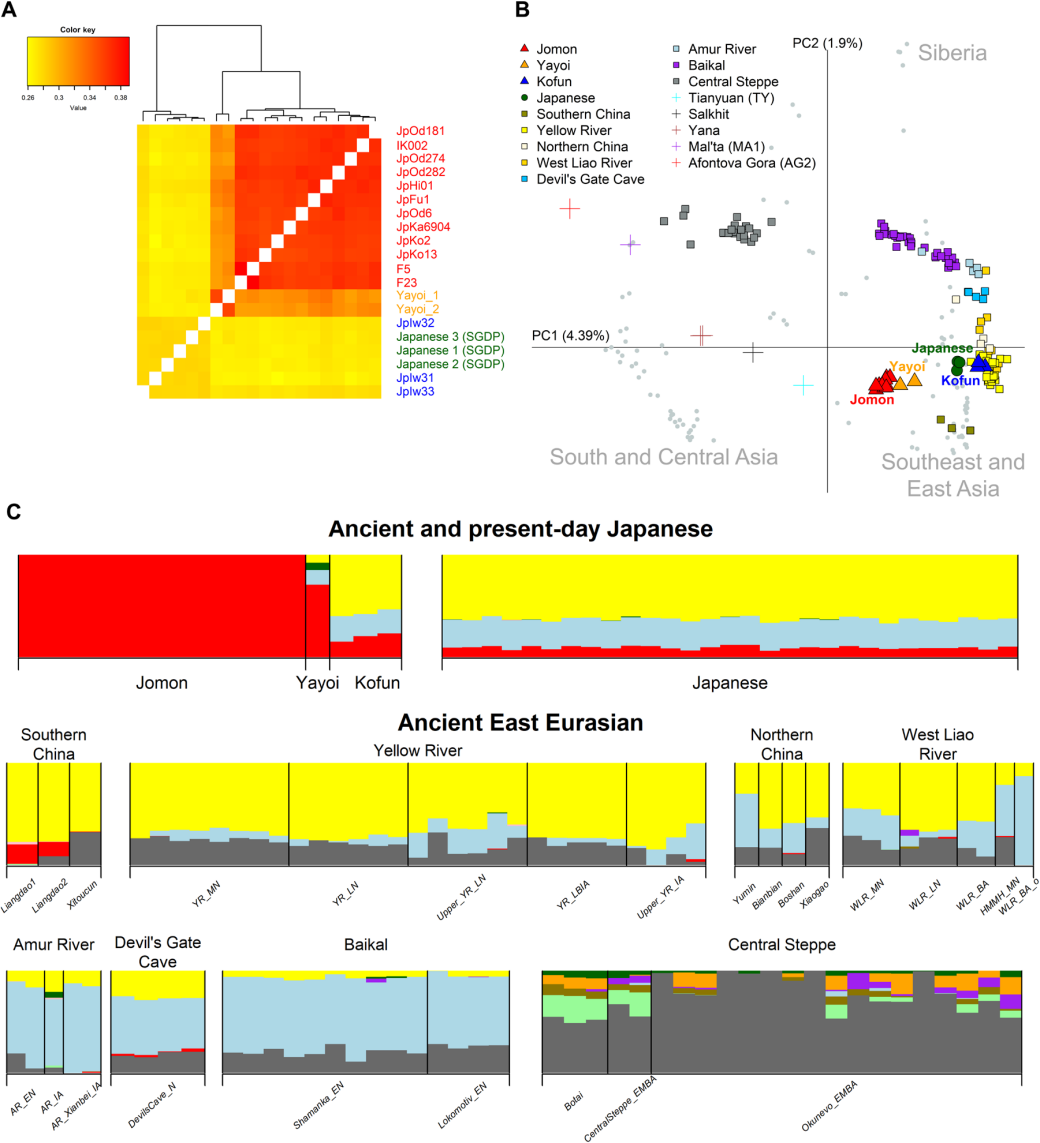
Genetic diversity through time in Japan. (**A**) A heatmap of pairwise outgroup *f*_3_ statistic comparisons between all ancient and modern Japanese individuals. (**B**) Principal components analysis (PCA) visualizing ancient Japanese individuals (i.e., Jomon, Yayoi, and Kofun) and continental ancient individuals (presented as colored symbols) projected onto 112 present-day East Eurasians (gray circles with Japanese highlighted in dark green). (**C**) Selected individuals from an ADMIXTURE analysis (*K* = 11; complete pictures from *K* = 2 to *K* = 12 are presented in figs. S5 and S6), showing a distinct Jomon ancestral component (represented by red), a component common in ancient samples from the Baikal region and the Amur River basin (represented by light blue) and a broad East Asian component (represented by yellow). A gray component that is most dominant in the Central Steppe is absent in the ancient and modern Japanese samples. The middle and bottom rows show selected Ancient East Eurasian populations from each geographical regions: Southern China (from left to right: Liangdao1, Liangdao2, and Xitoucun), Yellow River (YR) (Middle Neolithic, YR_MN; Late Neolithic, YR_LN; Late Neolithic, Upper_YR_LN; Late Bronze Age/Iron Age, YR_LBIA; and Iron Age, Upper_YR_IA), Northern China (Yumin, Bianbian, Boshan, and Xiaogao), West Liao River (WLR) (Middle Neolithic, WLR_MN; Late Neolithic, WLR_LN; Bronze Age, WLR_BA; Middle Neolithic individual from Haminmangha, HMMH_MN; and Bronze Age individual with a different genetic background from WLR_BA and WLR_BA_o), Amur River (AR) (Early Neolithic, AR_EN; and Iron Age, AR_IA and AR_Xianbei_IA), Baikal (Early Neolithic, Shamanka_EN and Lokomotiv_EN), and Central Steppe (Botai, CentralSteppe_EMBA, and Okunevo_EMBA).

We explored the genetic diversity within our time series data by looking at the shared genetic drift between all pairwise comparisons of individuals from both the ancient and modern (SGDP) Japanese populations using the statistic *f*_3_(Individual_1, Individual_2; Mbuti) (Fig. 2A). Our results very clearly define three distinct clusters of Jomon, Yayoi, and Kofun individuals, the last of whom group with the modern Japanese individuals, suggesting that cultural shifts were accompanied by genomic changes. Despite the large spatial and temporal variation in the Jomon dataset, extremely high levels of shared drift are observed between all 12 individuals. The Yayoi individuals are most closely related to each other and also have a higher affinity to the Jomon than to the Kofun individuals. The Kofun and modern Japanese individuals are almost indistinguishable from one another by this metric, implying some level of genetic continuity over the past 1400 years.

We further investigated the genome-wide autosomal affinities of our ancient Japanese individuals to continental populations using principal components analysis (PCA). We projected ancient individuals onto the genetic variation of present-day populations in the SGDP dataset from South and Central Asia, Southeast and East Asia, and Siberia (Fig. 2B and fig. S4). We observe that the ancient Japanese individuals separate into their respective cultural designations along PC1. All Jomon individuals form a tight cluster, falling apart from other ancient populations, as well as present-day Southeast and East Asians, suggesting a sustained geographic isolation. The two Yayoi individuals appear near to this Jomon cluster, supporting genetic and morphological similarity to Jomon as reported in (*15*, *16*). However, a shift toward East Asian populations implies the presence of additional continental ancestry in Yayoi. Ancient individuals from East Eurasia show a geographic cline from the south to the north on PC2: Southern China, Yellow River, Northern China, West Liao River, Devil’s Gate Cave, Amur River, and Baikal. The three individuals from the Kofun period fall within the diversity of the Yellow River cluster.

ADMIXTURE analysis with the Human Origins Array dataset also supports the increase in continental gene flow into the archipelago after the end of the Jomon period (Fig. 2C and figs. S5 and S6). The Jomon have a distinct ancestral component (represented in red in Fig. 2C), which is also visible at high levels in the Yayoi and remains at reduced levels in the Kofun and Japanese. New ancestral components appear in the Yayoi, at proportions similar to the profile seen in the Amur River basin and surrounding regions. These include a larger component that is dominant in Northeast Asians (represented by light blue) and another smaller component that represents much broader East Asian ancestry (represented by yellow). This East Asian component becomes dominant in the Kofun period and the modern Japanese population.

### Deep divergence of the Jomon lineage by geographic isolation

**Fig. 3.**
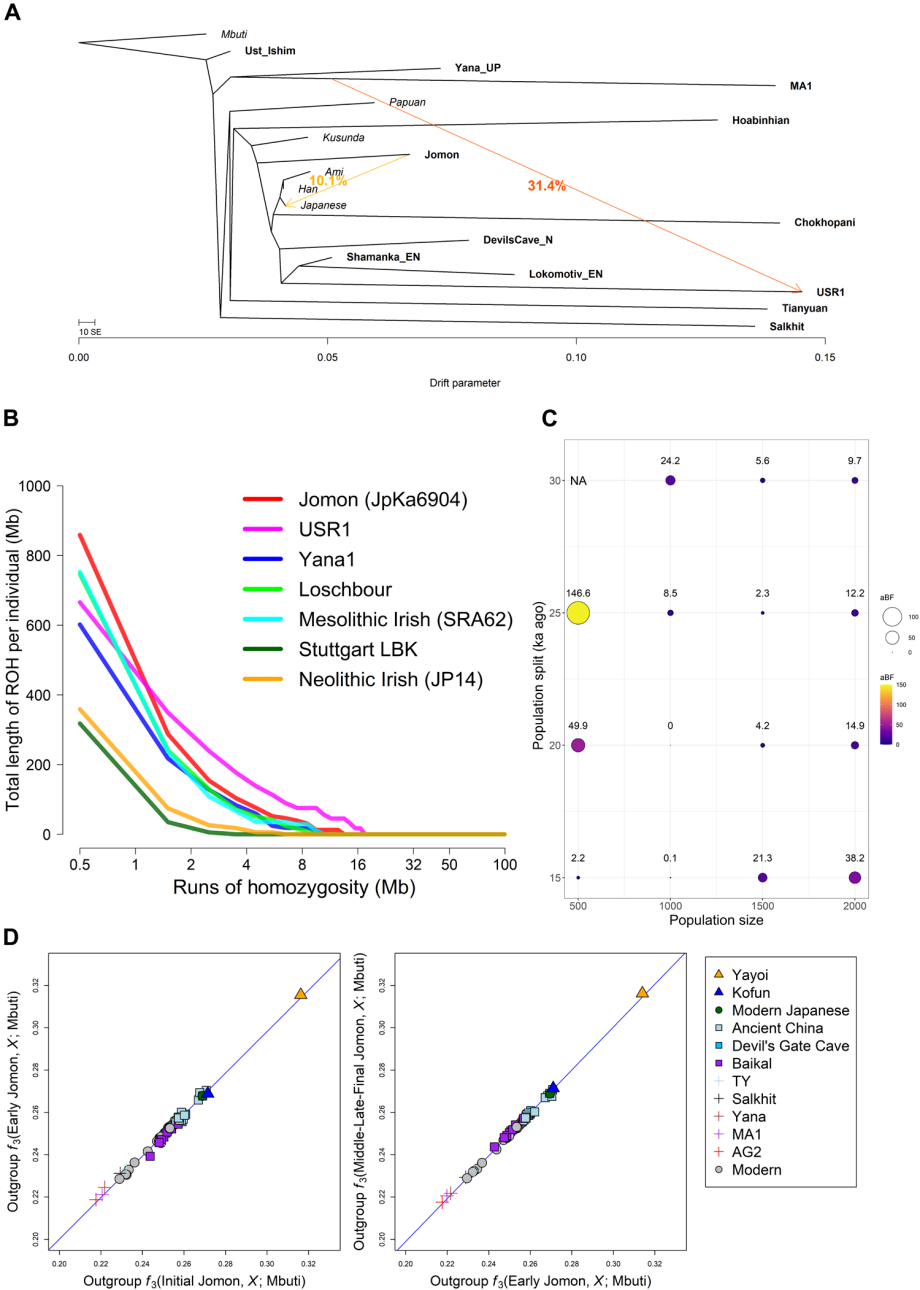
Demographic history of the Jomon lineage. (**A**) Maximum likelihood phylogenetic tree reconstructed by TreeMix under a model of two migrations. The tree shows a phylogenetic relationship among ancient (bold) and present-day (italic) populations. Colored arrows represent the migration pathways. The migration weight represents the fraction of ancestry derived from the migration edge. All other migration models from *m* = 0 to *m* = 5 are presented in fig. S7. (**B**) ROH spectra for Mesolithic and Neolithic hunter-gatherers including the 8.8-ka-old JpKa6904. Total length of ROH is plotted against different sizes of homozygous fragments ranging from 0.5 to 100 Mb. (**C**) Fitting of the models under different combinations of *N* (*x* axis) and *T* (*y* axis) for the 8.8-ka-old Jomon individual. Each point in the balloon plot represents a log_10_-scaled approximate Bayes factor (aBF) that compares likelihoods between a model with the highest likelihood and each of the other given models; the point with aBF = 0 is the model with the highest likelihood (*N* = 1000 and *T* = 20 ka ago; see fig. S10). NA means that aBF is not measurable for the model due to its likelihood equal to zero. (**D**) A comparison of outgroup *f*_3_ statistic results for the Jomon dataset divided into three subperiods measured using *f*_3_(Jomon_Sub-Period, *X*; Mbuti) (see fig. S12 for an expanded analysis). The three subperiods are as follows: Initial Jomon (JpKa6906); Early Jomon (JpFu1, JpOd6, JpOd181, JpOd274, and JpOd282); and a merged group for all Middle, Late, and Final Jomon (F5, F23, IK002, JpHi01, JpKo2, and JpKo13).

The separation of the Jomon from other populations (Fig. 2) supports the idea that they form a distinct lineage among East Eurasians, as proposed in previous studies (*13*, *14*). To explore the depth of this divergence, we reconstructed the phylogenetic relationship of Jomon with 17 other ancient and present-day populations using TreeMix with differing numbers of admixture events (Fig. 3A and fig. S7) (*29*). Our results infer that Jomon emerged after the early divergences of Upper Paleolithic East Eurasians (Tianyuan and Salkhit) and ancient Southeast Asian hunter-gatherers (Hoabinhian), but before the splitting off of other samples including present-day East Asians, an ancient Nepali (Chokhopani), hunter-gatherers from Baikal (Shamanka_EN and Lokomotiv_EN) and Chertovy Vorota Cave (Devil’s Gate Cave) in the Primorye Region, and a Pleistocene Alaskan (USR1). We further confirm the position of Jomon between two other deeply diverged hunter-gatherer lineages in this tree through formal tests of symmetric models using *f*_4_(Mbuti, *X*; Hoabinhian/DevilsCave_N, Jomon) (fig. S8, A and B). These show that all East Asian individuals in our dataset since the beginning of the Jomon period have a higher affinity to Jomon than the earlier diverged Hoabinhian but have a lower affinity in comparison to DevilsCave_N; this supports the inferred phylogeny of three distinct hunter-gatherer lineages in East Eurasia, rather than a previously proposed model in which Jomon is a mixture of Hoabinhian and East Asian-related lineages [*f*_3_(Jomon; Hoabinhian, DevilsCave_N) = 0.193, *Z* = 61.355; table S5] (*12*, *30*). We also consistently infer gene flow from Jomon to the modern Japanese across all migration models tested, with genetic contributions ranging from 8.9 to 11.5% (fig. S7). This is consistent with the mean Jomon component of 9.31% in the present-day Japanese individuals estimated from our ADMIXTURE analysis (Fig. 2C). These results suggest a deep divergence of the Jomon and an ancestral link to present-day Japanese populations.

We apply population genetic modeling to estimate the timing of the appearance of the Jomon lineage. Our approach makes use of genome-wide patterns of runs of homozygosity (ROHs) to identify the demographic scenario that best fits the ROH spectrum observed in our oldest and highest coverage sample, JpKa6904 (see note S2). The distribution of ROH tracts reflects the effective population size and the time to the most recent common ancestor between two copies of haplotypes within an individual (*31*, *32*). The 8.8-ka-old Jomon carries high levels of ROH, in particular with the highest frequency of short ROH (due to population effects rather than recent inbreeding) yet reported (Fig. 3B) (*33*). This pattern, coupled with strong shared genetic drift among the Jomon individuals (fig. S9), implies that the Jomon population underwent a severe population bottleneck. With a search of parameter space of population size and split time, our estimates place the appearance of the Jomon lineage between 15 and 20 ka ago, followed by the maintenance of a very small population size of ~1000 until at least the Initial Jomon period (Fig. 3C and figs. S10 and S11). This coincides with rising sea levels and the severing of the land bridge to the mainland at the end of the LGM (*34*, *35*) and shortly precedes the first appearance of Jomon pottery in the archipelago (*2*).

We then asked whether the Jomon had any contact with continental Upper Paleolithic people after the divergence of their lineage, but before their isolation in the archipelago, using the statistic *f*_4_(Mbuti, *X*; Jomon, Han/Dai/Japanese) (fig. S8, C to E). Among the Upper Paleolithic individuals tested, only Yana_UP is significantly closer to Jomon than Han, Dai, or Japanese, respectively (*Z* > 3.366). This affinity is still detectable even if we replace these reference populations with the other Southeast and East Asians (table S6), supporting gene flow between the ancestors of Jomon and Ancient North Siberians, a population widespread in North Eurasia before the LGM (*19*).

We lastly investigated potential temporal and spatial variation within the Jomon population. Three temporal groups, defined by the Initial, Early, and Middle-Late-Final stages of the Jomon period, show similar levels of shared genetic drift with ancient and present-day continental populations, implying little or no genetic impact from outside of the archipelago over these subperiods (Fig. 3D and fig. S12). This pattern is further backed up by the absence of any significant gene flow, observed with the statistic *f*_4_(Mbuti, *X*; sub_Jomon*_i_*, sub_Jomon*_j_*), where *i* and *j* are any pairs of the three Jomon groups (fig. S13). These Jomon individuals similarly do not display any diversity in genetic affinity to continental populations when grouped by geography (i.e., different islands on which the samples are located: Honshu, Shikoku, and Rebun Island) (fig. S14). The only observable difference within the Jomon individuals is a slightly higher affinity between sites located on Honshu, implying an insular effect with restricted gene flow between Honshu and other islands (fig. S15). Overall, these results show limited spatial and temporal genetic variation within the Jomon population, supporting the idea of near-complete isolation from the rest of Asia for thousands of years.

### Dispersal of paddy field rice farming during the Yayoi period

**Fig. 4.**
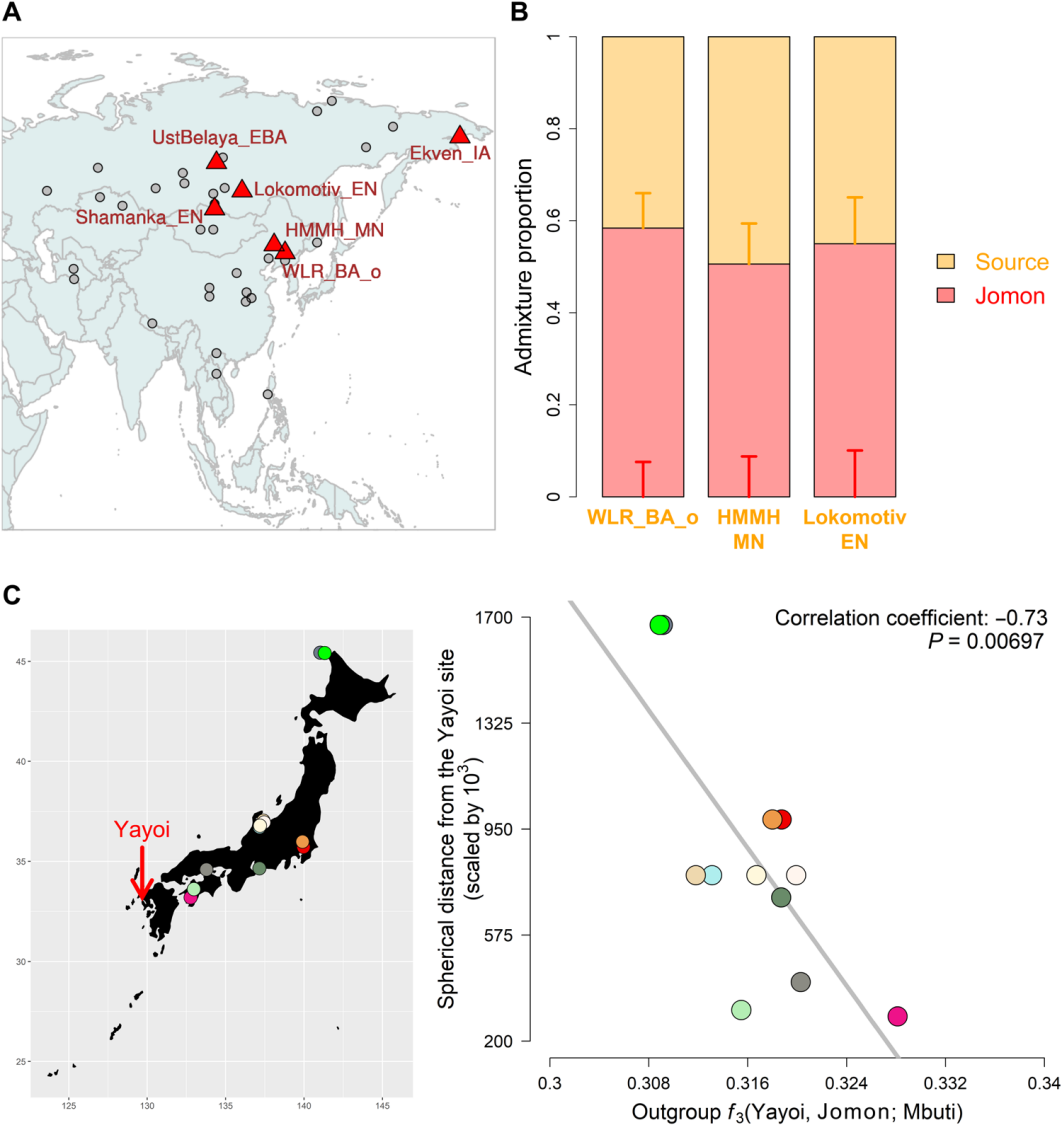
Genetic changes in the Yayoi period. (**A**) Geographical map highlighting results from *f*_4_(Mbuti, *X*; Jomon, Yayoi); continental ancient populations who are significantly closer to the Yayoi than the Jomon (with *Z* > 3.0) are represented by red triangles, while those who are symmetrically related to the both populations are represented by gray circles. (**B**) Genetic ancestry of the Yayoi modeled with a two-way admixture of the Jomon and the other source represented by: Bronze Age or Middle Neolithic individuals from West Liao River (WLR_BA_o or HMMH_MN) or Baikal hunter-gatherer (Lokomotiv_EN). Vertical bars represent ±1 SE estimated by qpAdm. The values of admixture proportions are shown in table S7. (**C**) Correlation of shared genetic drift between the Yayoi and a Jomon individual with the geographic locations of Jomon sites. The spherical distance from the Yayoi site is measured with the Haversine formula (*93*). The map marks the archaeological site of Yayoi with a red arrow and the sites of Jomon by circles with different colors.

Two individuals from the southwestern part of the archipelago associated with the Yayoi culture (Fig. 1) (*15*) were found to have both Jomon and continental ancestry (Fig. 2). Our qpAdm analysis rejected a model in which the Yayoi are unadmixed descendants of the Jomon (*P* = 0.00003), as opposed to a morphological assessment that groups these two individuals as a part of the Jomon lineage (*16*). The non-Jomon ancestral component could have been introduced by people who brought rice cultivation to the archipelago. We first tested whether any ancient East Eurasian population has higher genetic affinity to the Yayoi than Jomon using *f*_4_(Mbuti, *X*; Jomon, Yayoi) (Fig. 4A and fig. S16). Most of the sampled ancient populations in the continent do not show significant affinity to the Yayoi, including populations from the Yellow River basin (*20*) where rice farming first spread from the lower Yangtze Valley (a hypothetical origin of japonica rice, i.e., wet rice) (*36*). However, excess affinity to the Yayoi was detected in populations who had no cultural relation to rice farming (*Z* > 3.0): those from the West Liao River basin in Northeast China (WLR_BA_o and HMMH_MN), Baikal (Lokomotive_EN, Shamanka_EN, and UstBelaya_EBA), and Northeast Siberia (Ekven_IA). This affinity is not observable in two more individuals (WLR_BA; *Z* = 1.493) who came from the same archaeological site as the other Bronze Age West Liao River individual (WLR_BA_o) (*20*). These two individuals have much higher Yellow River–related ancestry (81.4 ± 6.7%) than WLR_BA_o (1.8 ± 9.1%) (*20*), which implies that the ancient Yellow River populations tested are unlikely to be a major source of the non-Jomon ancestry carried by the Yayoi.

To further distinguish these six potential sources of continental ancestry, we modeled the Yayoi as a two-way admixture of the Jomon and each in turn using qpWave (table S7). The admixture model was confidently supported (*P* > 0.05) for three of these: Baikal hunter-gatherers and the West Liao Middle Neolithic or Bronze Age individuals with a high level of Amur River ancestry (*20*). These groups all share a dominant Northeast Asian ancestral component (Fig. 2C and fig. S17) (*20*). Admixture fractions of 55.0 ± 10.1%, 50.6 ± 8.8%, or 58.4 ± 7.6% for the Jomon input were estimated by qpAdm when each of these three were, respectively, used as the second source (Fig. 4B and table S7), with a fraction of 61.3 ± 7.4% returned when the West Liao Middle Neolithic and Bronze Age individuals were merged into a single source population (table S7).

We further confirm by *f*_4_(Mbuti, Jomon; Yayoi_1, Yayoi_2) that the level of Jomon ancestry is comparable between these two Yayoi individuals (*Z* = 1.309). These results imply an approximately equal ratio of indigenous hunter-gatherer and migrant contribution to the Yayoi communities associated with this northwestern Kyushu site. This parity is particularly notable when compared to agricultural migrations in western Eurasia, where minimal hunter-gatherer contribution is observed in many regions, including the archipelago of Britain and Ireland (*37*–*40*), which mirrors Japan as an insular geographic extreme of Eurasia.

While the West Liao populations used in our admixture models did not themselves practice rice farming, they are situated just north of a hypothesized route of agricultural spread into Japan, to which our results lend weight. This follows the Shandong Peninsula (northeastern China) into the Liaodong Peninsula (northwestern part of the Korean Peninsula) and then reaches the archipelago via the Korean Peninsula (*41*).

We further investigated how the Yayoi culture spread into the archipelago using outgroup *f*_3_ statistics that measured genetic affinity between the Yayoi and each of the Jomon individuals. We find that the strength of shared genetic drift has a significant correlation with the distance from the location of the Yayoi individuals (*P* = 0.00697; Fig. 4C); the closer the Jomon archaeological sites are to the Yayoi site, the more the Jomon individuals share genetic drift with the Yayoi. This result supports the introduction of rice farming via the Korean Peninsula (*41*, *42*), followed by admixture with local Jomon populations in the south of the archipelago.

### Genetic ancestry of migrants during the Kofun period

**Fig. 5.**
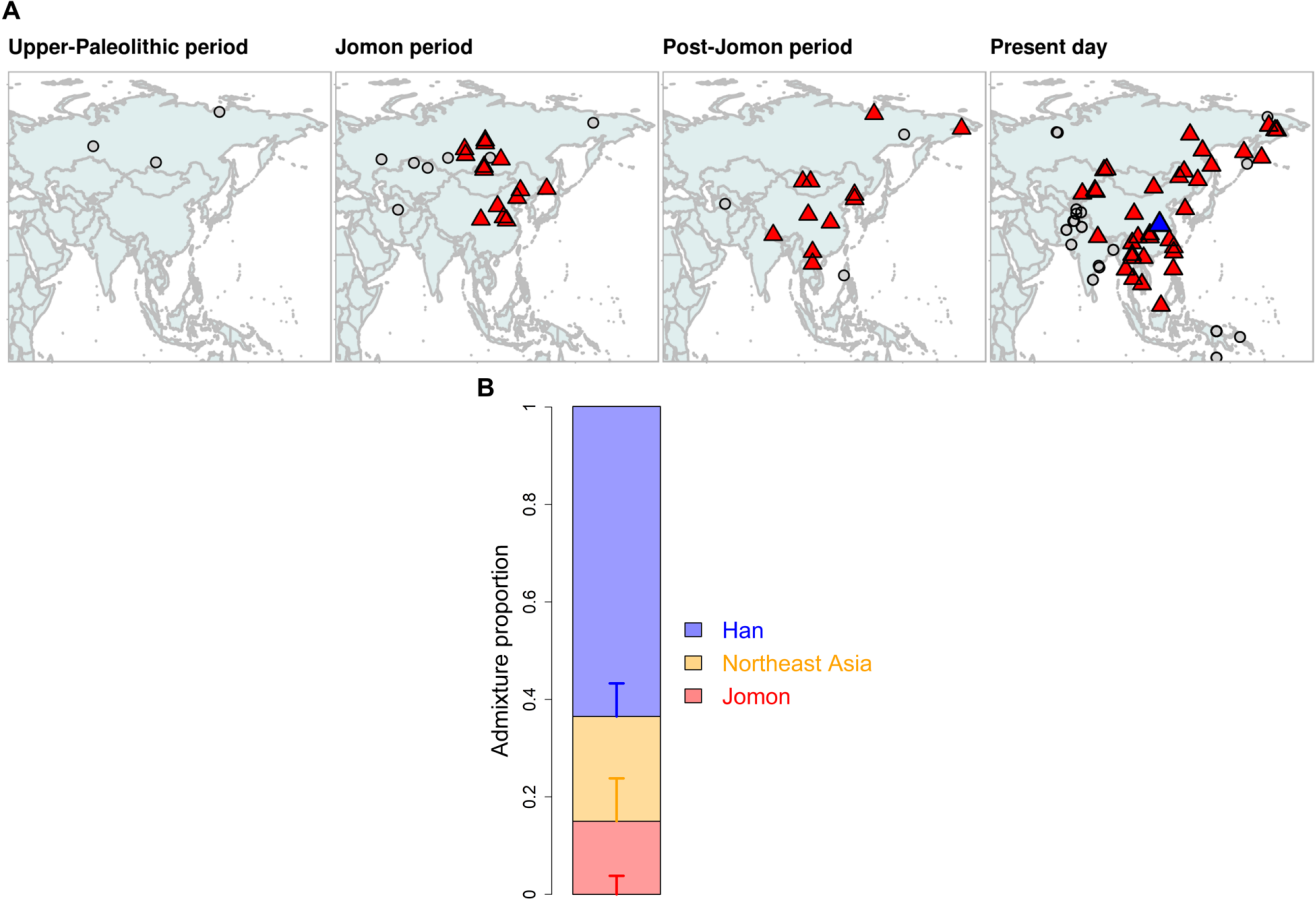
Genetic changes in the Kofun period. (**A**) Geographical maps highlighting results from *f*_4_(Mbuti, *X*; Yayoi, Kofun); continental ancient and present-day populations who are significantly closer to the Kofun than the Yayoi with a *Z* score of >3.0 are represented by red triangles, while those that are symmetrically related to both populations are represented by gray circles. The populations tested are split into four different periods, depending on their ages: Upper-Paleolithic (>16,000 years B.P.), Jomon (from 16,000 to 3000 years B.P.), Post-Jomon (from 3000 years B.P. to the present), and present day. Han is highlighted by a blue triangle in the present-day panel. (**B**) Genetic ancestry of the Kofun individuals modeled with a three-way admixture of the Jomon, Northeast Asia (WLR_BA_o and HMMH_MN), and Han. Vertical bars represent ±1 SE estimated by qpAdm. The values of admixture proportions are shown in table S10.

Historical records provide strong support for continued population movement from the continent to the archipelago during the Kofun period (*1*). However, our qpWave modeling of the three Kofun individuals rejected the two-way admixture of Jomon and Northeast Asian ancestry that fitted the Yayoi individuals (*P* < 0.05; table S8). Thus, the Kofun are genetically distinct from the Yayoi in terms of their ancestral components, as supported from our outgroup *f*_3_, PCA, and ADMIXTURE clusters (Fig. 2), as well as from previous morphological studies (*43*, *44*). To identify additional ancestral groups that contributed to the genetic makeup of the Kofun individuals, we tested genetic affinity between the Kofun and each of the continental populations using *f*_4_(Mbuti, *X*; Yayoi, Kofun) (Fig. 5A and fig. S18). We find that most of the ancient or modern populations in our dataset are significantly closer to the Kofun than they are to the Yayoi. This finding implies additional migration to the archipelago during the six centuries that separates the genomes from these two periods.

We attempted to narrow the source of this migration by testing the fit of two-way admixtures of the Yayoi and populations identified from our *f*_4_ statistics as being significantly closer to the Kofun (table S9). This admixture model was confidently supported with *P* > 0.05 for only 5 of 59 populations tested. We then applied qpAdm to quantify the genetic contribution from Yayoi and each of these sources in turn (table S9). The two-way admixture model was subsequently rejected in an additional two populations due to a lack of support from different reference sets. The remaining three populations (Han, Korean, and YR_LBIA) show 20 to 30% contributions to the Kofun (fig. S19). These three all share strong genetic drift (highlighted by the green square in fig. S17), characterized with a major component of broadly East Asian ancestry in their ADMIXTURE profiles (fig. S6). To further screen the source of additional ancestry in the Kofun individuals, we tested a three-way admixture by replacing the Yayoi ancestry with Jomon and Northeast Asian ancestry (table S10). Only Han were successfully modeled as a source of ancestry in the model (Fig. 5B), with a significantly better fitting of the three-way admixture than any possible two-way admixture models (table S11). Given that Jomon ancestry is diluted by approximately a factor of 4 between the Yayoi and Kofun populations sampled, these results suggest that the state formation phase saw a large influx of migrants who had East Asian ancestry.

We then explore the possibility that the continental ancestry observed in both the Yayoi and Kofun periods derives from the same source, with intermediate levels of Northeast and East Asian ancestry (table S12). Only one candidate was found to better fit a two-way mixture for Kofun, a population of the Late Bronze Age and Iron Age individuals from the Yellow River basin (YR_LBIA) (*20*), although this was not consistent across the reference sets (nested, *P* = 0.100; table S13). Despite not showing statistically significant gene flow with Yayoi to the exclusion of Jomon (*Z* = 2.487) (Fig. 4A and fig. S16), we find that a two-way model between YR_LBIA and Jomon also fits the Yayoi (table S14). This Yellow River population has an intermediate genetic profile with approximately 40% Northeast Asian and 60% East Asian (i.e., Han) ancestry, as estimated by qpAdm. Thus, this is an intermediate genetic profile that fits both Yayoi and Kofun in certain models, with an input of 37.4 ± 1.9% and 87.5 ± 0.8% in these populations (table S14). These results imply that continuous gene flow from a single source may be sufficient to explain the genetic changes between the Yayoi and Kofun.

However, our broader analyses strongly suggest that a single source of gene flow is less likely than two distinct waves of migrations. First, the ratios of Northeast Asian to East Asian ancestry identified in ADMIXTURE were starkly different between the Yayoi (1.9:1) and the Kofun (1:2.5) (Fig. 2C). Second, this contrast in continental affinity is also observable in the different forms of *f* statistics, in which there is a repeated pattern of Yayoi having significant affinity to those with Northeast Asian ancestry (Fig. 4A and fig. S16), while the Kofun individuals form a tight cluster with other East Asians including Han and ancient Yellow River populations (Fig. 5A and figs. S17 and S18). Last, we find support for a two-pulse model from our dating of the admixture in the Kofun individuals by DATES (fig. S20) (*45*). A single admixture event with the intermediate population (i.e., YR_LBIA) is estimated to have occurred 1840 ± 213 years before the present (B.P.), which is much later than the onset of the Yayoi period (~3 ka ago). In contrast, if two separate admixture events with two distinct sources are assumed, the resulting estimates reasonably fit the timings consistent with the beginning of the Yayoi and Kofun periods (3448 ± 825 years B.P. for the admixture between Jomon and Northeast Asian ancestry and 1748 ± 175 years B.P. for Jomon and East Asian ancestry; fig. S20). These genetic findings are further supported by both the archaeological evidence and the historical records, which document the arrival of new people from the continent during the period (*1*).

### Genetic heritage of Kofun in present-day Japanese

The three Kofun individuals are genetically similar to the present-day Japanese, as shown in Fig. 2. This implies no substantial change in the genetic makeup of Japanese populations since the Kofun period. To look for signals of additional genetic ancestry in the present-day Japanese samples, we tested whether the continental populations have preferential affinity to modern genomes relative to Kofun using *f*_4_(Mbuti, *X*; Kofun, Japanese) (fig. S21). Although some of the ancient populations exhibit higher affinity to Kofun than Japanese, none of them is supported by qpAdm as an additional source of ancestry present in the Kofun (see note S3 and table S15). Unexpectedly, no ancient or modern population shows additional gene flow with the present-day Japanese to the exclusion of the Kofun. Our admixture modeling further confirms that the present-day Japanese population is sufficiently explained by Kofun ancestry without increasing Jomon or Yayoi ancestry or without introducing an additional ancestor represented by present-day Southeast or East Asians or Siberians (table S16). We also find that the modern Japanese population has the same set of ancestral components as the three-way admixture in the Kofun individuals (table S17), with a slightly increased level of East Asian ancestry in the modern Japanese compared with the Kofun individuals sampled (fig. S22). This suggests a certain level of genetic continuity but not absolute. A strict model of continuity between the Kofun and the modern Japanese population (i.e., with no genetic drift unique to the Kofun lineage) is rejected (table S18) (*46*). However, we note no dilution of Jomon ancestry in the Japanese population (15.0 ± 3.8%), relative to the Kofun individuals (13.1 ± 3.5%) (fig. S22), as opposed to the preceding Yayoi and Kofun periods where Jomon ancestry significantly decreased due to the continental migrations (Figs. 4 and 5). Testing the genetic cladality between Kofun and Japanese by qpAdm with a “no admixture” model, we found that Kofun forms a clade with Japanese (*P* = 0.769). These results suggest that the genetic profile of three major ancestral components established by the state formation period has become a foundation for present-day Japanese populations, as also supported from dental and nonmetrical cranial traits (*47*, *48*).

## DISCUSSION

**Fig. 6.**
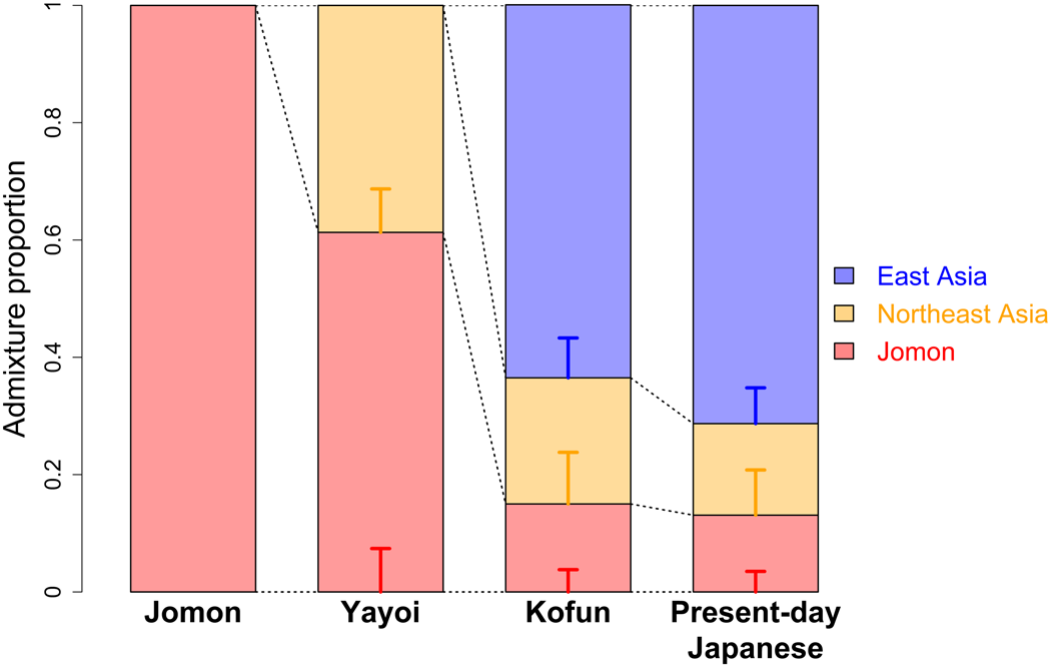
Genomic transitions in parallel with cultural transitions in pre- and protohistoric Japan. The Jomon had a very unique genetic profile due to strong genetic drift and a long-term isolation in the Japanese archipelago. The rice cultivation was brought by the people who had Northeast Asian ancestry (represented by WLR_BA_o and HMMH_MN) in the Yayoi period. An additional wave of migration brought widespread East Asian ancestry (represented by Han) to the archipelago in the Kofun period. Since then, this tripartite ancestry structure has been maintained in the archipelago and become the genetic foundation of modern Japanese.

Our data provide evidence of a tri-ancestry structure for present-day Japanese populations (Fig. 6), refining the established dual-structure model of admixed Jomon and Yayoi origins (*5*). The Jomon accumulated their own genetic variation due to long-term isolation and strong genetic drift within the Japanese archipelago following the LGM, which underlies a unique genetic component within the modern Japanese. The Yayoi period marks the end of this isolation, with substantial population migration from mainland Asia beginning at least 2.3 ka ago. However, we find a clear genetic distinction between the groups of people who arrived to the archipelago during the subsequent agrarian and state formation phases of Japanese pre- and protohistory. Genetic data from the Yayoi individuals document the presence of Northeast Asian ancestry in the archipelago as supported from morphological studies (*5*, *49*), while we observe widespread East Asian ancestry in the Kofun. The ancestors characterizing each of the Jomon, Yayoi, and Kofun cultures made a significant contribution to the formation of Japanese populations today.

The lineage ancestral to Jomon is proposed to have originated in Southeast Asia with a deep divergence from other ancient and present-day East Asians (*12*–*14*). The timing of this divergence was previously estimated to be between 18 and 38 ka ago (*14*); our modeling with the ROH profile of the 8.8-ka-old Jomon individual narrows this date to a lower limit within the range of 20 to 15 ka ago (Fig. 3). The Japanese archipelago had become accessible through the Korean Peninsula at the beginning of the LGM (28 ka ago) (*34*), enabling population movements between the continent and archipelago. The subsequent widening of the Korea Strait 17 to 16 ka ago due to rising sea levels may have led to the isolation of the Jomon lineage from the rest of the continent and also coincides with the oldest evidence of Jomon pottery production (*2*). Our ROH modeling also shows that the Jomon maintained a small effective population size of ~1000 during the Initial Jomon period, and we observe very little changes to their genomic profile in subsequent periods or across the different islands of the archipelago.

The spread of agriculture is often marked by population replacement, as documented in the Neolithic transition throughout most of Europe, with only minimal contributions from hunter-gatherer populations observed in many regions (*37*–*40*). However, we find genetic evidence that the agricultural transition in prehistoric Japan involved the process of assimilation, rather than replacement, with almost equal genetic contributions from the indigenous Jomon and new immigrants at the Kyushu site (Fig. 4). This implies that at least some parts of the archipelago supported a Jomon population of comparable size to the agricultural immigrants at the beginning of the Yayoi period, as it is reflected in the high degree of sedentism practiced by some Jomon communities (*50*–*53*).

The continental component inherited by the Yayoi is best represented in our dataset by the Middle Neolithic and Bronze Age individuals from the West Liao River basin with a high level of Amur River ancestry (i.e., WRL_BA_o and HMMH_MN) (*20*). Populations from this region are genetically heterogeneous in time and space (*20*). The Middle-to-Late Neolithic transition (i.e., between 6.5 and 3.5 ka ago) is characterized with an increase in Yellow River ancestry from 25 to 92% but a decrease in Amur River ancestry from 75 to 8% over time, which can be linked to an intensification of millet farming (*20*). However, the population structure changes again in the Bronze Age, which started around 3.5 ka ago, due to an apparent influx of people from the Amur River basin (fig. S17) (*20*). This coincides with the beginning of intensive language borrowing between Transeurasian and Sinitic linguistic subgroups (*54*). Excess affinity to the Yayoi is observable in the individuals who are genetically close to ancient Amur River populations or present-day Tunguisic-speaking populations (Fig. 4 and fig. S17). Our findings imply that wet rice farming was introduced to the archipelago by people who lived somewhere around the Liaodong Peninsula but who derive a major component of their ancestry from populations further north, although the spread of rice agriculture originated south of the West Liao River basin (*55*).

The most noticeable archaeological characteristic of Kofun culture is the custom of burying the elite in keyhole-shaped mounds, the size of which reflect hierarchical rank and political power (*1*). The three Kofun individuals sequenced in this study were not buried in those tumuli (see note S1), which suggests that they were lower-ranking people. Their genomes document the arrival of people with majority East Asian ancestry to Japan and their admixture with the Yayoi population (Fig. 5 and fig. S17). This additional ancestry is best represented in our analysis by Han, who have multiple ancestral components. A recent study has reported that people became morphologically homogeneous in the continent from the Neolithic onward (*56*), which implies that migrants during the Kofun period were already highly admixed.

Several lines of archaeological evidence support the introduction of new large settlements to Japan, most likely from the southern Korean peninsula, during the Yayoi-Kofun transition. Strong cultural and political affinity between Japan, Korea, and China is also observable from several imports, including Chinese mirrors and coins, Korean raw materials for iron production (*1*), and Chinese characters inscribed on metal implements (e.g., swords) (*57*). Access to these resources from overseas brought about intensive competition between communities within the archipelago; this facilitated political contact with polities in the continent, such as the Yellow Sea coast, for dominance (*1*). Therefore, continuous migration and continental impacts are evident throughout the Kofun period. Our findings provide strong support for the genetic exchange involved in the appearance of new social, cultural, and political traits in this state formation phase.

There are caveats to this analysis. First, we are limited to only two Late Yayoi individuals from a region where skeletal remains associated with Yayoi culture are morphologically similar to Jomon (*16*). Yayoi individuals from other regions or other time points may have different ancestral profiles, e.g., continental-like or Kofun-like ancestry. Second, our sampling is nonrandom, as is the case of our three Kofun individuals coming from the same burial site (table S1). Additional ancient genomic data will be necessary to trace temporal and regional variation in the genetic ancestry of Yayoi and Kofun populations and provide a comprehensive view of the tripartite structure of Japanese populations proposed here.

In summary, our study provides a detailed look into the changing genomic profile of the people who lived in the Japanese archipelago, both before and after agricultural and technologically driven population movements ended thousands of years of isolation from the rest of the continent. Ancient genomics on individuals from these isolated regions provides a unique opportunity to observe the magnitude of the effects of major cultural transitions on the genetic makeup of human populations.

## MATERIALS AND METHODS

### Sampling

Petrous bones and a tooth from a total of 14 ancient Japanese individuals (11 Jomon and 3 Kofun) from six archaeological sites across the west and central parts of the Japanese archipelago were sampled. Detailed information of each site is provided in note S1, including archaeological contexts and radiocarbon dates that are taken from OxCal4.4 (*58*) with the IntCal20 curve.

### DNA extraction

Sample processing was carried out in dedicated ancient DNA facilities at Kanazawa University and Trinity College Dublin where all the precautions for ancient DNA processing were followed as described in (*59*). Samples were photographed extensively before further processing. All bones were exposed to ultraviolet light for 15 min on either side to remove surface contaminants. Further cleaning of their surface was performed with a drill before extraction. A triangular wedge section of the otic capsule region of the petrous temporal bone was targeted for sampling. Half of the wedge or fragmented tooth was pulverized for DNA extraction. An aliquot of ~0.1 g of the bone powder was subjected to a silica column method with or without an initial washing step by 0.5% bleach solution as in (*60*) or by predigestion solution as in (*61*). DNA extracts were purified with MinElute silica columns (QIAGEN) and eluted at a volume of 55 μl of elution buffer.

### Library preparation and sequencing

The initial screening of each sample and blank controls was performed by constructing a double-stranded DNA next-generation sequencing library mainly using the method outlined in (*62*) with modifications as in (*38*) but also using a NEBNext Ultra II DNA Library Prep Kit for Illumina with bead-based size selection [<100 base pairs (bp)]. Every library was screened on a MiSeq Illumina platform. DNA extracts with human endogenous content of >20% were selected for high-coverage sequencing and subsequently treated with uracil-DNA-glycosylase (UDG) (*63*) before a second library construction with the former method. To increase complexity when sequencing for high-coverage, several polymerase chain reactions (PCRs) with unique indexes were prepared from each UDG-treated library. Different PCR cycles were used according to the template concentration. The concentration and quality were then assessed using the Agilent TapeStation 2200 system with the D1000 ScreenTape. High-coverage sequencing for UDG-treated libraries from 12 individuals was carried out on a HiSeq 2500 or NovaSeq 6000 Illumina platform (100-bp single-end reads or 50-bp paired-end reads) at Macrogen (Republic of Korea) or TrinSeq (Ireland).

### Sequence data processing

We trimmed adapters from raw single-end sequence reads using cutadapt v1.9.1 (*64*) with a minimum overlap of 1 bp and removed sequences shorter than 34 bp in length and from raw paired-end sequence reads using AdapterRemoval v2.2.2 (*65*) with the following parameters: --collapse --minlength 25 --minadapteroverlap 1 --minquality 25--trimns and --trimqualities. The trimmed reads were aligned to the hg19 reference genome with the revised Cambridge Reference Sequence (rCRS) for mitochondrial DNA (mtDNA) using bwa-aln in BWA (Burrows-Wheeler Aligner) v0.7.5 (*66*) with relaxed parameters “-l 16500 -n 0.01 -o 2.” We applied a mapping quality of 20 to the aligned data and removed PCR duplicates using SAMtools v1.7 (*67*). The authenticity of ancient DNA was determined in non–UDG-treated libraries by looking at degradation patterns at 5′- and 3′-end, respectively, using mapDamage2.0 (*68*). We added read groups using Picard tools version 1.101 (http://broadinstitute.github.io/picard/), before all libraries were merged into one bam per individual. Two final processing steps were carried out on the merged files: Indels were realigned using Genome Analysis Toolkit (GATK) version 3.7-0 (*69*), and the quality of the two bases at the end of each read was manually reduced to a score of 2 (“softclipped”), to avoid calling genotype from these damage-prone regions. We used the same processing pipeline for published ancient genomes in FASTQ format with the exception of using AdapterRemoval version 2.2 (*65*) instead of cutadapt; in some cases, BAM files were realigned to our reference genome and then put through the same pipeline. Quality assessment of raw data was conducted with FastQC v0.11.4 (*70*).

### Contamination estimates and determining mtDNA haplogroup

To determine the mitochondrial contamination rate and assign a mitochondrial haplogroup, we aligned sequence reads to the rCRS mitochondrial genome and reprocessed the aligned data using the same pipeline described in the previous section. Consensus sequences were determined to a minimum depth of consensus of 5 and a base quality of 30 using mpileup and bcftools within the SAMtools package. We then assigned a specific haplotype to each consensus sequence using HAPLOFIND (*71*).

We estimated contamination rates as previously described in (*33*): Briefly, we calculated the rate of secondary bases that did not match the consensus sequence at haplotype-defining and private mutations defined by HAPLOFIND. We also report the rate when removing single-nucleotide polymorphisms (SNPs) marked as C or G that may be due to postmortem damage (table S2).

### Molecular sex determination and relatedness

To determine the sex of each of our ancient samples, we filtered for reads with a minimum mapping quality of 30 and followed the method outlined in (*72*). In summary, we calculated the ratio of Y chromosome reads to the total number of sex chromosome (*R_y_*) (*72*) and assigned female if *R_y_* < 0.016 or male if *R_y_* > 0.075 (table S3). The kinship estimation for all ancient individuals was done using “Relationship Estimation from Ancient DNA” (READ) (*73*). Any first-degree relatives were removed from analysis.

### Y chromosome haplogroup analysis

All newly sequenced individuals determined to be male were piled-up using GATK version 3.7-0 (*69*) and manually compared to the International Society of Genetic Genealogy (ISOGG) SNP index with a mapping quality of 20 and a base quality of 30.

### Genotype calling and merging to published datasets

We created a large panel of ancient East Eurasians that include ancient Japanese newly sequenced in this study and published ancient genomes from Central and Eastern Steppe, Siberia, Southeast Asia, and East Asia (see a full list of ancient individuals in table S4). We genotyped all ancient individuals for the biallelic SNP sites present on two different reference panels in which they were merged to the following: (i) the Human Origin Array (HOA) consisting of 594,896 SNP sites for 1963 modern, ancient, and reference genomes (*39*); and (ii) the SGDP consisting of 278 modern, ancient, and reference genomes (*27*) that have been filtered for autosomal transversion-only SNPs with a minor allele frequency of 1%, which left 3,867,366 SNP sites in our merged data. For each SNP site, we randomly called a high-quality single base (bq30) per position to create a pseudo-diploid genotype using GATK version 3.7-0 (*69*).

### Principal components analysis

PCA was conducted using the smartpca (v16000) from the EIGENSOFT package (v7.2.0) (*74*) We projected all ancient Japanese and a subsection of other ancient individuals onto present-day East Eurasians in the SGDP panel (*n* = 112), filtered for transversions and global minor allele frequencies of 1%, with the options of “killr2: YES,” “r2thresh: 0.2,” “numoutlieriter: 0,” “lsqproject: YES,” and “autoshrink: YES.” With the exception of “Yayoi_1,” we included ancient individuals with a coverage of at least 100,000 SNPs.

### ADMIXTURE

We used ADMIXTURE v.1.3.0 (*75*) for unsupervised genetic clustering of ancient East Eurasian samples with at least 100,000 SNPs in the SGDP dataset (*n* = 189) and present-day populations from the HOA (*n* = 786; Mbuti, Sardinian, South Asians, Southeast Asians, East Asians, Siberians, Oceanians, and Native Americans). SNPs were pruned for sites in linkage disequilibrium using PLINK v1.90b4.4 (*76*), with a sliding window size of 50 variants, a step size of 5 variants, and an *r*^2^ threshold of 0.2 (--indep-pairwise 50 5 0.2), leaving 186,856 SNPs for analysis. We ran 10 replicates with random seeds for the number of clusters (*K*) from 2 to 12 and chose the run with the minimum cross-validation error for plotting.

### TreeMix analysis

Maximum likelihood trees were inferred under various admixture models using TreeMix (v1.13) (*29*). A subset of ancient and present-day populations in the SGDP dataset was chosen to represent different ancestors across East Eurasia: Ust_Ishim, Yana_UP, MA1, Tianyuan, Salkhit, Papuan, Hoabinhian (La368 and Ma911), Kusunda, Jomon, Chokhopani, Shamanka_EN, Lokomotiv_EN, DevilsCave_N, USR1, Han, Ami, and Japanese. The dataset was filtered for nonmissingness, which left 98,687 SNPs for analysis. We used Mbuti as an outgroup to root the tree and fitted models with no-migration or migrations from one to five to the data. Total 1000 to 1500 replicates were run for each model, of which the tree with a likelihood of the closest to a mean across the replicates was considered the most representative under a given model.

### *F* statistics

We calculated *f* statistics using qp3Pop (v300) and qpDstat (v662) with the *f*_4_ mode in the AdmixTools v6.0 package (*77*). We used outgroup *f*_3_ statistics to measure a genetic relationship between two populations or between all pairs of individuals within a population by specifying Mbuti as an outgroup. SEs in *f*_4_ statistics were calculated from the default block jackknife approach.

### Diploid calling and determining ROHs

We used HaplotypeCaller, part of GATK version 3.7.0, to call diploid genotypes for the oldest Jomon, JpKa6904, and other published high-coverage ancient genomes, including Yana1 (*19*), USR1 (*78*), Loschbour, Stuttgart_LBK (*39*), Mesolithic Irish (SRA62), and Neolithic Irish (JP14) (*33*). Our diploid call was based on the 1000 Genome phase 3 release v5 panel (*28*) filtered for transversion sites and a minor allele frequency of 1%. We used VCFtools v0.1.13 (*79*) to filter our results for a genotype quality of 30 and a minimum depth of 10. Transversion SNPs common to each diploid ancient sample (total of 660,027 SNPs) were used for this analysis. ROHs were measured in each diploid genome using PLINK v1.90b4.4 (*76*) with the following options: -homozyg --homozyg-density 50 --homozyg-gap 100 --homozyg-kb 500 --homozyg-snp 50 --homozyg-window-het 1 --homozyg-window-snp 50 --homozyg-window-threshold 0.05.

### Demographic modeling with ROH

To estimate the population size (*N*) and split time (*T*) of the Jomon lineage, we applied coalescent simulations of the Out-of-Africa model (*80*) to approximating a likelihood surface by fitting genome-wide patterns of ROHs to those observed in our oldest Jomon individual, JpKa6904 (see note S2). Briefly, we first measured the proportion of the Jomon genome under ROH using PLINK v1.90b4.4 (*76*). Second, we conducted coalescent simulations under different combinations of *N* and *T* using ms (*81*), with an option of “-eA” that allows sampling of ancient chromosomes at a given time in the past. We then fitted the spectrum of ROH fragments ranging from 0.5 to 100 Mb.

We broadly searched for the parameter space from all combinations of 500 ≦ *N* ≦ 2500 and 10 ≦ *T* ≦ 40 ka ago using the data from chromosome 3 to 22. Narrowing down the parameter space that includes likely scenarios, we evaluated the goodness-of-fit of the models with the full genome data (i.e., chromosomes 1 to 22). Fitting of the model was measured as an approximate Bayes factor (aBF) (*82*) where the model with the highest likelihood was compared to each of all other models. We considered log_10_-scaled aBF > 2.0 as decisive evidence on the data in favor of the highest likelihood model, as opposed to the other model (*83*).

### qpWave and qpAdm modeling

To model admixture events in the prehistory of Japan, we applied qpWave v600 and qpAdm v1000 in the AdmixTools v6.0 package to the merged datasets of ancient East Eurasians and the SGDP populations (*77*, *84*). Our analysis only used transversion sites with global minor allele frequencies of >1%, coupled with the option of “allsnps: YES.” We used a set of nine Eurasian populations, including Sardinian (*n* = 3), Kusunda (*n* = 2), Papuan (*n* = 14), Dai (*n* = 4), Ami (*n* = 2), Naxi (*n* = 3) (*27*), Tianyuan (*n* = 1) (*85*), Chokhopani (*n* = 1) (*18*), and Mal’ta (*n* = 1) (*86*), as an outgroup with or without a subset of Jomon individuals (JpKa6904, JpOd181, and IK002) in our modeling. We considered populations as sources of an admixture event only if the admixture between the sources is supported from modeling with and without a subset of Jomon in the right populations.

### Dating admixture events by DATES

We used DATES v753 (*45*) to estimate the time of two different admixture events in the Kofun individuals: one is the admixture between the Jomon and Northeast Asian ancestry, and the other is between the Jomon and East Asian ancestry. For the former model, we used the West Liao River individuals labeled as WLR_BA_O and HMMH_MN as the second source. East Asian ancestry in the latter model was represented by Han Chinese in Beijing, China (CHB) in the 1000 Genome phase 3 data (*28*). The estimated date in generation was converted into years with the assumption of 25 years per generation, which was further added into a mean age of median dates from three Kofun individuals (i.e., 1348 years before present). The parameter settings that we used are as follows: binsize: 0.001, maxdis: 1.0, runmode: 1, mincount: 1, and lovalfit: 0.45. The SE was estimated from a weighted block jackknife method.

## Supplementary Materials

### This PDF file includes:

Supplementary Text Figs. S1 to S22 Tables S1 to S18 References

## Notes

View/request a protocol for this paper from *Bio-protocol*.

## References

[1] K. Mizoguchi, *The Archaeology of Japan: From the Earliest Rice Farming Villages to the Rise of the State* (Cambridge Univ. Press, 2013).

[2] J. Habu, *Ancient Jomon of Japan* (Cambridge Univ. Press, 2004).

[3] P. U. Clark, J. D. Shakun, P. A. Baker, P. J. Bartlein, S. Brewer, E. Brook, A. E. Carlson, H. Cheng, D. S. Kaufman, Z. Liu, T. M. Marchitto, A. C. Mix, C. Morrill, B. L. Otto-Bliesner, K. Pahnke, J. M. Russell, C. Whitlock, J. F. Adkins, J. L. Blois, J. Clark, S. M. Colman, W. B. Curry, B. P. Flower, F. He, T. C. Johnson, J. Lynch-Stieglitz, V. Markgraf, J. McManus, J. X. Mitrovica, P. I. Moreno, J. W. Williams, Global climate evolution during the last deglaciation. Proc. Natl. Acad. Sci. U.S.A. 109, E1134–E1142 (2012). 22331892 10.1073/pnas.1116619109 PMC3358890

[4] E. R. Crema, J. Habu, K. Kobayashi, M. Madella, Summed probability distribution of ^14^C dates suggests regional divergences in the population dynamics of the Jomon period in Eastern Japan. PLOS ONE 11, e0154809 (2016). 27128032 10.1371/journal.pone.0154809 PMC4851332

[5] K. Hanihara, Dual structure model for the population history of the Japanese. Japan Review 2, 1–33 (1991).

[6] M. J. Hudson, S. Nakagome, J. B. Whitman, The evolving Japanese: The dual structure hypothesis at 30. Evol. Hum. Sci. 2, E6 (2020). 10.1017/ehs.2020.6 PMC10427290 37588379

[7] Japanese Archipelago Human Population Genetics Consortium, T. Jinam, N. Nishida, M. Hirai, S. Kawamura, H. Oota, K. Umetsu, R. Kimura, J. Ohashi, A. Tajima, T. Yamamoto, H. Tanabe, S. Mano, Y. Suto, T. Kaname, K. Naritomi, K. Yanagi, N. Niikawa, K. Omoto, K. Tokunaga, N. Saitou, The history of human populations in the Japanese Archipelago inferred from genome-wide SNP data with a special reference to the Ainu and the Ryukyuan populations. J. Hum. Genet. 57, 787–795 (2012). 23135232 10.1038/jhg.2012.114

[8] S. Nakagome, T. Sato, H. Ishida, T. Hanihara, T. Yamaguchi, R. Kimura, S. Mano, H. Oota; Asian DNA Repository Consortium, Model-based verification of hypotheses on the origin of modern Japanese revisited by Bayesian inference based on genome-wide SNP data. Mol. Biol. Evol. 32, 1533–1543 (2015). 25758010 10.1093/molbev/msv045

[9] T. Jinam, Y. Kawai, Y. Kamatani, S. Sonoda, K. Makisumi, H. Sameshima, K. Tokunaga, N. Saitou, Genome-wide SNP data of Izumo and Makurazaki populations support inner-dual structure model for origin of Yamato people. J. Hum. Genet. 66, 681–687 (2021). 33495571 10.1038/s10038-020-00898-3 PMC8225512

[10] T. A. Jinam, Y. Kawai, N. Saitou, Modern human DNA analyses with special reference to the inner dual-structure model of Yaponesian. Anthropol. Sci. 129, 3–11 (2021).

[11] H. Kanzawa-Kiriyama, K. Kryukov, T. A. Jinam, K. Hosomichi, A. Saso, G. Suwa, S. Ueda, M. Yoneda, A. Tajima, K.-I. Shinoda, I. Inoue, N. Saitou, A partial nuclear genome of the Jomons who lived 3000 years ago in Fukushima, Japan. J. Hum. Genet. 62, 213–221 (2017). 27581845 10.1038/jhg.2016.110 PMC5285490

[12] H. McColl, F. Racimo, L. Vinner, F. Demeter, T. Gakuhari, J. V. Moreno-Mayar, G. van Driem, U. G. Wilken, A. Seguin-Orlando, C. de la Fuente Castro, S. Wasef, R. Shoocongdej, V. Souksavatdy, T. Sayavongkhamdy, M. M. Saidin, M. E. Allentoft, T. Sato, A.-S. Malaspinas, F. A. Aghakhanian, T. Korneliussen, A. Prohaska, A. Margaryan, P. de Barros Damgaard, S. Kaewsutthi, P. Lertrit, T. M. H. Nguyen, H.-c. Hung, T. M. Tran, H. N. Truong, G. H. Nguyen, S. Shahidan, K. Wiradnyana, H. Matsumae, N. Shigehara, M. Yoneda, H. Ishida, T. Masuyama, Y. Yamada, A. Tajima, H. Shibata, A. Toyoda, T. Hanihara, S. Nakagome, T. Deviese, A.-M. Bacon, P. Duringer, J.-L. Ponche, L. Shackelford, E. Patole-Edoumba, A. T. Nguyen, B. Bellina-Pryce, J.-C. Galipaud, R. Kinaston, H. Buckley, C. Pottier, S. Rasmussen, T. Higham, R. A. Foley, M. M. Lahr, L. Orlando, M. Sikora, M. E. Phipps, H. Oota, C. Higham, D. M. Lambert, E. Willerslev, The prehistoric peopling of Southeast Asia. Science 361, 88–92 (2018). 29976827 10.1126/science.aat3628

[13] T. Gakuhari, S. Nakagome, S. Rasmussen, M. E. Allentoft, T. Sato, T. Korneliussen, B. N. Chuinneagáin, H. Matsumae, K. Koganebuchi, R. Schmidt, S. Mizushima, O. Kondo, N. Shigehara, M. Yoneda, R. Kimura, H. Ishida, T. Masuyama, Y. Yamada, A. Tajima, H. Shibata, A. Toyoda, T. Tsurumoto, T. Wakebe, H. Shitara, T. Hanihara, E. Willerslev, M. Sikora, H. Oota, Ancient Jomon genome sequence analysis sheds light on migration patterns of early East Asian populations. Commun. Biol. 3, 437 (2020). 32843717 10.1038/s42003-020-01162-2 PMC7447786

[14] H. Kanzawa-Kiriyama, T. A. Jinam, Y. Kawai, T. Sato, K. Hosomichi, A. Tajima, N. Adachi, H. Matsumura, K. Kryukov, N. Saitou, K.-I. Shinoda, Late Jomon male and female genome sequences from the Funadomari site in Hokkaido, Japan. Anthropol. Sci. 127, 83–108 (2019).

[15] K.-i. Shinoda, H. Kanzawa-Kiriyama, T. Kakuda, N. Adachi, Genetic characteristics of Yayoi people in Northwestern Kyushu. Anthropol. Sci. (Japanese Series) 127, 25–43 (2019).

[16] Y. Kaifu, K. Sakaue, R. T. Kono, Early Jomon and Yayoi human skeletal remains from Shimomotoyama Rock Shelter, Sasebo, Nagasaki prefecture, Japan. Anthropol. Sci. (Japanese Series) 125, 25–38 (2017).

[17] P. de Barros Damgaard, R. Martiniano, J. Kamm, J. V. Moreno-Mayar, G. Kroonen, M. Peyrot, G. Barjamovic, S. Rasmussen, C. Zacho, N. Baimukhanov, V. Zaibert, V. Merz, A. Biddanda, I. Merz, V. Loman, V. Evdokimov, E. Usmanova, B. Hemphill, A. Seguin-Orlando, F. E. Yediay, I. Ullah, K.-G. Sjögren, K. H. Iversen, J. Choin, C. de la Fuente, M. Ilardo, H. Schroeder, V. Moiseyev, A. Gromov, A. Polyakov, S. Omura, S. Y. Senyurt, H. Ahmad, C. McKenzie, A. Margaryan, A. Hameed, A. Samad, N. Gul, M. H. Khokhar, O. I. Goriunova, V. I. Bazaliiskii, J. Novembre, A. W. Weber, L. Orlando, M. E. Allentoft, R. Nielsen, K. Kristiansen, M. Sikora, A. K. Outram, R. Durbin, E. Willerslev, The first horse herders and the impact of early Bronze Age steppe expansions into Asia. Science 360, eaar7711 (2018). 29743352 10.1126/science.aar7711 PMC6748862

[18] C. Jeong, S. Wilkin, T. Amgalantugs, A. S. Bouwman, W. T. T. Taylor, R. W. Hagan, S. Bromage, S. Tsolmon, C. Trachsel, J. Grossmann, J. Littleton, C. A. Makarewicz, J. Krigbaum, M. Burri, A. Scott, G. Davaasambuu, J. Wright, F. Irmer, E. Myagmar, N. Boivin, M. Robbeets, F. J. Rühli, J. Krause, B. Frohlich, J. Hendy, C. Warinner, Bronze Age population dynamics and the rise of dairy pastoralism on the eastern Eurasian steppe. Proc. Natl. Acad. Sci. U.S.A. 115, E11248–E11255 (2018). 30397125 10.1073/pnas.1813608115 PMC6275519

[19] M. Sikora, V. V. Pitulko, V. C. Sousa, M. E. Allentoft, L. Vinner, S. Rasmussen, A. Margaryan, P. de Barros Damgaard, C. de la Fuente, G. Renaud, M. A. Yang, Q. Fu, I. Dupanloup, K. Giampoudakis, D. Nogués-Bravo, C. Rahbek, G. Kroonen, M. Peyrot, H. McColl, S. V. Vasilyev, E. Veselovskaya, M. Gerasimova, E. Y. Pavlova, V. G. Chasnyk, P. A. Nikolskiy, A. V. Gromov, V. I. Khartanovich, V. Moiseyev, P. S. Grebenyuk, A. Y. Fedorchenko, A. I. Lebedintsev, S. B. Slobodin, B. A. Malyarchuk, R. Martiniano, M. Meldgaard, L. Arppe, J. U. Palo, T. Sundell, K. Mannermaa, M. Putkonen, V. Alexandersen, C. Primeau, N. Baimukhanov, R. S. Malhi, K.-G. Sjögren, K. Kristiansen, A. Wessman, A. Sajantila, M. M. Lahr, R. Durbin, R. Nielsen, D. J. Meltzer, L. Excoffier, E. Willerslev, The population history of northeastern Siberia since the Pleistocene. Nature 570, 182–188 (2019). 31168093 10.1038/s41586-019-1279-z PMC7617447

[20] C. Ning, T. Li, K. Wang, F. Zhang, T. Li, X. Wu, S. Gao, Q. Zhang, H. Zhang, M. J. Hudson, G. Dong, S. Wu, Y. Fang, C. Liu, C. Feng, W. Li, T. Han, R. Li, J. Wei, Y. Zhu, Y. Zhou, C.-C. Wang, S. Fan, Z. Xiong, Z. Sun, M. Ye, L. Sun, X. Wu, F. Liang, Y. Cao, X. Wei, H. Zhu, H. Zhou, J. Krause, M. Robbeets, C. Jeong, Y. Cui, Ancient genomes from northern China suggest links between subsistence changes and human migration. Nat. Commun. 11, 2700 (2020). 32483115 10.1038/s41467-020-16557-2 PMC7264253

[21] M. A. Yang, X. Fan, B. Sun, C. Chen, J. Lang, Y.-C. Ko, C.-H. Tsang, H. Chiu, T. Wang, Q. Bao, X. Wu, M. Hajdinjak, A. M.-S. Ko, M. Ding, P. Cao, R. Yang, F. Liu, B. Nickel, Q. Dai, X. Feng, L. Zhang, C. Sun, C. Ning, W. Zeng, Y. Zhao, M. Zhang, X. Gao, Y. Cui, D. Reich, M. Stoneking, Q. Fu, Ancient DNA indicates human population shifts and admixture in northern and southern China. Science 369, 282–288 (2020). 32409524 10.1126/science.aba0909

[22] N. Adachi, K.-I. Shinoda, K. Umetsu, T. Kitano, H. Matsumura, R. Fujiyama, J. Sawada, M. Tanaka, Mitochondrial DNA analysis of Hokkaido Jomon skeletons: Remnants of archaic maternal lineages at the southwestern edge of former Beringia. Am. J. Phys. Anthropol. 146, 346–360 (2011). 21953438 10.1002/ajpa.21561

[23] M. Tanaka, V. M. Cabrera, A. M. González, J. M. Larruga, T. Takeyasu, N. Fuku, L.-J. Guo, R. Hirose, Y. Fujita, M. Kurata, K.-I. Shinoda, K. Umetsu, Y. Yamada, Y. Oshida, Y. Sato, N. Hattori, Y. Mizuno, Y. Arai, N. Hirose, S. Ohta, O. Ogawa, Y. Tanaka, R. Kawamori, M. Shamoto-Nagai, W. Maruyama, H. Shimokata, R. Suzuki, H. Shimodaira, Mitochondrial genome variation in eastern Asia and the peopling of Japan. Genome Res. 14, 1832–1850 (2004). 15466285 10.1101/gr.2286304 PMC524407

[24] M. F. Hammer, T. M. Karafet, H. Park, K. Omoto, S. Harihara, M. Stoneking, S. Horai, Dual origins of the Japanese: Common ground for hunter-gatherer and farmer Y chromosomes. J. Hum. Genet. 51, 47–58 (2006). 16328082 10.1007/s10038-005-0322-0

[25] H.-X. Zheng, S. Yan, Z.-D. Qin, Y. Wang, J.-Z. Tan, H. Li, L. Jin, Major population expansion of East Asians began before neolithic time: Evidence of mtDNA genomes. PLOS ONE 6, e25835 (2011). 21998705 10.1371/journal.pone.0025835 PMC3188578

[26] C.-C. Wang, H. Li, Inferring human history in East Asia from Y chromosomes. Investigative Genet. 4, 11 (2013). 10.1186/2041-2223-4-11 PMC3687582 23731529

[27] S. Mallick, H. Li, M. Lipson, I. Mathieson, M. Gymrek, F. Racimo, M. Zhao, N. Chennagiri, S. Nordenfelt, A. Tandon, P. Skoglund, I. Lazaridis, S. Sankararaman, Q. Fu, N. Rohland, G. Renaud, Y. Erlich, T. Willems, C. Gallo, J. P. Spence, Y. S. Song, G. Poletti, F. Balloux, G. van Driem, P. de Knijff, I. G. Romero, A. R. Jha, D. M. Behar, C. M. Bravi, C. Capelli, T. Hervig, A. Moreno-Estrada, O. L. Posukh, E. Balanovska, O. Balanovsky, S. Karachanak-Yankova, H. Sahakyan, D. Toncheva, L. Yepiskoposyan, C. Tyler-Smith, Y. Xue, M. S. Abdullah, A. Ruiz-Linares, C. M. Beall, A. Di Rienzo, C. Jeong, E. B. Starikovskaya, E. Metspalu, J. Parik, R. Villems, B. M. Henn, U. Hodoglugil, R. Mahley, A. Sajantila, G. Stamatoyannopoulos, J. T. S. Wee, R. Khusainova, E. Khusnutdinova, S. Litvinov, G. Ayodo, D. Comas, M. F. Hammer, T. Kivisild, W. Klitz, C. A. Winkler, D. Labuda, M. Bamshad, L. B. Jorde, S. A. Tishkoff, W. S. Watkins, M. Metspalu, S. Dryomov, R. Sukernik, L. Singh, K. Thangaraj, S. Pääbo, J. Kelso, N. Patterson, D. Reich, The Simons Genome Diversity Project: 300 genomes from 142 diverse populations. Nature 538, 201–206 (2016). 27654912 10.1038/nature18964 PMC5161557

[28] 1000 Genomes Project Consortium, A. Auton, L. D. Brooks, R. M. Durbin, E. P. Garrison, H. M. Kang, J. O. Korbel, J. L. Marchini, S. McCarthy, G. A. McVean, G. R. Abecasis, A global reference for human genetic variation. Nature 526, 68–74 (2015). 26432245 10.1038/nature15393 PMC4750478

[29] J. K. Pickrell, J. K. Pritchard, Inference of population splits and mixtures from genome-wide allele frequency data. PLOS Genet. 8, e1002967 (2012). 23166502 10.1371/journal.pgen.1002967 PMC3499260

[30] C.-C. Wang, H.-Y. Yeh, A. N. Popov, H.-Q. Zhang, H. Matsumura, K. Sirak, O. Cheronet, A. Kovalev, N. Rohland, A. M. Kim, S. Mallick, R. Bernardos, D. Tumen, J. Zhao, Y.-C. Liu, J.-Y. Liu, M. Mah, K. Wang, Z. Zhang, N. Adamski, N. Broomandkhoshbacht, K. Callan, F. Candilio, K. S. D. Carlson, B. J. Culleton, L. Eccles, S. Freilich, D. Keating, A. M. Lawson, K. Mandl, M. Michel, J. Oppenheimer, K. T. Özdoğan, K. Stewardson, S. Wen, S. Yan, F. Zalzala, R. Chuang, C.-J. Huang, H. Looh, C.-C. Shiung, Y. G. Nikitin, A. V. Tabarev, A. A. Tishkin, S. Lin, Z.-Y. Sun, X.-M. Wu, T.-L. Yang, X. Hu, L. Chen, H. Du, J. Bayarsaikhan, E. Mijiddorj, D. Erdenebaatar, T.-O. Iderkhangai, E. Myagmar, H. Kanzawa-Kiriyama, M. Nishino, K.-I. Shinoda, O. A. Shubina, J. Guo, W. Cai, Q. Deng, L. Kang, D. Li, D. Li, R. Lin, Nini, R. Shrestha, L.-X. Wang, L. Wei, G. Xie, H. Yao, M. Zhang, G. He, X. Yang, R. Hu, M. Robbeets, S. Schiffels, D. J. Kennett, L. Jin, H. Li, J. Krause, R. Pinhasi, D. Reich, Genomic insights into the formation of human populations in East Asia. Nature 591, 413–419 (2021). 33618348 10.1038/s41586-021-03336-2 PMC7993749

[31] F. C. Ceballos, P. K. Joshi, D. W. Clark, M. Ramsay, J. F. Wilson, Runs of homozygosity: Windows into population history and trait architecture. Nat. Rev. Genet. 19, 220–234 (2018). 29335644 10.1038/nrg.2017.109

[32] D. M. Fernandes, K. A. Sirak, H. Ringbauer, J. Sedig, N. Rohland, O. Cheronet, M. Mah, S. Mallick, I. Olalde, B. J. Culleton, N. Adamski, R. Bernardos, G. Bravo, N. Broomandkhoshbacht, K. Callan, F. Candilio, L. Demetz, K. S. D. Carlson, L. Eccles, S. Freilich, R. J. George, A. M. Lawson, K. Mandl, F. Marzaioli, W. C. McCool, J. Oppenheimer, K. T. Özdogan, C. Schattke, R. Schmidt, K. Stewardson, F. Terrasi, F. Zalzala, C. A. Antúnez, E. V. Canosa, R. Colten, A. Cucina, F. Genchi, C. Kraan, F. La Pastina, M. Lucci, M. V. Maggiolo, B. Marcheco-Teruel, C. T. Maria, C. Martínez, I. París, M. Pateman, T. M. Simms, C. G. Sivoli, M. Vilar, D. J. Kennett, W. F. Keegan, A. Coppa, M. Lipson, R. Pinhasi, D. Reich, A genetic history of the pre-contact Caribbean. Nature 590, 103–110 (2021). 33361817 10.1038/s41586-020-03053-2 PMC7864882

[33] L. M. Cassidy, R. Ó. Maoldúin, T. Kador, A. Lynch, C. Jones, P. C. Woodman, E. Murphy, G. Ramsey, M. Dowd, A. Noonan, C. Campbell, E. R. Jones, V. Mattiangeli, D. G. Bradley, A dynastic elite in monumental Neolithic society. Nature 582, 384–388 (2020). 32555485 10.1038/s41586-020-2378-6 PMC7116870

[34] J. d’Alpoim Guedes, J. Austermann, J. X. Mitrovica, Lost foraging opportunities for East Asian hunter-gatherers due to rising sea level since the Last Glacial Maximum. Geoarchaeology 31, 255–266 (2016).

[35] K. Lambeck, H. Rouby, A. Purcell, Y. Sun, M. Sambridge, Sea level and global ice volumes from the Last Glacial Maximum to the Holocene. Proc. Natl. Acad. Sci. U.S.A. 111, 15296–15303 (2014). 25313072 10.1073/pnas.1411762111 PMC4217469

[36] D. Q. Fuller, L. Qin, Y. Zheng, Z. Zhao, X. Chen, L. A. Hosoya, G.-P. Sun, The domestication process and domestication rate in rice: Spikelet bases from the Lower Yangtze. Science 323, 1607–1610 (2009). 19299619 10.1126/science.1166605

[37] P. Skoglund, H. Malmström, A. Omrak, M. Raghavan, C. Valdiosera, T. Günther, P. Hall, K. Tambets, J. Parik, K.-G. Sjögren, J. Apel, E. Willerslev, J. Storå, A. Götherström, M. Jakobsson, Genomic diversity and admixture differs for Stone-Age Scandinavian foragers and farmers. Science 344, 747–750 (2014). 24762536 10.1126/science.1253448

[38] C. Gamba, E. R. Jones, M. D. Teasdale, R. L. McLaughlin, G. Gonzalez-Fortes, V. Mattiangeli, L. Domboróczki, I. Kővári, I. Pap, A. Anders, A. Whittle, J. Dani, P. Raczky, T. F. G. Higham, M. Hofreiter, D. G. Bradley, R. Pinhasi, Genome flux and stasis in a five millennium transect of European prehistory. Nat. Commun. 5, 5257 (2014). 25334030 10.1038/ncomms6257 PMC4218962

[39] I. Lazaridis, N. Patterson, A. Mittnik, G. Renaud, S. Mallick, K. Kirsanow, P. H. Sudmant, J. G. Schraiber, S. Castellano, M. Lipson, B. Berger, C. Economou, R. Bollongino, Q. Fu, K. I. Bos, S. Nordenfelt, H. Li, C. de Filippo, K. Prüfer, S. Sawyer, C. Posth, W. Haak, F. Hallgren, E. Fornander, N. Rohland, D. Delsate, M. Francken, J.-M. Guinet, J. Wahl, G. Ayodo, H. A. Babiker, G. Bailliet, E. Balanovska, O. Balanovsky, R. Barrantes, G. Bedoya, H. Ben-Ami, J. Bene, F. Berrada, C. M. Bravi, F. Brisighelli, G. B. J. Busby, F. Cali, M. Churnosov, D. E. C. Cole, D. Corach, L. Damba, G. van Driem, S. Dryomov, J.-M. Dugoujon, S. A. Fedorova, I. G. Romero, M. Gubina, M. Hammer, B. M. Henn, T. Hervig, U. Hodoglugil, A. R. Jha, S. Karachanak-Yankova, R. Khusainova, E. Khusnutdinova, R. Kittles, T. Kivisild, W. Klitz, V. Kučinskas, A. Kushniarevich, L. Laredj, S. Litvinov, T. Loukidis, R. W. Mahley, B. Melegh, E. Metspalu, J. Molina, J. Mountain, K. Näkkäläjärvi, D. Nesheva, T. Nyambo, L. Osipova, J. Parik, F. Platonov, O. Posukh, V. Romano, F. Rothhammer, I. Rudan, R. Ruizbakiev, H. Sahakyan, A. Sajantila, A. Salas, E. B. Starikovskaya, A. Tarekegn, D. Toncheva, S. Turdikulova, I. Uktveryte, O. Utevska, R. Vasquez, M. Villena, M. Voevoda, C. A. Winkler, L. Yepiskoposyan, P. Zalloua, T. Zemunik, A. Cooper, C. Capelli, M. G. Thomas, A. Ruiz-Linares, S. A. Tishkoff, L. Singh, K. Thangaraj, R. Villems, D. Comas, R. Sukernik, M. Metspalu, M. Meyer, E. E. Eichler, J. Burger, M. Slatkin, S. Pääbo, J. Kelso, D. Reich, J. Krause, Ancient human genomes suggest three ancestral populations for present-day Europeans. Nature 513, 409–413 (2014). 25230663 10.1038/nature13673 PMC4170574

[40] L. M. Cassidy, R. Martiniano, E. M. Murphy, M. D. Teasdale, J. Mallory, B. Hartwell, D. G. Bradley, Neolithic and Bronze Age migration to Ireland and establishment of the insular Atlantic genome. Proc. Natl. Acad. Sci. U.S.A. 113, 368–373 (2016). 26712024 10.1073/pnas.1518445113 PMC4720318

[41] K. Miyamoto, The spread of rice agriculture during the Yayoi period: From the Shandong Peninsula to Japanese Archipelago via Korean Peninsula. Jpn. J. Archeol. 6, 109–124 (2019).

[42] H. Nasu, A. Momohara, The beginnings of rice and millet agriculture in prehistoric Japan. Quat. Int. 397, 504–512 (2016).

[43] H. Matsumura, A microevolutional history of the Japanese people from a dental characteristics perspective. Anthropol. Sci. 102, 93–118 (1994).

[44] Y. Kitagawa, Nonmetric morphological characters of deciduous teeth in Japan: Diachronic evidence of the past 4000 years. Int. J. Osteoarchaeol. 10, 242–253 (2000).

[45] V. M. Narasimhan, N. Patterson, P. Moorjani, N. Rohland, R. Bernardos, S. Mallick, I. Lazaridis, N. Nakatsuka, I. Olalde, M. Lipson, A. M. Kim, L. M. Olivieri, A. Coppa, M. Vidale, J. Mallory, V. Moiseyev, E. Kitov, J. Monge, N. Adamski, N. Alex, N. Broomandkhoshbacht, F. Candilio, K. Callan, O. Cheronet, B. J. Culleton, M. Ferry, D. Fernandes, S. Freilich, B. Gamarra, D. Gaudio, M. Hajdinjak, É. Harney, T. K. Harper, D. Keating, A. M. Lawson, M. Mah, K. Mandl, M. Michel, M. Novak, J. Oppenheimer, N. Rai, K. Sirak, V. Slon, K. Stewardson, F. Zalzala, Z. Zhang, G. Akhatov, A. N. Bagashev, A. Bagnera, B. Baitanayev, J. Bendezu-Sarmiento, A. A. Bissembaev, G. L. Bonora, T. T. Chargynov, T. Chikisheva, P. K. Dashkovskiy, A. Derevianko, M. Dobeš, K. Douka, N. Dubova, M. N. Duisengali, D. Enshin, A. Epimakhov, A. V. Fribus, D. Fuller, A. Goryachev, A. Gromov, S. P. Grushin, B. Hanks, M. Judd, E. Kazizov, A. Khokhlov, A. P. Krygin, E. Kupriyanova, P. Kuznetsov, D. Luiselli, F. Maksudov, A. M. Mamedov, T. B. Mamirov, C. Meiklejohn, D. C. Merrett, R. Micheli, O. Mochalov, S. Mustafokulov, A. Nayak, D. Pettener, R. Potts, D. Razhev, M. Rykun, S. Sarno, T. M. Savenkova, K. Sikhymbaeva, S. M. Slepchenko, O. A. Soltobaev, N. Stepanova, S. Svyatko, K. Tabaldiev, M. Teschler-Nicola, A. A. Tishkin, V. V. Tkachev, S. Vasilyev, P. Velemínský, D. Voyakin, A. Yermolayeva, M. Zahir, V. S. Zubkov, A. Zubova, V. S. Shinde, C. Lalueza-Fox, M. Meyer, D. Anthony, N. Boivin, K. Thangaraj, D. J. Kennett, M. Frachetti, R. Pinhasi, D. Reich, The formation of human populations in South and Central Asia. Science 365, eaat7487 (2019). 31488661 10.1126/science.aat7487 PMC6822619

[46] J. G. Schraiber, Assessing the relationship of ancient and modern populations. Genetics 208, 383–398 (2018). 29167200 10.1534/genetics.117.300448 PMC5753871

[47] Y. Dodo, Y. Kawakubo, Cranial affinities of the Epi-Jomon inhabitants in Hokkaido, Japan. Anthropol. Sci. 110, 1–32 (2002).

[48] H. Matsumura, Geographical variation of dental characteristics in the Japanese of the protohistoric Kofun period. Anthropol. Sci. 98, 439–449 (1990).

[49] Y. Mizoguchi, Affinities of the protohistoric Kofun people of Japan with pre- and proto-historic asian populations. Anthropol. Sci. 96, 71–109 (1988).

[50] J. Habu, Early sedentism in East Asia: From Late Palaeolithic to early agricultural societies in insular East Asia, in *The Cambridge World Prehistory* (Cambridge Univ. Press, 2014), vol. 3, pp. 724–741.

[51] N. Matsumoto, J. Habu, A. Matsui, in *Handbook of East and Southeast Asian Archaeology*, J. Habu, P. V. Lape, J. W. Olsen, Eds. (Springer New York, 2017), pp. 437–450.

[52] K. Imamura, *Prehistoric Japan: New Perspectives on Insular East Asia* (University of Hawaii Press, 1996).

[53] R. Pearson, Debating Jomon social complexity. Asian Perspect. 46, 361–388 (2007).

[54] M. Robbeets, Proto-transeurasian: Where and when? Man in India: Int. J. Anthropol. 97, 19–46 (2017).

[55] D. Q. Fuller, L. Qin, Water management and labour in the origins and dispersal of Asian rice. World Archaeol. 41, 88–111 (2009).

[56] K. Okazaki, H. Takamuku, Y. Kawakubo, M. Hudson, J. Chen, Cranial morphometric analysis of early wet-rice farmers in the Yangtze River Delta of China. Anthropol. Sci., 210325 (2021). 10.1537/ase.240428 PMC13134063 42157864

[57] M. Shichirō, R. A. Miller, The Inariyama tumulus sword inscription. J. Jpn. Stud. 5, 405–438 (1979).

[58] C. B. Ramsey, Bayesian analysis of radiocarbon dates. Radiocarbon 51, 337–360 (2009).

[59] B. Llamas, G. Valverde, L. Fehren-Schmitz, L. S. Weyrich, A. Cooper, W. Haak, From the field to the laboratory: Controlling DNA contamination in human ancient DNA research in the high-throughput sequencing era. Sci. Technol. Archaeol. Res. 3, 1–14 (2017).

[60] S. Boessenkool, K. Hanghøj, H. M. Nistelberger, C. Der Sarkissian, A. T. Gondek, L. Orlando, J. H. Barrett, B. Star, Combining bleach and mild predigestion improves ancient DNA recovery from bones. Mol. Ecol. Resour. 17, 742–751 (2017). 27790833 10.1111/1755-0998.12623

[61] P. B. Damgaard, A. Margaryan, H. Schroeder, L. Orlando, E. Willerslev, M. E. Allentoft, Improving access to endogenous DNA in ancient bones and teeth. Sci. Rep. 5, 11184 (2015). 26081994 10.1038/srep11184 PMC4472031

[62] M. Meyer, M. Kircher, Illumina sequencing library preparation for highly multiplexed target capture and sequencing. Cold Spring Harb. Protoc. 2010, pdb.prot5448 (2010). 20516186 10.1101/pdb.prot5448

[63] N. Rohland, E. Harney, S. Mallick, S. Nordenfelt, D. Reich, Partial uracil–DNA–glycosylase treatment for screening of ancient DNA. Philos. Trans. R. Soc. Lond. B Biol. Sci. 370, 20130624 (2015). 25487342 10.1098/rstb.2013.0624 PMC4275898

[64] M. Martin, Cutadapt removes adapter sequences from high-throughput sequencing reads. EMBnet.J. 17, 10–12 (2011).

[65] M. Schubert, S. Lindgreen, L. Orlando, AdapterRemoval v2: Rapid adapter trimming, identification, and read merging. BMC. Res. Notes 9, 88 (2016). 26868221 10.1186/s13104-016-1900-2 PMC4751634

[66] H. Li, R. Durbin, Fast and accurate short read alignment with Burrows–Wheeler transform. Bioinformatics 25, 1754–1760 (2009). 19451168 10.1093/bioinformatics/btp324 PMC2705234

[67] H. Li, B. Handsaker, A. Wysoker, T. Fennell, J. Ruan, N. Homer, G. Marth, G. Abecasis, R. Durbin; 1000 Genome Project Data Subgroup, The sequence alignment/map format and SAMtools. Bioinformatics 25, 2078–2079 (2009). 19505943 10.1093/bioinformatics/btp352 PMC2723002

[68] H. Jónsson, A. Ginolhac, M. Schubert, P. L. F. Johnson, L. Orlando, mapDamage2.0: Fast approximate Bayesian estimates of ancient DNA damage parameters. Bioinformatics 29, 1682–1684 (2013). 23613487 10.1093/bioinformatics/btt193 PMC3694634

[69] A. McKenna, M. Hanna, E. Banks, A. Sivachenko, K. Cibulskis, A. Kernytsky, K. Garimella, D. Altshuler, S. Gabriel, M. Daly, M. A. DePristo, The Genome Analysis Toolkit: A MapReduce framework for analyzing next-generation DNA sequencing data. Genome Res. 20, 1297–1303 (2010). 20644199 10.1101/gr.107524.110 PMC2928508

[70] S. Andrews, FastQC: A Quality Control Tool for High Throughput Sequence Data (2010); www.bioinformatics.babraham.ac.uk/projects/fastqc/.

[71] D. Vianello, F. Sevini, G. Castellani, L. Lomartire, M. Capri, C. Franceschi, HAPLOFIND: A new method for high-throughput mtDNA haplogroup assignment. Hum. Mutat. 34, 1189–1194 (2013). 23696374 10.1002/humu.22356

[72] P. Skoglund, J. Storå, A. Götherström, M. Jakobsson, Accurate sex identification of ancient human remains using DNA shotgun sequencing. J. Archaeol. Sci. 40, 4477–4482 (2013).

[73] J. M. Monroy Kuhn, M. Jakobsson, T. Günther, Estimating genetic kin relationships in prehistoric populations. PLOS ONE 13, e0195491 (2018). 29684051 10.1371/journal.pone.0195491 PMC5912749

[74] N. Patterson, A. L. Price, D. Reich, Population structure and eigenanalysis. PLOS Genet. 2, e190 (2006). 17194218 10.1371/journal.pgen.0020190 PMC1713260

[75] D. H. Alexander, J. Novembre, K. Lange, Fast model-based estimation of ancestry in unrelated individuals. Genome Res. 19, 1655–1664 (2009). 19648217 10.1101/gr.094052.109 PMC2752134

[76] S. Purcell, B. Neale, K. Todd-Brown, L. Thomas, M. A. R. Ferreira, D. Bender, J. Maller, P. Sklar, P. I. W. de Bakker, M. J. Daly, P. C. Sham, PLINK: A tool set for whole-genome association and population-based linkage analyses. Am. J. Hum. Genet. 81, 559–575 (2007). 17701901 10.1086/519795 PMC1950838

[77] N. Patterson, P. Moorjani, Y. Luo, S. Mallick, N. Rohland, Y. Zhan, T. Genschoreck, T. Webster, D. Reich, Ancient admixture in human history. Genetics 192, 1065–1093 (2012). 22960212 10.1534/genetics.112.145037 PMC3522152

[78] J. V. Moreno-Mayar, B. A. Potter, L. Vinner, M. Steinrücken, S. Rasmussen, J. Terhorst, J. A. Kamm, A. Albrechtsen, A.-S. Malaspinas, M. Sikora, J. D. Reuther, J. D. Irish, R. S. Malhi, L. Orlando, Y. S. Song, R. Nielsen, D. J. Meltzer, E. Willerslev, Terminal Pleistocene Alaskan genome reveals first founding population of Native Americans. Nature 553, 203–207 (2018). 29323294 10.1038/nature25173

[79] P. Danecek, A. Auton, G. Abecasis, C. A. Albers, E. Banks, M. A. DePristo, R. E. Handsaker, G. Lunter, G. T. Marth, S. T. Sherry, G. McVean, R. Durbin; 1000 Genomes Project Analysis Group, The variant call format and VCFtools. Bioinformatics 27, 2156–2158 (2011). 21653522 10.1093/bioinformatics/btr330 PMC3137218

[80] R. N. Gutenkunst, R. D. Hernandez, S. H. Williamson, C. D. Bustamante, Inferring the joint demographic history of multiple populations from multidimensional SNP frequency data. PLOS Genet. 5, e1000695 (2009). 19851460 10.1371/journal.pgen.1000695 PMC2760211

[81] R. R. Hudson, Generating samples under a Wright–Fisher neutral model of genetic variation. Bioinformatics 18, 337–338 (2002). 11847089 10.1093/bioinformatics/18.2.337

[82] N. Osada, S. Nakagome, S. Mano, Y. Kameoka, I. Takahashi, K. Terao, Finding the factors of reduced genetic diversity on X chromosomes of *Macaca fascicularis*: Male-driven evolution, demography, and natural selection. Genetics 195, 1027–1035 (2013). 24026095 10.1534/genetics.113.156703 PMC3813834

[83] R. E. Kass, A. E. Raftery, Bayes factors. J. Am. Stat. Assoc. 90, 773–795 (1995).

[84] W. Haak, I. Lazaridis, N. Patterson, N. Rohland, S. Mallick, B. Llamas, G. Brandt, S. Nordenfelt, E. Harney, K. Stewardson, Q. Fu, A. Mittnik, E. Bánffy, C. Economou, M. Francken, S. Friederich, R. G. Pena, F. Hallgren, V. Khartanovich, A. Khokhlov, M. Kunst, P. Kuznetsov, H. Meller, O. Mochalov, V. Moiseyev, N. Nicklisch, S. L. Pichler, R. Risch, M. A. R. Guerra, C. Roth, A. Szécsényi-Nagy, J. Wahl, M. Meyer, J. Krause, D. Brown, D. Anthony, A. Cooper, K. W. Alt, D. Reich, Massive migration from the steppe was a source for Indo-European languages in Europe. Nature 522, 207–211 (2015). 25731166 10.1038/nature14317 PMC5048219

[85] M. A. Yang, X. Gao, C. Theunert, H. Tong, A. Aximu-Petri, B. Nickel, M. Slatkin, M. Meyer, S. Pääbo, J. Kelso, Q. Fu, 40,000-year-old individual from Asia provides insight into early population structure in Eurasia. Curr. Biol. 27, 3202–3208.e9 (2017). 29033327 10.1016/j.cub.2017.09.030 PMC6592271

[86] M. Raghavan, P. Skoglund, K. E. Graf, M. Metspalu, A. Albrechtsen, I. Moltke, S. Rasmussen, T. W. Stafford Jr., L. Orlando, E. Metspalu, M. Karmin, K. Tambets, S. Rootsi, R. Mägi, P. F. Campos, E. Balanovska, O. Balanovsky, E. Khusnutdinova, S. Litvinov, L. P. Osipova, S. A. Fedorova, M. I. Voevoda, M. DeGiorgio, T. Sicheritz-Ponten, S. Brunak, S. Demeshchenko, T. Kivisild, R. Villems, R. Nielsen, M. Jakobsson, E. Willerslev, Upper Palaeolithic Siberian genome reveals dual ancestry of Native Americans. Nature 505, 87–91 (2014). 24256729 10.1038/nature12736 PMC4105016

[87] J. Habu, C. Fawcett, Jomon archaeology and the representation of Japanese origins. Antiquity 73, 587–593 (1999).

[88] Y. Nakazawa, On the Pleistocene population history in the Japanese archipelago. Curr. Anthropol. 58, S539–S552 (2017).

[89] T. Gakuhari, H. Komiya, J. Sawada, T. Anezaki, T. Sato, K. Kobayashi, S. Itoh, K. Kobayashi, H. Matsuzaki, K. Yoshida, M. Yoneda, Radiocarbon dating of one human and two dog burials from the Kamikuroiwa rock shelter site, Ehime Prefecture. Anthropol. Sci. 123, 87–94 (2015).

[90] A. Scally, R. Durbin, Revising the human mutation rate: Implications for understanding human evolution. Nat. Rev. Genet. 13, 745–753 (2012). 22965354 10.1038/nrg3295

[91] C. Jeong, A. T. Ozga, D. B. Witonsky, H. Malmström, H. Edlund, C. A. Hofman, R. W. Hagan, M. Jakobsson, C. M. Lewis, M. S. Aldenderfer, A. Di Rienzo, C. Warinner, Long-term genetic stability and a high-altitude East Asian origin for the peoples of the high valleys of the Himalayan arc. Proc. Natl. Acad. Sci. U.S.A. 113, 7485–7490 (2016). 27325755 10.1073/pnas.1520844113 PMC4941446

[92] D. Massilani, L. Skov, M. Hajdinjak, B. Gunchinsuren, D. Tseveendorj, S. Yi, J. Lee, S. Nagel, B. Nickel, T. Devièse, T. Higham, M. Meyer, J. Kelso, B. M. Peter, S. Pääbo, Denisovan ancestry and population history of early East Asians. Science 370, 579–583 (2020). 33122380 10.1126/science.abc1166

[93] R. J. Hijmans, E. Williams, C. Vennes, Geosphere: Spherical trigonometry, R package version 1 (2016).

