# Supplementary material for: Ancient genomics reveals tripartite origins of Japanese populations
Source: Sci Adv. 2021 Sep 17;7(38):eabh2419. doi: 10.1126/sciadv.abh2419 (PMC8448447; doi:10.1126/sciadv.abh2419)
Supplement: Supplementary file 1 — Supplementary Text Figs. S1 to S22 Tables S1 to S18 References [file sciadv.abh2419_sm.pdf]

Science Advances
AAAS

Supplementary Materials for

Ancient genomics reveals tripartite origins of Japanese populations

Niall P. Cooke, Valeria Mattiangeli, Lara M. Cassidy, Kenji Okazaki, Caroline A. Stokes, Shin Onbe, Satoshi Hatakeyama, Kenichi Machida, Kenji Kasai, Naoto Tomioka, Akihiko Matsumoto, Masafumi Ito, Yoshitaka Kojima, Daniel G. Bradley*, Takashi Gakuhari*, Shigeki Nakagome*

*Corresponding author. Email: dbradley@tcd.ie (D.G.B.); gakuhari@staff.kanazawa-u.ac.jp (T.G.); nakagoms@tcd.ie (S.N.)

Published 17 September 2021, *Sci. Adv.* **7**, eabh2419 (2021)
DOI: 10.1126/sciadv.abh2419

This PDF file includes:

Supplementary Text
Figs. S1 to S22
Tables S1 to S18
References

Note S1: Archaeological contexts

A brief introduction on pre- and protohistoric Japan

The Japanese archipelago consists of five main islands including Hokkaido, Honshu, Shikoku, Kyushu, and Okinawa and is a region which is extremely rich in archaeological sites (*87*). The pre- and protohistory of Japan is divided into four different periods: the Paleolithic, Jomon, Yayoi, and Kofun. The lithic-based Paleolithic, which started >38,000 years ago (ka ago) (*88*), transformed into the pottery-producing Jomon >16 ka ago (*2*). The Jomon (which translates to cord mark decorations; *Jo* meaning "cord" and *mon* meaning "mark" in Japanese) are considered to be complex hunter-gatherers (*50–53*). Characteristics of the Jomon culture include: the presence of large settlements, a high population density per site, low residential mobility, logistically organized subsistence strategies (evidenced by the presence of storage pits and large shell-middens), and the construction of large ceremonial features. The Jomon period lasted more than 10,000 years and is split into six sub-periods based on temporal variation in these cultural characteristics: Incipient (15,000-11,000 calibrated before present, calBP), Initial (11,000-7,000 calBP), Early (7,000-5,400 calBP), Middle (5,400-4,400 calBP), Late (4,400-3,300 calBP), and Final Jomon (3,300-2,300 calBP) (*51*).

The subsequent period between the beginning of wet-rice cultivation and the emergence of the custom of burying the elite in keyhole-shaped tumuli is referred to as the Yayoi period, the first agrarian phase in Japanese history (*1*). Systematic rice paddy field agriculture was introduced into the Japanese archipelago during the first millennium BC. Within approximately 1,000 years, technological advancements in tool-making led not only to further advances in rice cultivation, but also to social stratification due to increasing contradiction between the hierarchization of social relations and the necessity of maintaining communal collaboration and the egalitarian ethos for food production (*1*).

The following Kofun period is regarded as the state-formation phase as represented by the beginning of the imperial reign. A prominent feature of this period is the construction of large burial mounds, whose size reflected the social status of its occupant during their lifetime (*1*). This trend is particularly evident in archaeological sites from the early Kofun period. However, the keyhole structure slowly began to be downsized over the course of time (*1*). Coinciding with this decline in the size of large keyhole tumuli, packed tumuli clusters, such as horizontal cave tombs, became prevalent in the Late Kofun period. The construction of keyhole tumuli ceased by the end of the Kofun period (the six century AD) which marked the completion of the process whereby the elite became the rulers and managed to control inevitable hierarchies that had persisted since the introduction of paddy field rice cultivation.

In the following sections, we provide details of archaeological contexts of the sites where the samples used in this study were collected from (see Fig. 1a for geographic locations of the sites).

1. Jomon

1.1. Kamikuroiwa rock shelter (Initial Jomon; JpKa6904)

Kamikuroiwa rock shelter is located in Kumakogen, Kamiukena District, Ehime Prefecture of Shikoku. The site is sitting on a terrace along Kuma River, at an elevation of about 396 m above sea level. The excavation of this site was conducted from 1962 to 1970, identifying well-preserved deposits that were stratigraphically divided into nine layers. Most of the human burials, including JpKa6904 sequenced in this study, were recovered from Layer 4, coupled with large pieces of Jomon potteries; their roller-stamped designs (so-called "Oshigata-mon" in Japanese) associated Layer 4 with the Initial Jomon. The radiocarbon date of JpKa6904 reported in (*89*) was corrected with the IntCal20 curve, which gave 6,696-7,041 calBC (8,819 ± 172 calBP), consistent with the archaeological contexts of this layer. JpKa6904 had morphological characteristics of a female, which was further confirmed by our genetic data (see Table S3). An additional individual recovered from the same layer was screened for high-coverage sequencing but had little endogenous human DNA left in a tooth (<1%).

1.2. Odake shell-midden (Early Jomon; JpOd)

The Odake shell midden is located around 4 km inland from the present-day seacoast, at the junction of the Kureha hills in Toyama, a coastal city of north central Honshu. At the time of settlement at the Odake shell midden, the sea level was higher than the level at the present due to climatic warming and this site was located along the shore, emerging as the result of marine transgressions. At least 100 human skeletons have been discovered from this site, most of which were excavated between 2009 and 2010 due to the construction of new train lines. Buried skeletons had a specific burial practice in which the body was placed in a flexed position with bent legs. Some of the buried individuals folded stones in their arms across their chest. Animal remains excavated from this site included terrestrial and marine mammals, as well as fish, suggesting that the Jomon in this area utilized a variety of food resources. Four individuals from this site produced genomic data in this study, all of which are included in the early stage of the Early Jomon period as follows:

- JpOd274: 4,169-4,339 calBC (6,204 ± 85 calBP)
- JpOd6: 3,984-4,229 calBC (6,057 ± 123 calBP)
- JpOd181: 3,801-3,967 calBC (5,834 ± 83 calBP)
- JpOd282: 3,787-3,952 calBC (5,820 ± 83 calBP)

1.3. Funagura shell-midden (Early Jomon; JpFu)

The Funagura shell-midden is located in Kurashiki, a coastal city of western Honshu, sitting on the Seto Inland Sea that separates three main islands, Honshu, Shikoku, and Kyushu. Three burials were excavated from this site in 1991; two individuals were initially screened for human endogenous DNA; only JpFu1 was analyzed in this study due to the poor preservation of the other individual. JpFu1 was discovered in Layer 3 where the density of shell deposits was extremely high. Several Jomon potteries, together with lithic tools, were recovered from the same layer, the type of which had an association with the late stage of the Early Jomon period. Our radiocarbon dating showed 3,528-3,640 calBC (5,534 ± 56 calBP). This individual had morphological characters that were considered an adult female; our genetic analysis assigns female to this individual (Table S3). In addition to the human skeletons, animal remains were also excavated from this site, including fish (*e.g.*, sharks and Japanese black porgy), birds, and mammals (*e.g.*, deer and boars). These archaeological evidence supports that both fishing in the Inland Sea and

hunting terrestrial animals could have been central to food collection for the Jomon population in this site.

1.4. Kosaku shell-midden (Middle-to-Late Jomon; JpKo)

The Kosaku shell-midden is located in Funabashi, a coastal city of central Honshu. This site was initially discovered in 1883; following excavations identified the Jomon pottery that represents the Late Jomon period, in particular those having the lid with many large bracelets made from shell inside the pottery. Around 100 human skeletons were recovered from this site, some of which were collectively buried. Two individuals sequenced in this study, JpKo2 and JpKo13, were buried individually, dated to be the Middle or Late Jomon period (see below). There were animal remains excavated together with the human skeletons, which included deers, boars, racoons, as well as a variety of fish and shells.

- JpKo2: 2,344-2,564 calBC (4,404 ± 110 calBP; Middle Jomon)
- JpKo13: 1,897-2,028 calBC (3,913 ± 66 calBP; Late Jomon)

1.5. Hirajo shell-midden (Late Jomon; JpHi01)

The Hirajo shell-midden is located in Ainan, a coastal city of Shikoku. This site was originally discovered in 1891 and is known for a specific "Hirajo" type of the Jomon pottery, that is representative of the middle of the Late Jomon period. Hirajo-type pottery had a certain level of similarity to the pottery found in Northern Kyushu or Honshu on the Seto Inland Sea, suggesting potential trade across the areas. A total of 10 burials were recovered from this site during excavations conducted from 1954 to 1972, one of which, JpHi01, was well preserved as discovered with a full body skeleton and chosen for genomic sequencing in this study. This individual was dated to be 1,735-1,900 calBC (3,768 ± 83 calBP) (referred from the Annual Archaeological Report from Ainan Town 2020). The Late Jomon culture had a practice of ritual tooth ablation; however, this individual had no evidence on tooth removal. From morphological characters, this individual was likely to be an adult. Animal remains excavated together with the humans include deers, boars, sharks, clams, and oysters, supporting the fishing and hunting lifestyle of the Jomon in this region. Fiber remains were also found in this site, suggesting the Jomon might have made their clothes out of fibers.

2. Kofun

Iwade horizontal cave tombs (JpIw)

Horizontal cave tombs are constructed on a hill and dug in parallel to the ground. Multiple small tombs make a cluster, each of which contains a burial chamber where multiple individuals were accommodated by shifting previously buried individuals to make room for new burials. The Iwade site is located in Kanazawa, Ishikawa Prefecture of Honshu, consisting of eight tombs split into three sections (Section A: No. 1, 2, 3, 5; Section B: No. 6, 7, 8; Section C: No. 4). Three individuals sequenced in this study were excavated from the No. 3 tomb. A number of *Sue* stoneware were buried together with the human body in the tomb. The shape of this stoneware was uniquely observable in sites from eastern or northeastern Japan, but rare in northwestern Japan and completely absent in western Japan. This suggests that people might have moved to the areas such as the Iwade site from eastern parts of the archipelago. Given that a keyhole tumulus was constructed in the sixth century AD near the Iwade horizontal cave tombs, a sociopolitical community could have been newly formed in the Late Kofun period. Radiocarbon dates of our samples are consistent with the timing of the appearance of the keyhole tumulus:

- JpIw32: 541-603 AD (1,378 ± 31 calBP)
- JpIw31: 573-647 AD (1,340 ± 37 calBP)
- JpIw33: 595-655 AD (1,325 ± 30 calBP)

Note S2: Demographic modelling by runs of homozygosity (ROH)

We identify the excess accumulation of short runs of homozygosity (ROH) in our oldest Jomon genome, JpKa6904, compared to Mesolithic hunter-gatherers from Ireland (*33*) and Luxembourg (*39*), as well as the Upper-Paleolithic Northeast Siberian (*19*) and the other Neolithic or Pleistocene genomes (*33*, *39*, *78*) (see Fig. 3b). This suggests that the Jomon had a smaller population size than the West Eurasian hunter-gatherers likely due to a long-term isolation in insular East Asia. To estimate the timing of divergence of the Jomon lineage, coupled with the population size, we employed a simulation-based modelling approach that can fit genome-wide patterns of ROH expected under given demographic conditions to those observed in the Jomon genome.

We applied diploid-genotype calling to the high-coverage ancient genomes including 7.5× JpKa6904. To minimize any potential confounding effects of postmortem deamination on the ROH analysis, we called genotypes only for single nucleotide polymorphisms (SNPs) with transversions filtered for global minor allele frequencies above 1% among the Phase 3 v5 1000 Genomes release (*28*). Additional filtering steps were applied to the genotype calling: bases with a quality >30, a sequence depth >10, and a genotype quality >20. This left 984,740 autosomal SNPs for ROH analysis. We then used PLINK v1.90 (*76*) to detect ROH in the Jomon genome with the following options: --homozyg --homozyg-density 50 --homozyg-gap 100 --homozyg-kb 500 --homozyg-snp 50 --homozyg-window-het 1 --homozyg-window-snp 50 --homozyg-window-threshold 0.05. The ROH profile was summarized into a spectrum of ROH fragments ranging from 0.5 to 100 Mb with a bin size of 1 Mb (Fig. S10).

The observed ROH spectrum is shaped jointly by the population size and timing of population split. The previous study has provided estimates on the divergence of Jomon lineage from the common ancestor of Han to be in between 18 and 38 ka ago, with a constant population size that falls within the range from 2,000 to 3,000 (*14*). We first performed a broad search for the parameter space that is defined with the population size (N) from 500 to 2,500 and population split from 10 to 40 ka ago (T) using the data from chromosomes 3 to 22 (Fig. S11). To test whether the Jomon demography influences the pattern of ROH in a genome, we generated 100 whole-genome simulations, using a coalescent simulator *ms* (*81*), for different combinations of N and T under a fixed scenario of the Out-of-Africa dispersal reconstructed from a previous study (*80*) with slight modifications (Fig. S10a). Our simulation assumed 25 years per generation and 1.25×10^{-8} per generation (*90*) and 0.625×10^{-8} per generation for a mutation rate and a recombination rate respectively.

We then estimated the likelihood of observing the ROH spectrum similar to that observed in JpKa6904 (shown as a dashed red line in Fig. S10b). We applied the method developed in (*82*) that measures the similarity of summary data (*i.e.*, ROH spectrum) between the observed and simulated data based on a kernel density estimate and that calculates an approximated marginal likelihood (aML) of a given model. A normalized Gaussian kernel function with a bandwidth of 1.0 was used to compute kernel density estimates of aMLs. These estimates were then compared to identify the best-fitting model as an approximate Bayes factor (aBF); we calculated aBFs between a model with the highest aML and any other models.

Our broad search was able to confine the parameter space into $500 \leqq N \leqq 2{,}000$ and $15 \leqq T \leqq 30$ ka ago that includes likely scenarios for further testing with all autosomal chromosomes (Fig. S11). Including chromosomes 1 and 2 increased the power in discriminating the highest likelihood model from the others (Fig. 3c); the model with $N = 1{,}000$ and $T = 20$ ka ago was significantly

favoured with $\log_{10}$-scaled aBFs >2.0 against any other models, except for that with N = 1,000 and T = 15 ka ago ($\log_{10}$-scaled aBF: 0.1).

Note S3: Testing the presence of addition ancestry in the Kofun

We rigorously tested whether the Kofun have additional ancestry that is absent or reduced in modern Japanese using *qpAdm*. We modelled the Kofun and modern Japanese with a four-way admixture by adding populations identified from the f_4-statistics as significantly closer to the Kofun (Fig. S21) to the three sources of Jomon, Northeast Asian, and East Asian ancestry (Table S15). None of the populations tested support the four-way admixture models; the three-way admixture without the fourth source always better explains the genetic ancestry of Kofun than the four-way admixture (nested p-value > 0.05) (see Table S15). These results suggest that no additional ancestral component is present at any detectable level in the Kofun.

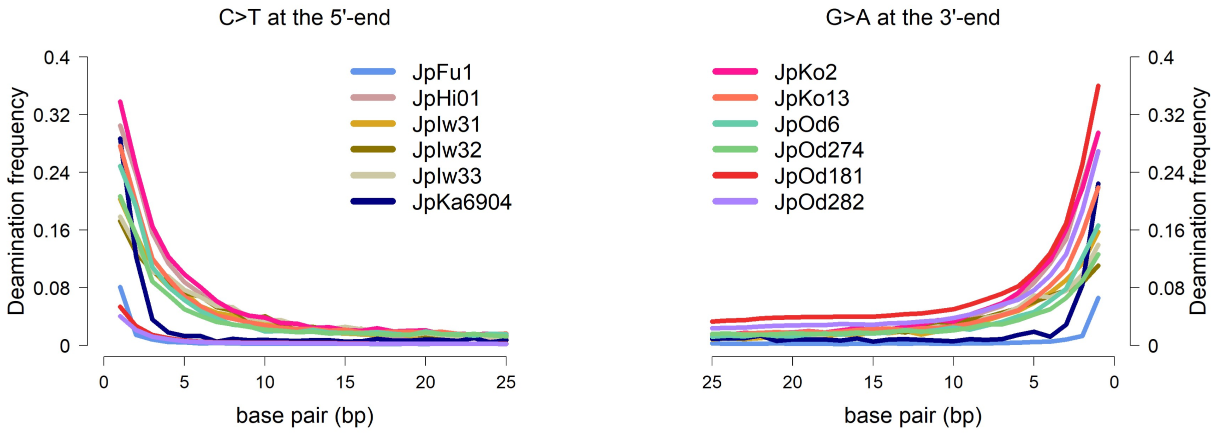

Fig. S1. DNA damage plots for newly sequenced ancient Japanese. Damage patterns on the left plot show C>T misincorporations at the 5'-end, while those on the right plots show G>A misincorporations at the 3'-end.

(a)

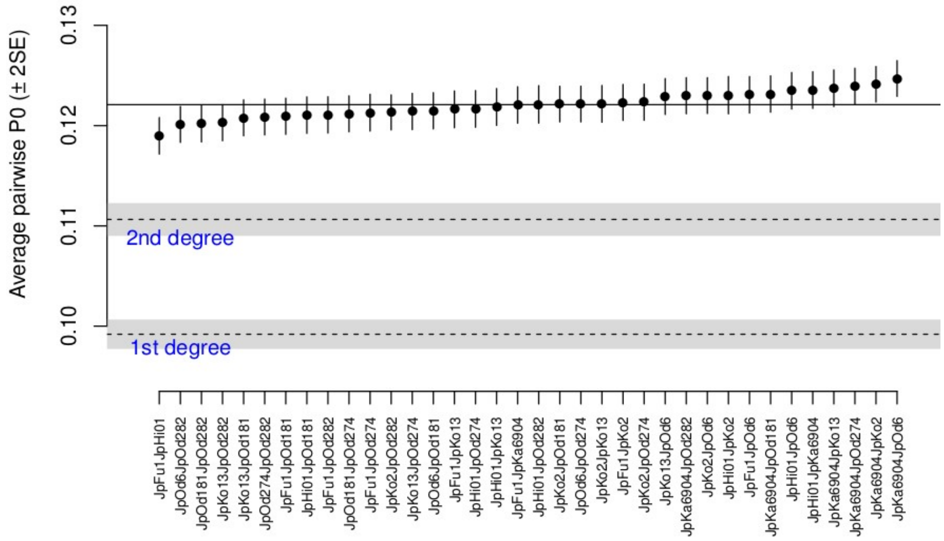

(b)

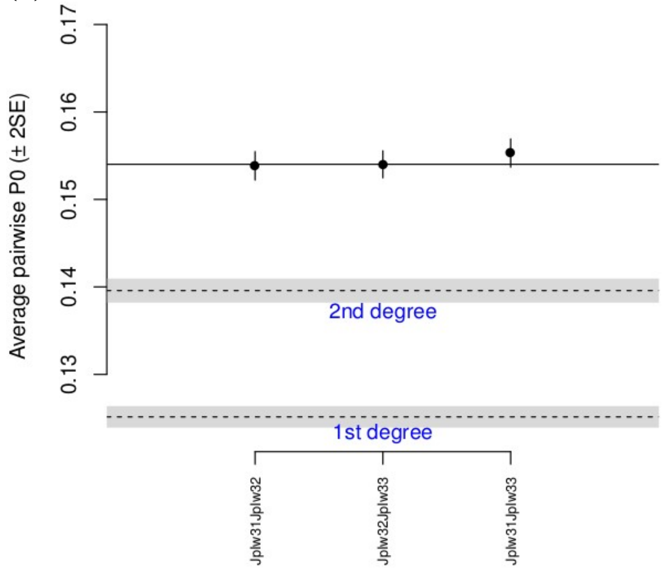

Fig. S2. Results of kinship analysis by READ for all (a) Jomon and (b) Kofun individuals sequenced in this study. Pairs of individuals tested are listed on the *x*-axis, while the *y*-axis represents average pairwise P0 scores. Dashed lines show the cut-off for the 1st and 2nd degree relatives.

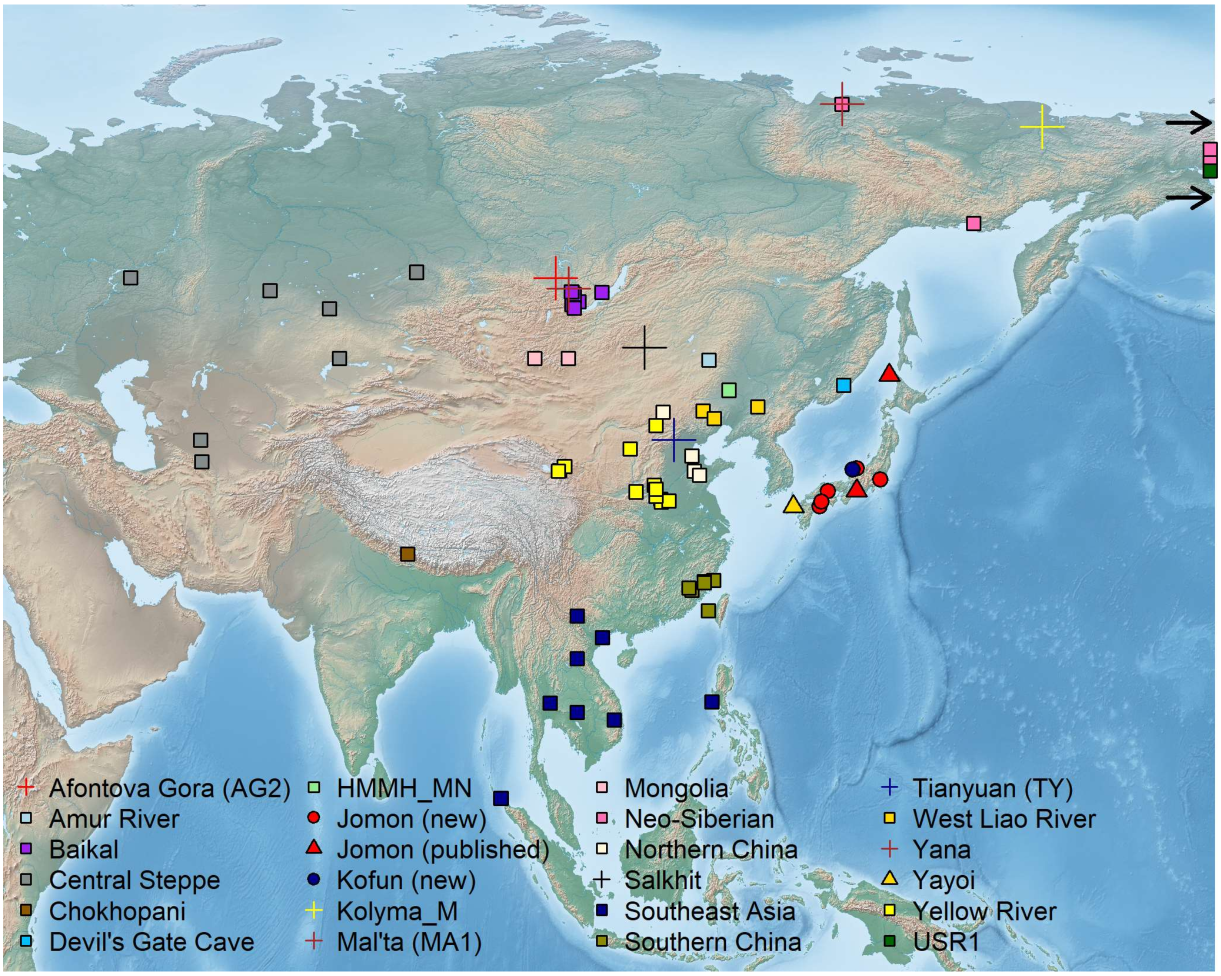

Fig. S3. A topographic map showing locations of ancient samples included in this study. Ancient data are represented by symbols designated by geographic or cultural context. Ancient Japanese newly sequenced in this study are highlighted with circles, while those previously published data are represented by triangles. Sites of Upper-Paleolithic samples are marked with crosses. Other ancient individuals are represented by coloured squares according to the following groupings: Amur River (AR_EN, AR_IA, and AR_Xianbei_IA), Baikal (Kurma_EBA, Lokomotiv_EN, Shamanka_EBA, Shamanka_EN, UstBelaya_EBA, UstBelaya_MED, UstBelaya_N, UstIda_EBA, and UstIda_LN), Central Steppe (Botai, CentralSteppe_EMBA, Namazga_CA, Okunevo_EMBA, SidelkinoEHG_ML, Turkmenistan_IA, and YamnayaKaragash_EBA), Chokhopani, Devil's Gate Cave (DevilsCave_N), HMMH_MN, Mongolia (ARS008 and ARS026), Neo-Siberian (Ekven_IA, Magadan_BA, Uelen_IA, and Yana_MED), Northern China (Bianbian, Boshan, Xiaogao, Xiaojingshan, and Yumin), Southeast Asia (McColl_Groups_1-6, McColl_Group_3.1, and McColl_Group_4.1), Southern China (Chuanyun, Liangdao1, Liangdao2, Qihe, Suogang, Tanshishan, and Xitoucun), West Liao River (WLR_BA, WLR_BA_o, WLR_LN, and WLR_MN), Yellow River (Miaozigou_MN, Shimao_LN, Upper_YR_IA, Upper_YR_LN, YR_LBIA, YR_LN, and YR_MN) and USR1. Full information about ancient Japanese individuals is included in Table 1 and Table S1, and information about all other previously published data is summarized in Table S4.

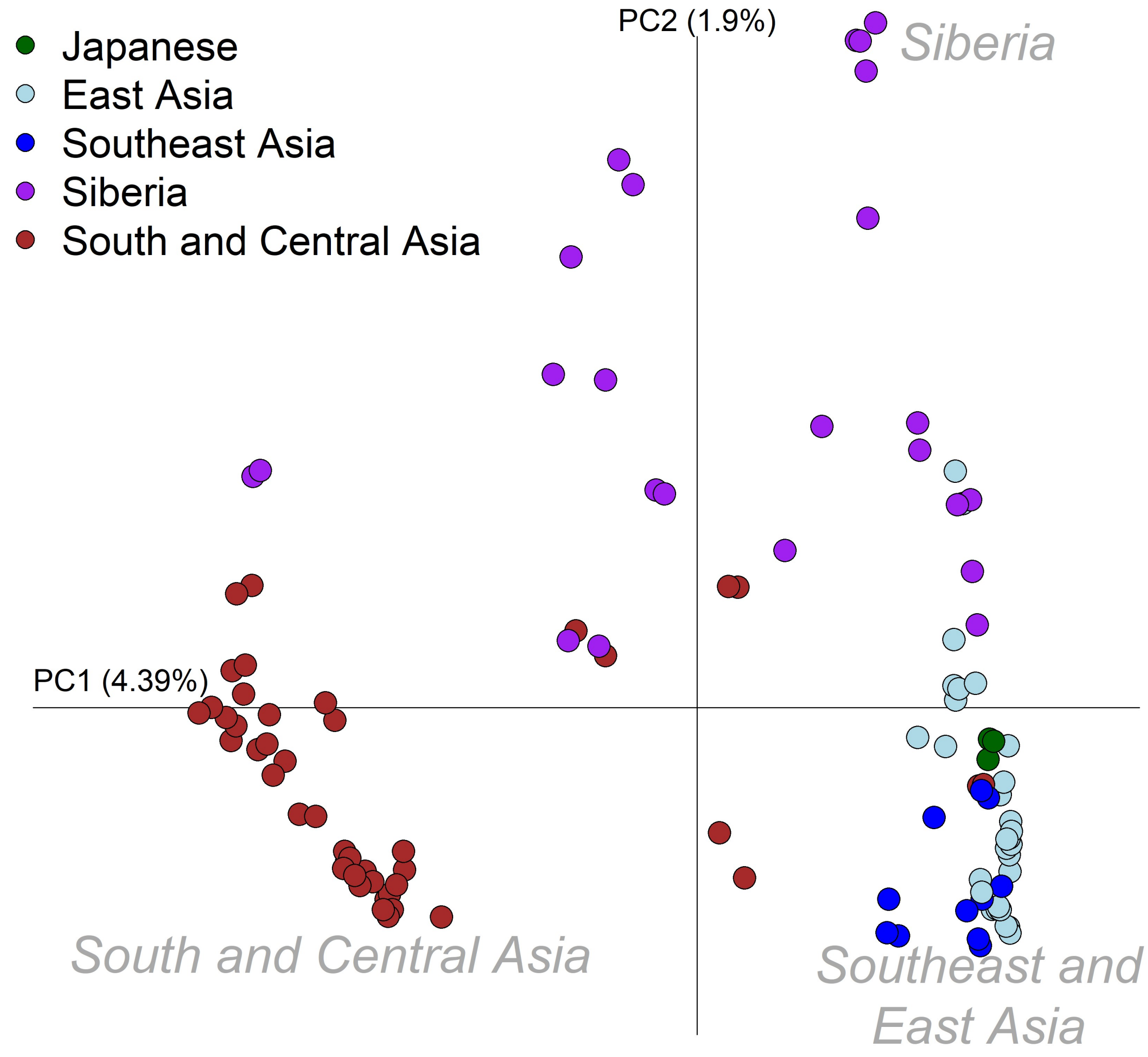

Fig. S4. Principal component analysis of present-day individuals only. Principal components are calculated using 112 present-day East Eurasians in the SGDP panel filtered for transversions and global minor allele frequencies of 1%.

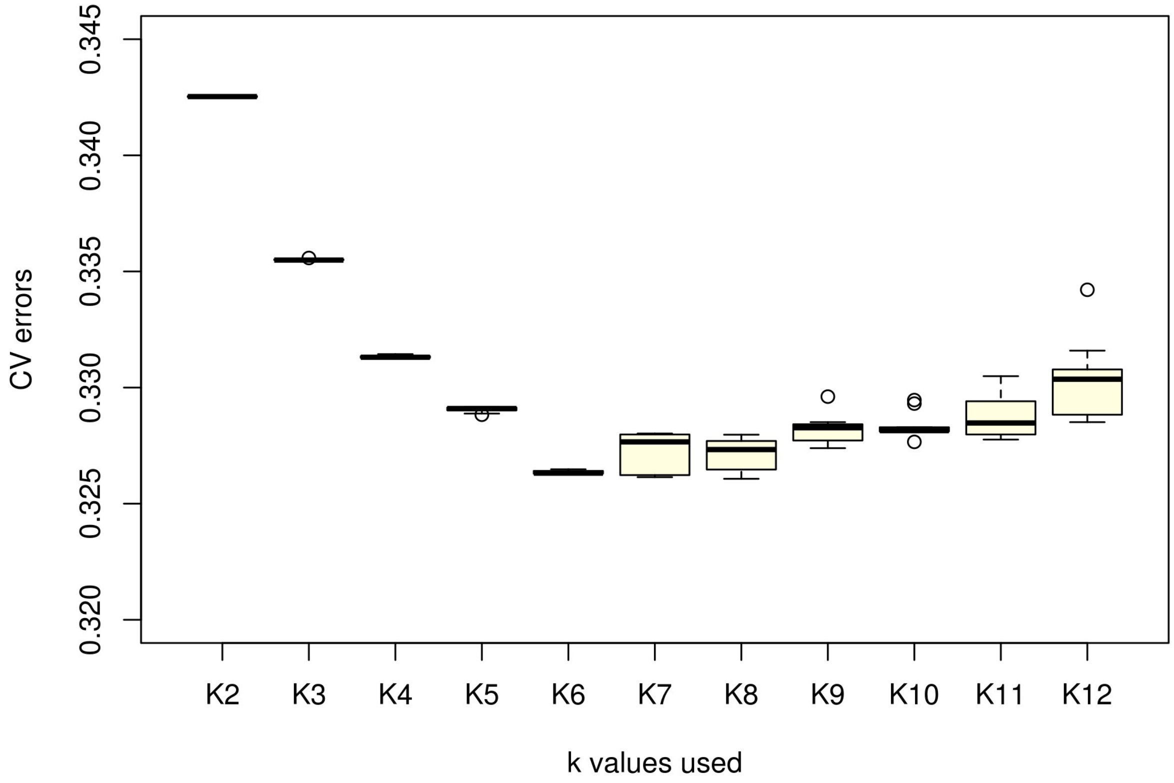

Fig. S5. Cross validation (CV) errors used in ADMIXTURE analysis. Each K component includes ten replicates. The run with the lowest CV is plotted in Fig. S6.

(a)

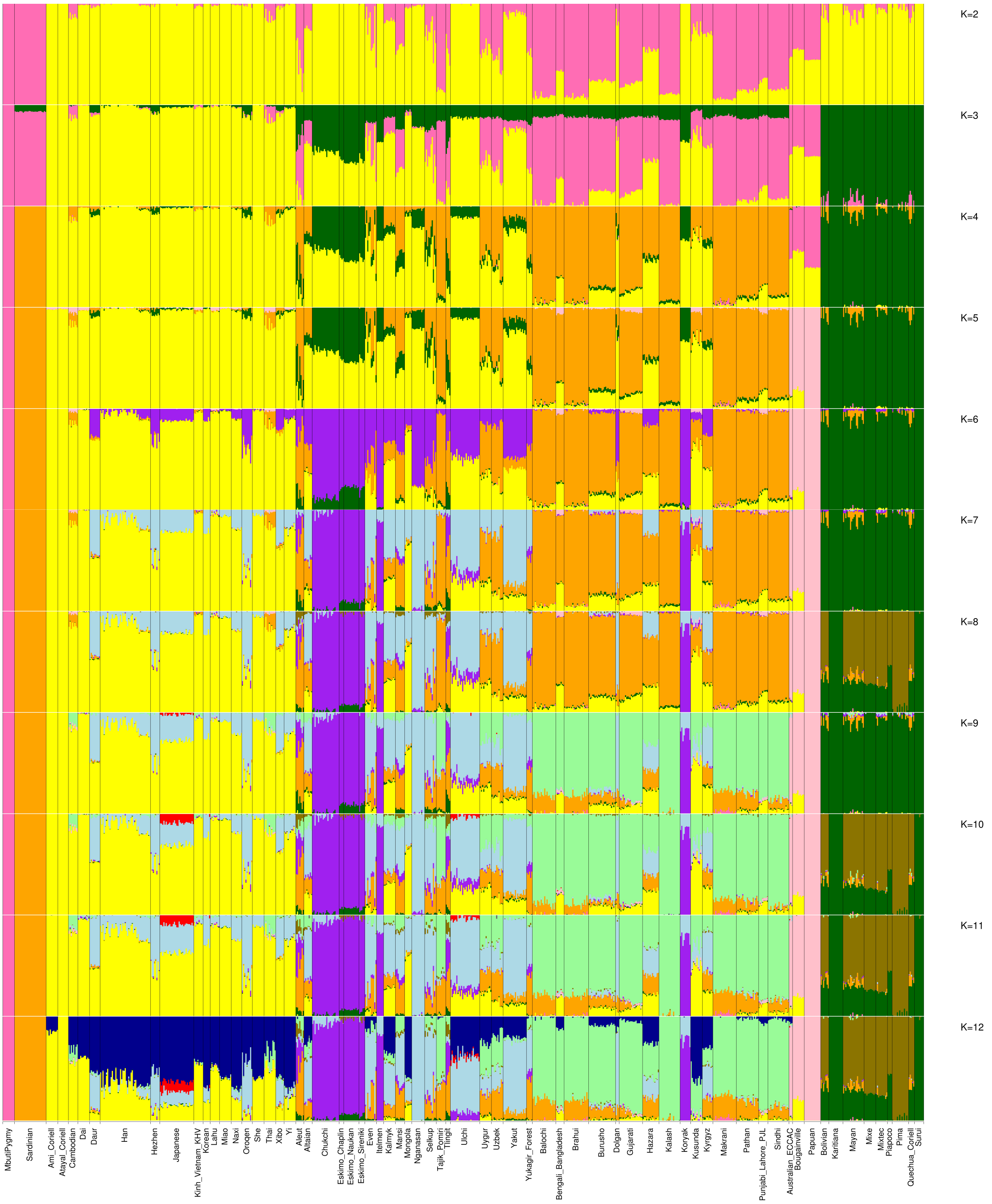
K=2
K=3
K=4
K=5
K=6
K=7
K=8
K=9
K=10
K=11
K=12
MbutiPygmy
Sardinian
Ami_Coriell
Atayal_Coriell
Cambodian
Dai
Daur
Han
Hezhen
Japanese
Kinh_Vietnam_KHV
Korean
Lahu
Miao
Naxi
Oroqen
She
Thai
Xibo
Yi
Aleut
Altaian
Chukchi
Eskimo_Chaplin
Eskimo_Naukan
Eskimo_Sireniki
Even
Itelmen
Kalmyk
Mansi
Mongola
Nganasan
Selkup
Tajik_Pomiri
Tlingit
Ulchi
Uygur
Uzbek
Yakut
Yukagir_Forest
Balochi
Bengali_Bangladesh
Brahui
Burusho
Dolgan
Gujarati
Hazara
Kalash
Koryak
Kusunda
Kyrgyz
Makrani
Pathan
Punjabi_Lahore_PJL
Sindhi
Australian_ECCAC
Bougainville
Papuan
Bolivian
Karitiana
Mayan
Mixe
Mixtec
Piapoco
Pima
Quechua_Coriell
Surui

(b)

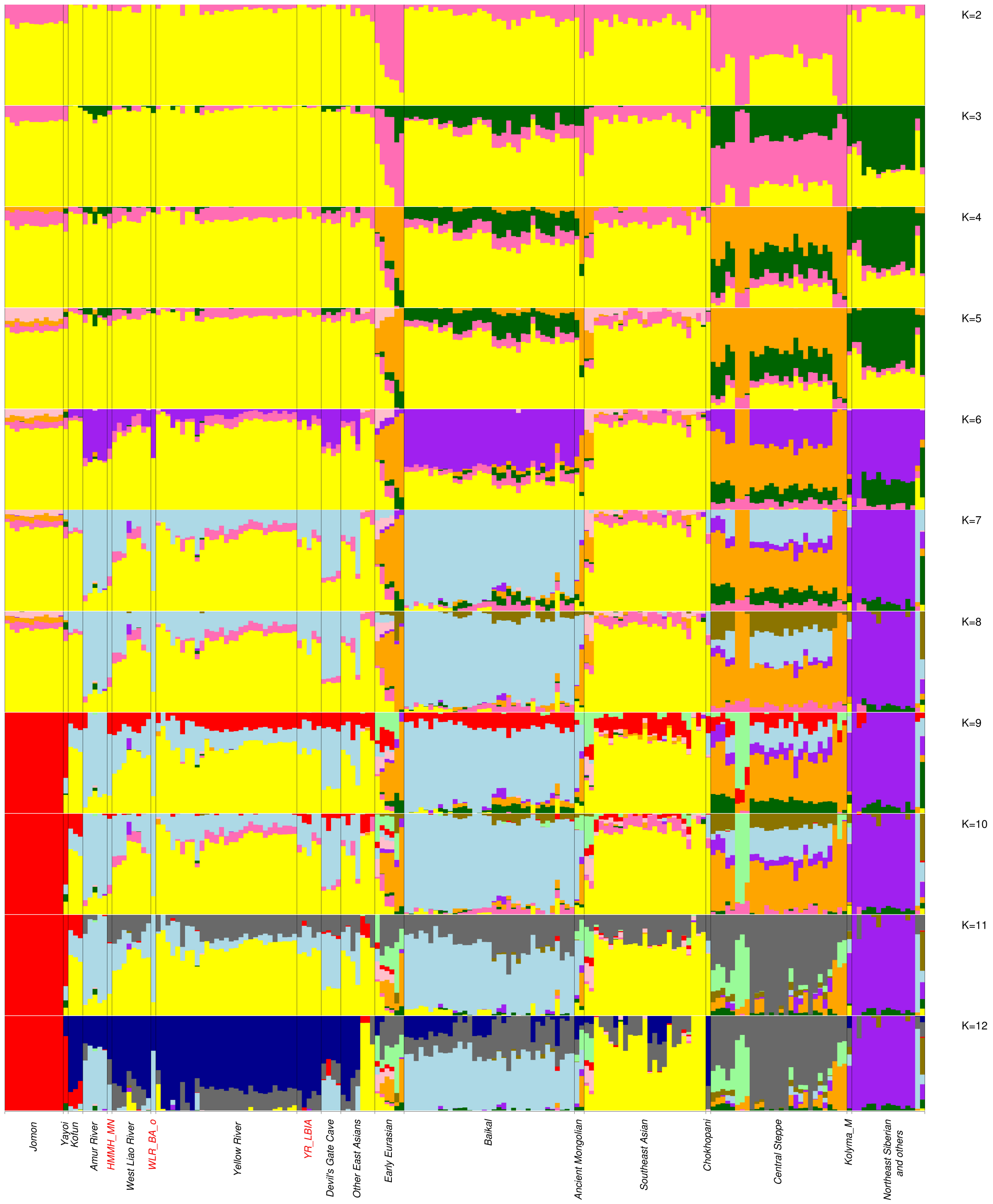

Fig. S6. ADMIXTURE results for 2 to 12 ancestral clusters (K) in (a) modern and (b) ancient individuals. Plotted results for each K represent the run with the lowest cross validation error out of 10 iterations, which have been divided into present-day and ancient populations for clarity. In (a) present-day individuals (n = 786) from the Human Origin Array are separated by region: (from left to right) Africa (represented by Mbuti), Europe (represented by Sardinia), East Asia, Siberia, South and Central Asia, Australasia and Native America. In (b) ancient East Eurasian individuals

with at least 100,000 SNPs in the SGDP database (*n* = 187) are divided into the following broad groups based on culture, age or location of origin: (from left to right) Jomon, Yayoi, Kofun, Amur River (AR_EN, AR_IA, and AR_Xianbei_IA), West Liao River (HMMH_MN, WLR_MN, WLR_LN, WLR_BA, and WLR_BA_o), Yellow River (Upper_YR_LN, Upper_YR_IA, YR_MN, YR_LN, Miaozigou_MN, Shimao_LN, and YR_LBIA), Devil's Gate Cave, Other East Asian (Bianbian, Boshan, Xiaogao, Yumin, Liangdao1, Liangdao2, and Xitoucun), Early Eurasians (Tianyuan, Salkhit, Yana_UP, Mal'ta, and Afontova Gora), Baikal (Shamanka_EN, Shamanka_EBA, Lokomotiv_EN, UstIda_LN, UstIda_EBA, UstBelaya_EBA, UstBelaya_N, UstBelaya_MED, and Kurma_EBA), Ancient Mongolian (ARS008 and ARS026), Southeast Asian (McColl_Group1, McColl_Group2, McColl_Group3, McColl_Group3_1, McColl_Group4, McColl_Group4_1, McColl_Group5, and McColl_Group6), Chokhopani, Central Steppe (Botai, CentralSteppe_EMBA, Namazga_CA, Okunevo_EMBA, SidelkinoEHG_ML, Turkmenistan_IA, and Yamnaya), Kolyma_M, Northeast Siberian and others (Magadan_BA, Uelen_IA, Ekven_IA, Yana_MED, and USR1). In addition, certain ancient groupings that were identified by *qpAdm* as potential sources of admixture for Yayoi (HMMH_MN and WLR_BA_o) and Kofun (YR_LBIA) are highlighted in red. At K=2, the dataset separates into Asian and non-Asian ancestry, denoted by the colors yellow and dark pink respectively. At K=3, a Native American component (darkgreen) emerges that is also observed in lower levels in present-day and ancient Siberians. At K=4, a component representing broad European ancestry (orange) emerges, which is also present in present-day Central Asians, some Early Eurasians and ancient Central Steppe. At K=5, Australasian ancestral component emerges (light pink) that is highest in Papuan. At K=6, a Siberian component (purple) emerges that is seen at its highest level in Koryak and Itelman. At K=7, a component that is highest in Nganasan and strongly observed in ancient Baikal emerges (light blue). At K=8, an ancestral component highest in the Pima population emerges (light brown). At K=9, the Pima component disappears, and two new ones appear - Jomon ancestry becomes distinguishable (red), which is also observed in other ancient and present-day Japanese and at low levels in other samples, and a south and central Asian ancestral component emerges that is highest in Kalash (palegreen). The Pima component reappears in K=10. At K=11, an ancient Central Steppe component is observed (gray). At K=12, a component associated with Yellow River ancestry (deepblue) emerges.

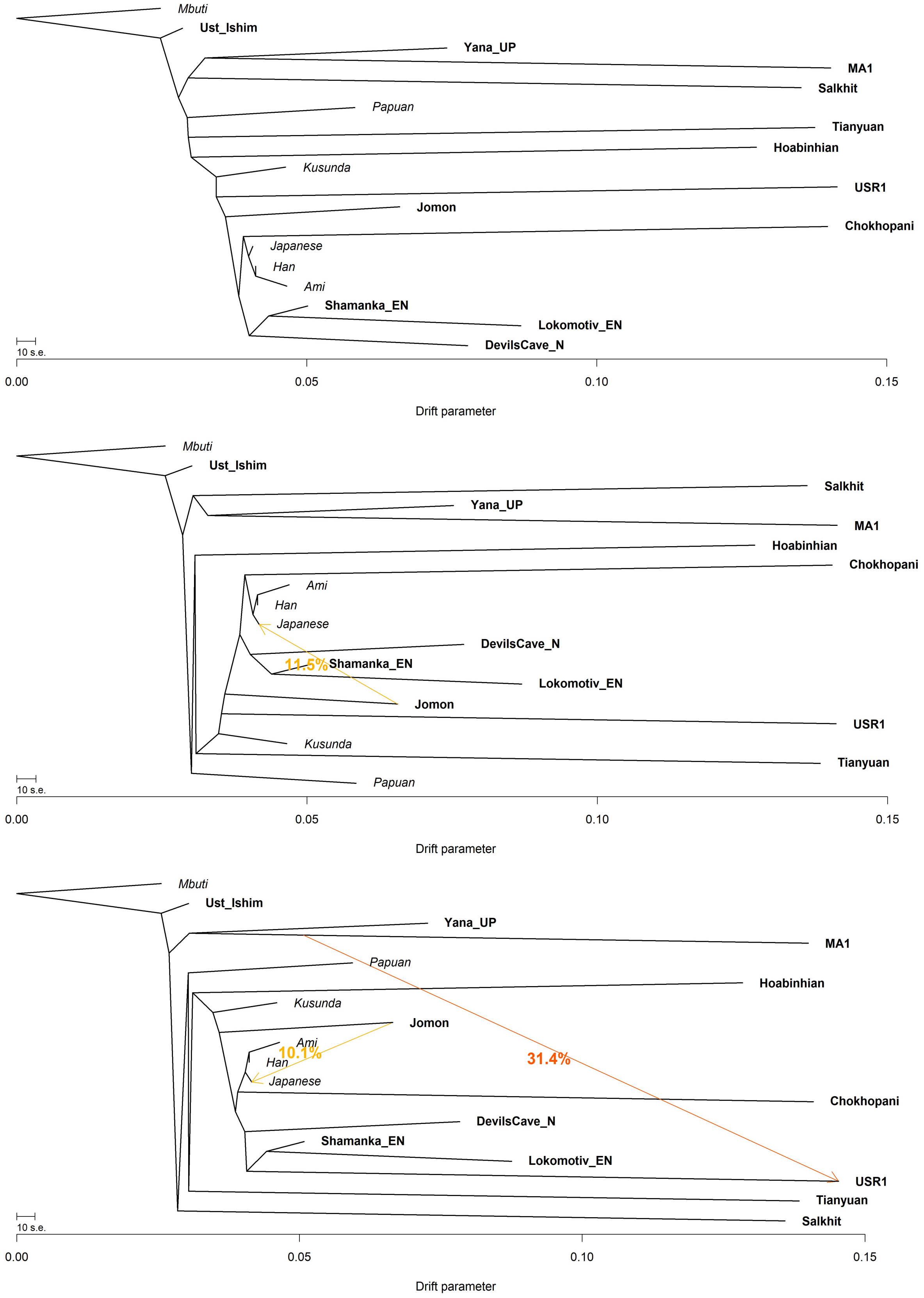
Mbuti
Ust_Ishim
Yana_UP
MA1
Salkhit
Papuan
Tianyuan
Hoabinhian
Kusunda
USR1
Jomon
Chokhopani
Japanese
Han
Ami
Shamanka_EN
Lokomotiv_EN
DevilsCave_N
10 s.e.
0.00
0.05
0.10
0.15
Drift parameter
Mbuti
Ust_Ishim
Salkhit
Yana_UP
MA1
Hoabinhian
Chokhopani
Ami
Han
Japanese
DevilsCave_N
11.5%
Shamanka_EN
Lokomotiv_EN
Jomon
USR1
Kusunda
Tianyuan
Papuan
10 s.e.
0.00
0.05
0.10
0.15
Drift parameter
Mbuti
Ust_Ishim
Yana_UP
MA1
Papuan
Hoabinhian
Kusunda
Jomon
10.1%
Ami
Han
Japanese
31.4%
Chokhopani
DevilsCave_N
Shamanka_EN
Lokomotiv_EN
USR1
Tianyuan
Salkhit
10 s.e.
0.00
0.05
0.10
0.15
Drift parameter

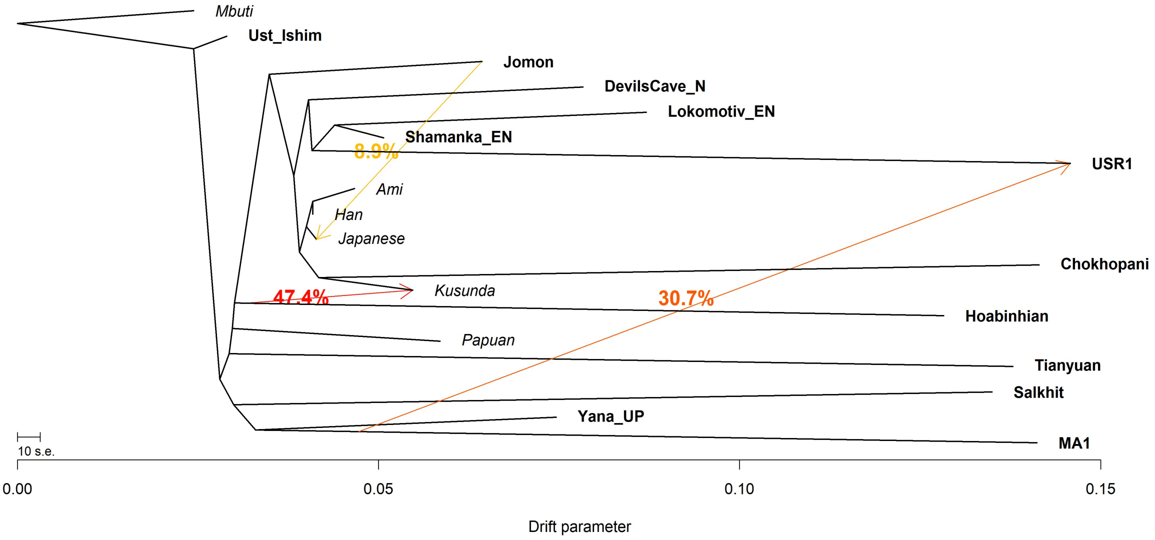
Mbuti
Ust_Ishim
Jomon
DevilsCave_N
Lokomotiv_EN
Shamanka_EN
8.9%
USR1
Ami
Han
Japanese
Chokhopani
Kusunda
47.4%
30.7%
Hoabinhian
Papuan
Tianyuan
Salkhit
Yana_UP
MA1
10 s.e.
0.00
0.05
0.10
0.15
Drift parameter

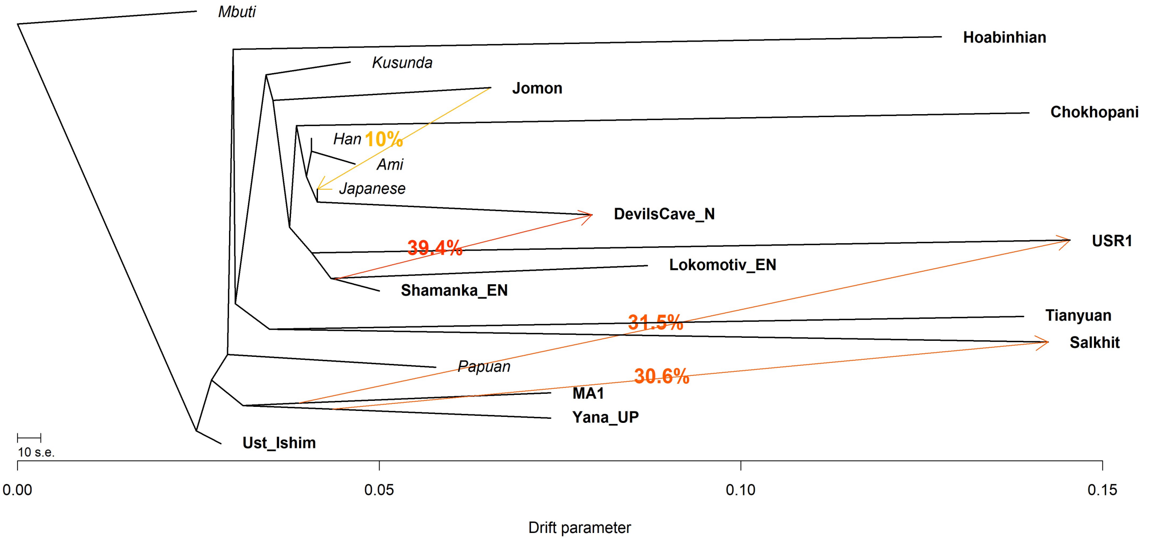
Mbuti
Hoabinhian
Kusunda
Jomon
Chokhopani
Han
10%
Ami
Japanese
DevilsCave_N
USR1
39.4%
Lokomotiv_EN
Shamanka_EN
Tianyuan
31.5%
Salkhit
Papuan
30.6%
MA1
Yana_UP
Ust_Ishim
10 s.e.
0.00
0.05
0.10
0.15
Drift parameter

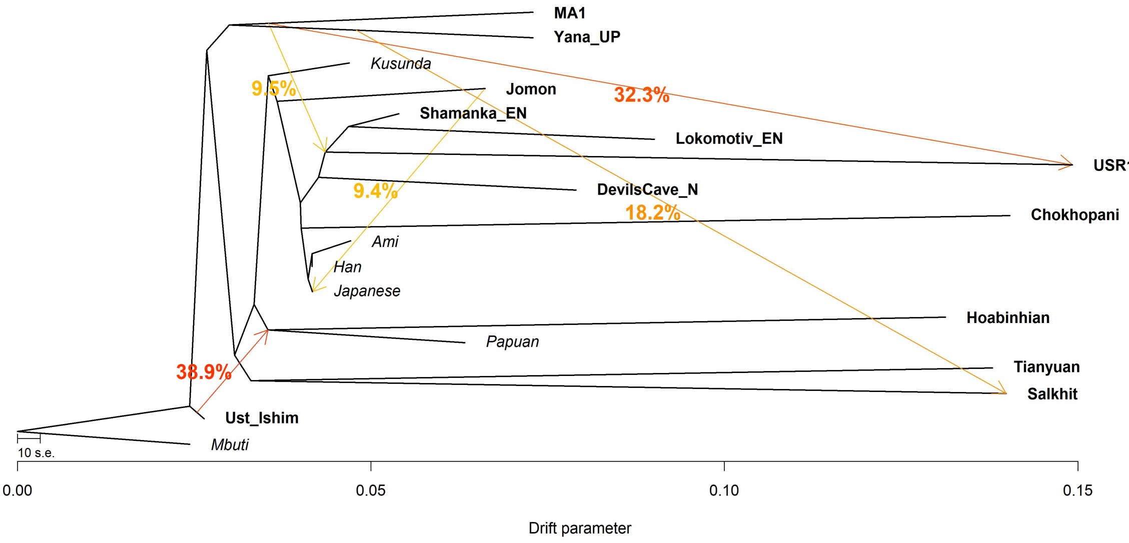
MA1
Yana_UP
Kusunda
9.5%
Jomon
32.3%
Shamanka_EN
Lokomotiv_EN
USR1
9.4%
DevilsCave_N
18.2%
Chokhopani
Ami
Han
Japanese
Hoabinhian
Papuan
38.9%
Tianyuan
Salkhit
Ust_Ishim
Mbuti
10 s.e.
0.00
0.05
0.10
0.15
Drift parameter

Fig. S7. Maximum-likelihood phylogenetic trees reconstructed by *TreeMix*. The trees show phylogenetic relationships among ancient (bold) and present-day (italic) populations under a no-migration model or models with migrations from 1 to 5. Colored arrows represent the migration pathways and signals of admixture among all datasets. The migration weight represents the fraction of ancestry derived from the migration edge.

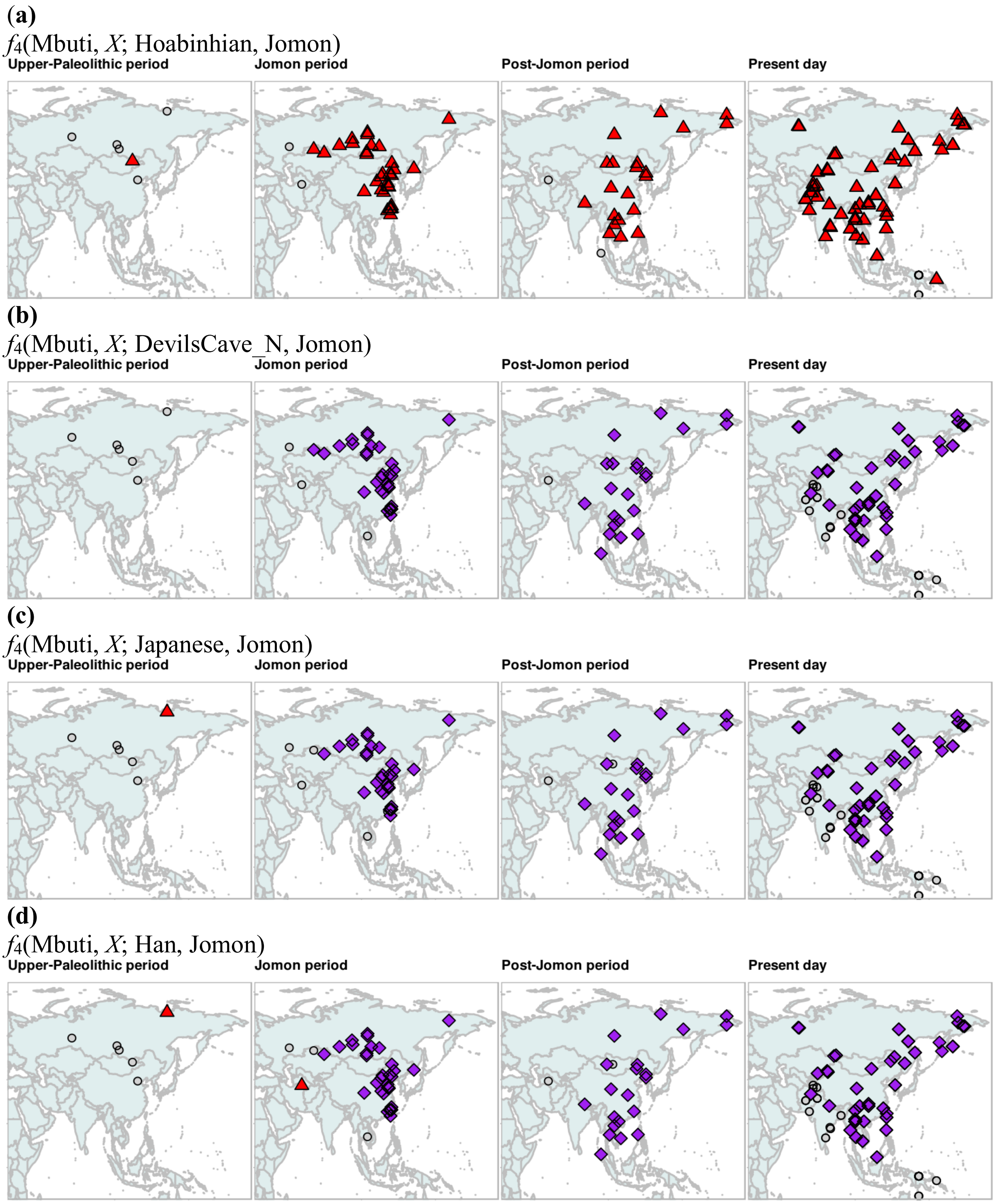
(a)
f4(Mbuti, X; Hoabinhian, Jomon)
Upper-Paleolithic period
Jomon period
Post-Jomon period
Present day
(b)
f4(Mbuti, X; DevilsCave_N, Jomon)
Upper-Paleolithic period
Jomon period
Post-Jomon period
Present day
(c)
f4(Mbuti, X; Japanese, Jomon)
Upper-Paleolithic period
Jomon period
Post-Jomon period
Present day
(d)
f4(Mbuti, X; Han, Jomon)
Upper-Paleolithic period
Jomon period
Post-Jomon period
Present day

(e)

f_4(Mbuti, *X*; Dai, Jomon**)**

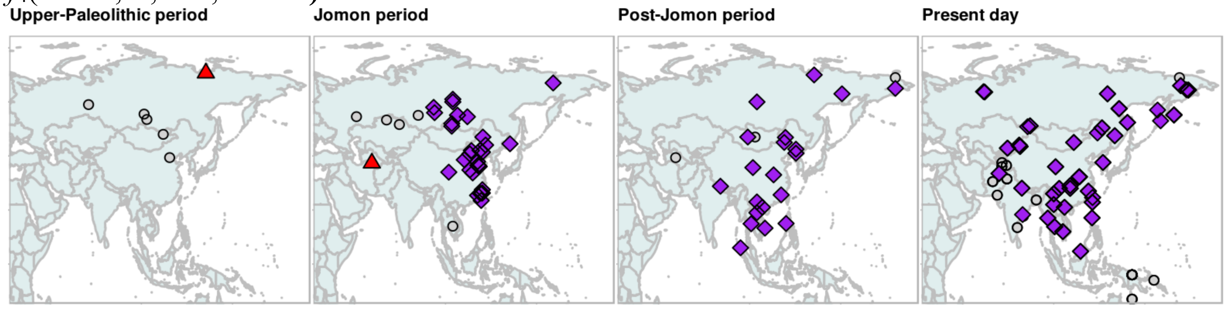

Fig. S8. Geographic and temporal display of f_4(Mbuti, *X*; *Y*, Jomon) results. We compare the different affinities of all ancient and modern East Eurasians to the Jomon with respect to five East Asian populations (*i.e.*, reference populations): (a) Hoabinhian, (b) DevilsCave_N, (c) Japanese, (d) Han, and (e) Dai. Results are divided into four periods based on the prehistory of Japan: the Upper-Paleolithic period (38,000-16,000 years BP), the Jomon period (16,000-3,000 years BP), the post-Jomon period (3,000 years BP-present) and Present day. Populations who are significantly closer to Jomon are represented by a red triangle and those closer to the reference populations are represented by a purple diamond. Symmetrically-related populations are denoted by a gray circle. The Jomon are genetically closer to nearly all populations with respect to the Hoabinhian hunter-gatherers but have less genetic affinity than East Asian hunter gatherers from Devil's Gate Cave and present-day Japanese, Han and Dai; one notable exception however is that the Jomon have extra affinity to the Upper-Paleolithic Yana compared with these present-day reference populations.

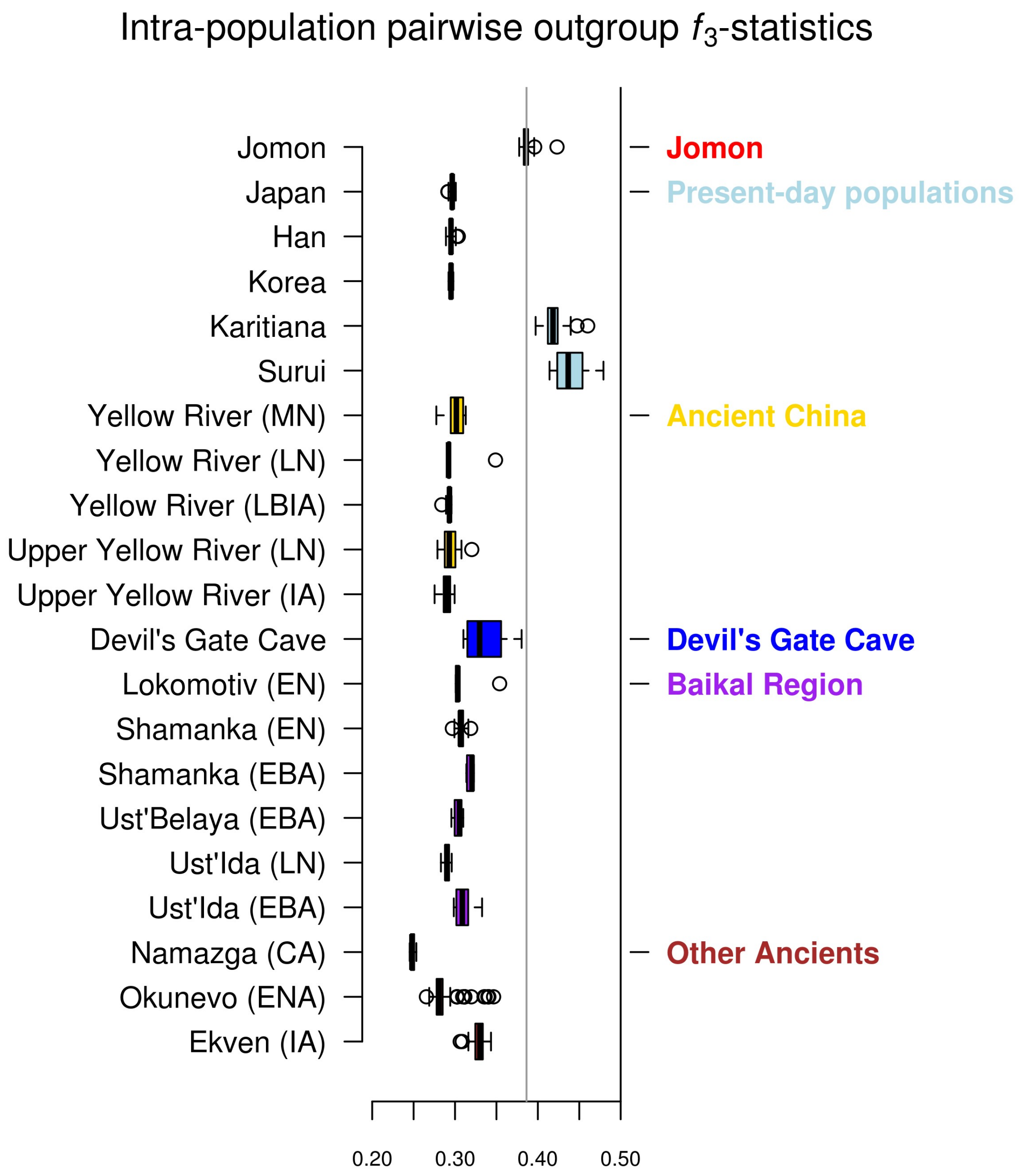

Fig. S9. Distributions of shared genetic drift, measured as outgroup f_3, between all pairs of individuals within a population. Different individuals within the Jomon share more genetic drift than those from ancient or present-day populations tested, except for Karitiana and Surui who are known to be among the most genetically homogenous human populations today.

(a)

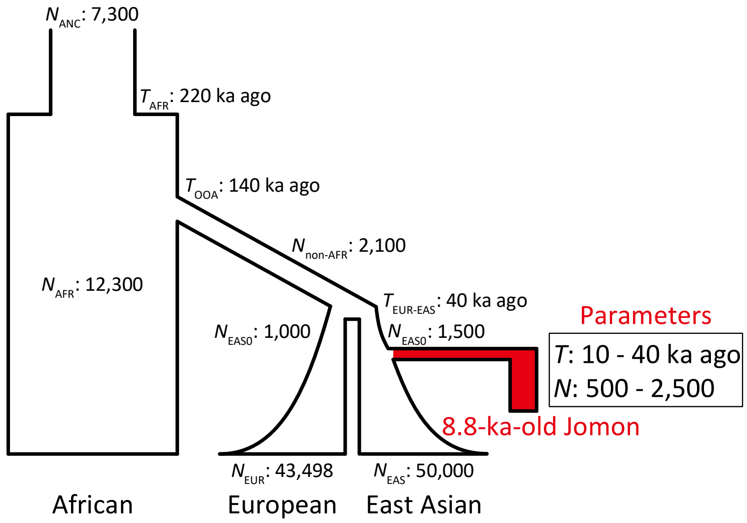
N_{ANC}: 7,300
T_{AFR}: 220 ka ago
T_{OOA}: 140 ka ago
$N_{non\text{-}AFR}$: 2,100
N_{AFR}: 12,300
$T_{EUR\text{-}EAS}$: 40 ka ago
N_{EAS0}: 1,000
N_{EAS0}: 1,500
Parameters
T: 10 - 40 ka ago
N: 500 - 2,500
8.8-ka-old Jomon
N_{EUR}: 43,498
N_{EAS}: 50,000
African
European
East Asian

(b)

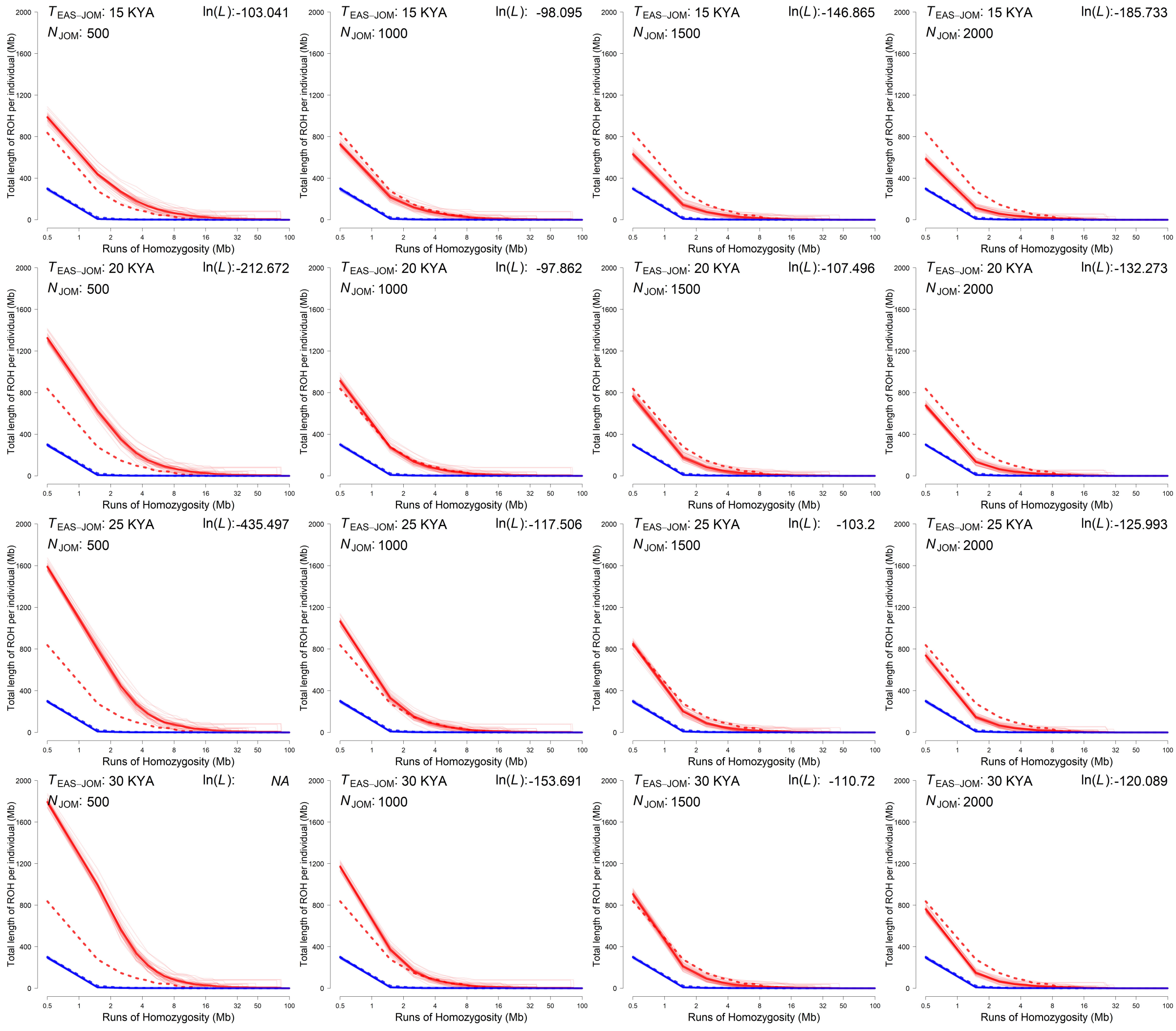
$T_{EAS\text{–}JOM}$: 15 KYA N_{JOM}: 500 ln(L):-103.041
$T_{EAS\text{–}JOM}$: 15 KYA N_{JOM}: 1000 ln(L): -98.095
$T_{EAS\text{–}JOM}$: 15 KYA N_{JOM}: 1500 ln(L):-146.865
$T_{EAS\text{–}JOM}$: 15 KYA N_{JOM}: 2000 ln(L):-185.733
$T_{EAS\text{–}JOM}$: 20 KYA N_{JOM}: 500 ln(L):-212.672
$T_{EAS\text{–}JOM}$: 20 KYA N_{JOM}: 1000 ln(L): -97.862
$T_{EAS\text{–}JOM}$: 20 KYA N_{JOM}: 1500 ln(L):-107.496
$T_{EAS\text{–}JOM}$: 20 KYA N_{JOM}: 2000 ln(L):-132.273
$T_{EAS\text{–}JOM}$: 25 KYA N_{JOM}: 500 ln(L):-435.497
$T_{EAS\text{–}JOM}$: 25 KYA N_{JOM}: 1000 ln(L):-117.506
$T_{EAS\text{–}JOM}$: 25 KYA N_{JOM}: 1500 ln(L): -103.2
$T_{EAS\text{–}JOM}$: 25 KYA N_{JOM}: 2000 ln(L):-125.993
$T_{EAS\text{–}JOM}$: 30 KYA N_{JOM}: 500 ln(L): NA
$T_{EAS\text{–}JOM}$: 30 KYA N_{JOM}: 1000 ln(L):-153.691
$T_{EAS\text{–}JOM}$: 30 KYA N_{JOM}: 1500 ln(L): -110.72
$T_{EAS\text{–}JOM}$: 30 KYA N_{JOM}: 2000 ln(L):-120.089
Total length of ROH per individual (Mb)
Runs of Homozygosity (Mb)

Fig. S10. A demographic model used in the simulation and observed and simulated ROH spectra calculated from the 8.8-ka-old Jomon (JpKa6904) (red) or Han (blue). (a) The Out-of-Africa dispersal model is reconstructed from (*80*), with varying sets of parameters for the population size (N) and divergence time (T) of the Jomon lineage. (b) Solid lines show ROH spectra from 40 simulated data, with a thick line representing a mean of the simulated spectra. Dashed lines show observed ROH in JpKa6904. There are 44 combinations of values of the parameters, population size (N) and split time (T) of the Jomon lineage. Both parameters jointly affect the pattern of ROH. The smaller the population size is, the more short and middle ROH increase. The split time has a further impact on the ROH profiles; the old divergence significantly amplifies a level of ROH carried by the Jomon individual. A log-scaled likelihood is estimated from a similarity between the observed and simulated ROH spectra; *NA* means a likelihood is very close to zero. The model with $N = 1000$ and $T = 20$ ka ago shows the highest likelihood.

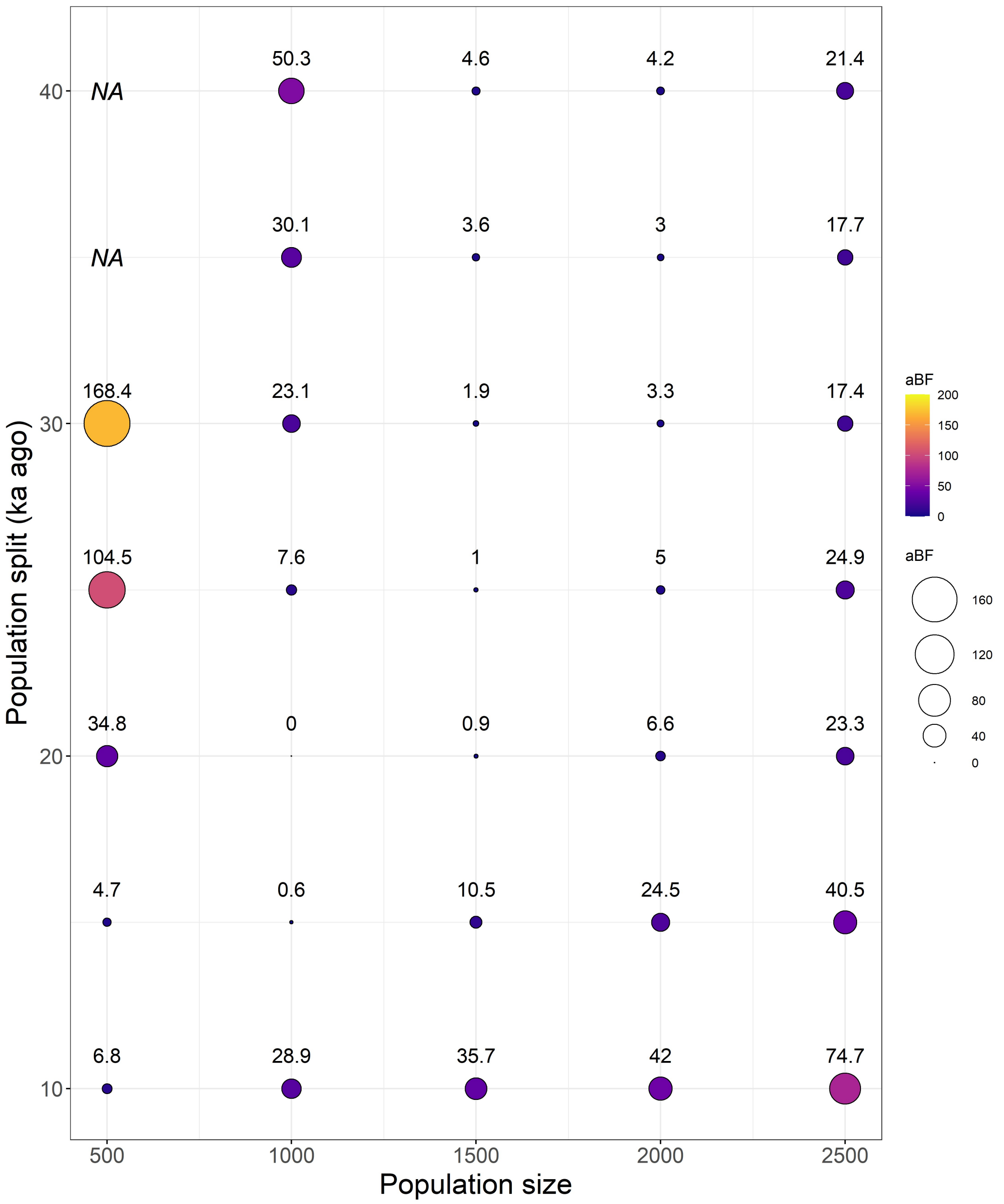

Fig. S11. Modelling on the Jomon demography by ROH. Fitting of the models under different combinations of *N* (*x*-axis) and *T* (*y*-axis) for the 8.8-ka-old Jomon individual. The grid search is based on data from chromosomes 3 to 22. Each point in the balloon plot represents an approximate Bayes factor (aBF) that compares likelihoods between a model with the highest likelihood and each of any other models; the point with aBF = 0 is the model with the highest likelihood (*N* = 1000 and *T* = 20 ka ago). *NA* means that aBF is not measurable for the model due to its zero likelihood.

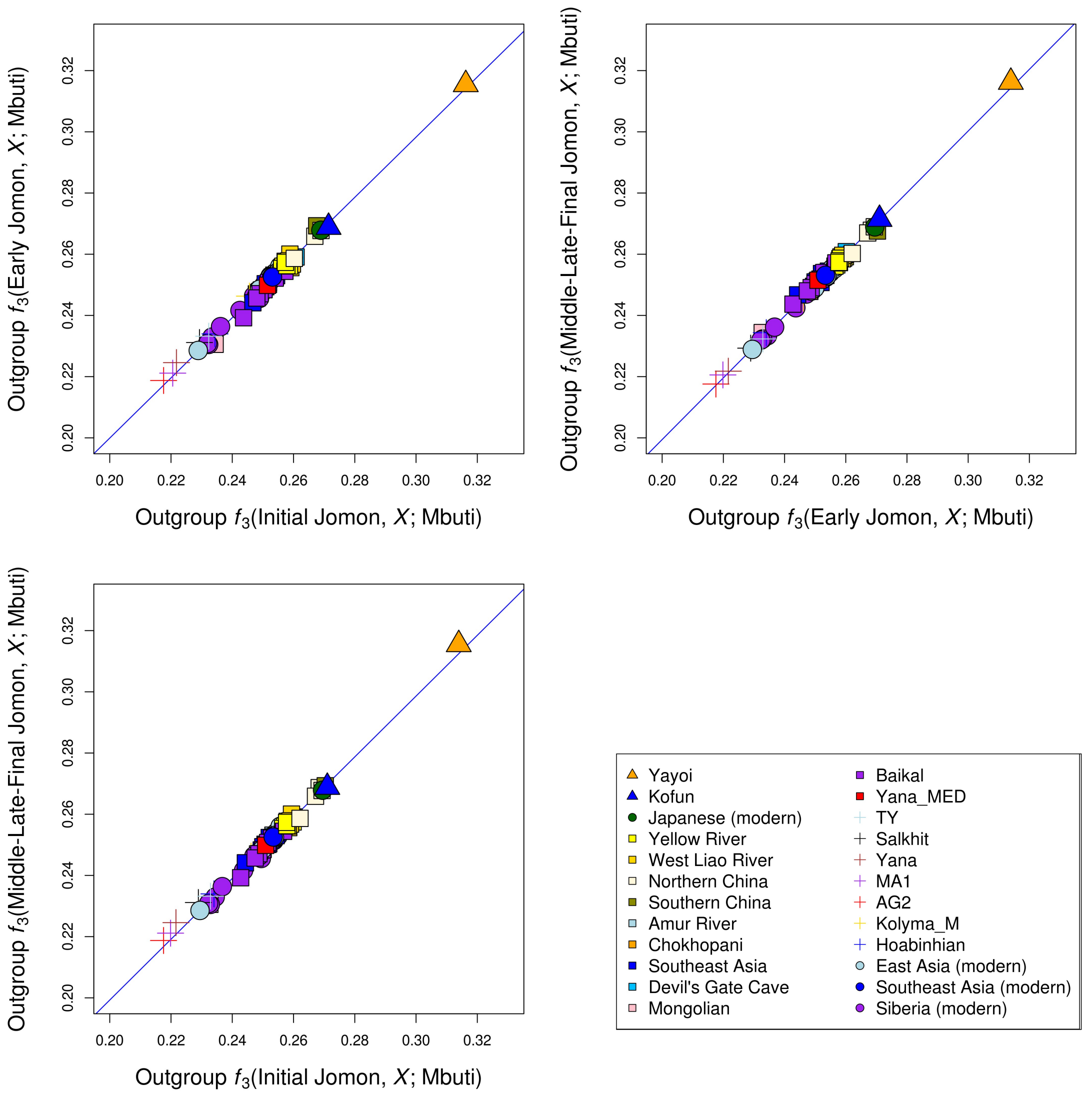

Fig. S12. A comparison of outgroup f_3 results of the Jomon dataset divided into three sub-periods measured using f_3(*Jomon_Sub-Period, X*; Mbuti). The three sub-periods are defined as follows: Initial Jomon (JpKa6906), Early Jomon (JpFu1, JpOd6, JpOd181, JpOd274, and JpOd282), and a merged group for all Middle, Late, and Final Jomon (F5, F23, IK002, JpHi01, JpKo2, and JpKo13). Populations for X plotted are populations whose outgroup f_3-value is calculated using >100,000 SNP sites. The following ancient samples are included: Yayoi, Kofun, Yellow River (Miaozigou_MN, Shimao_LN, Upper_YR_IA, Upper_YR_LN, YR_LBIA, YR_LN, and YR_MN), West Liao River (HMMH_MN,WLR_BA, WLR_BA_o, WLR_LN, and WLR_MN), Northern China (Bianbian, Boshan, Xiaogao, and Yumin), Southern China (Liangdao2), Amur River (AR_EN and AR_Xianbei_IA), Chokhopani, Southeast Asia (McColl Group2, McColl Group3, McColl Group4, McColl Group5, McColl Group6, McColl Group3_1, and McColl Group4_1), Devil's Gate Cave, Mongolian (ARS008 and ARS026), Baikal (Kurma_EBA, Lokomotiv_EN, Shamanka_EBA, Shamanka_EN, UstBelaya_EBA, UstBelaya_MED, UstBelaya_N, Ust_Ida_EBA, and Ust_Ida_LN), Yana_MED, Tianyuan (TY),

Salkhit, Yana, Mal’ta (MA1), Afontova Gora (AG2), Kolyma_M and Hoabinhian (McColl Group1).

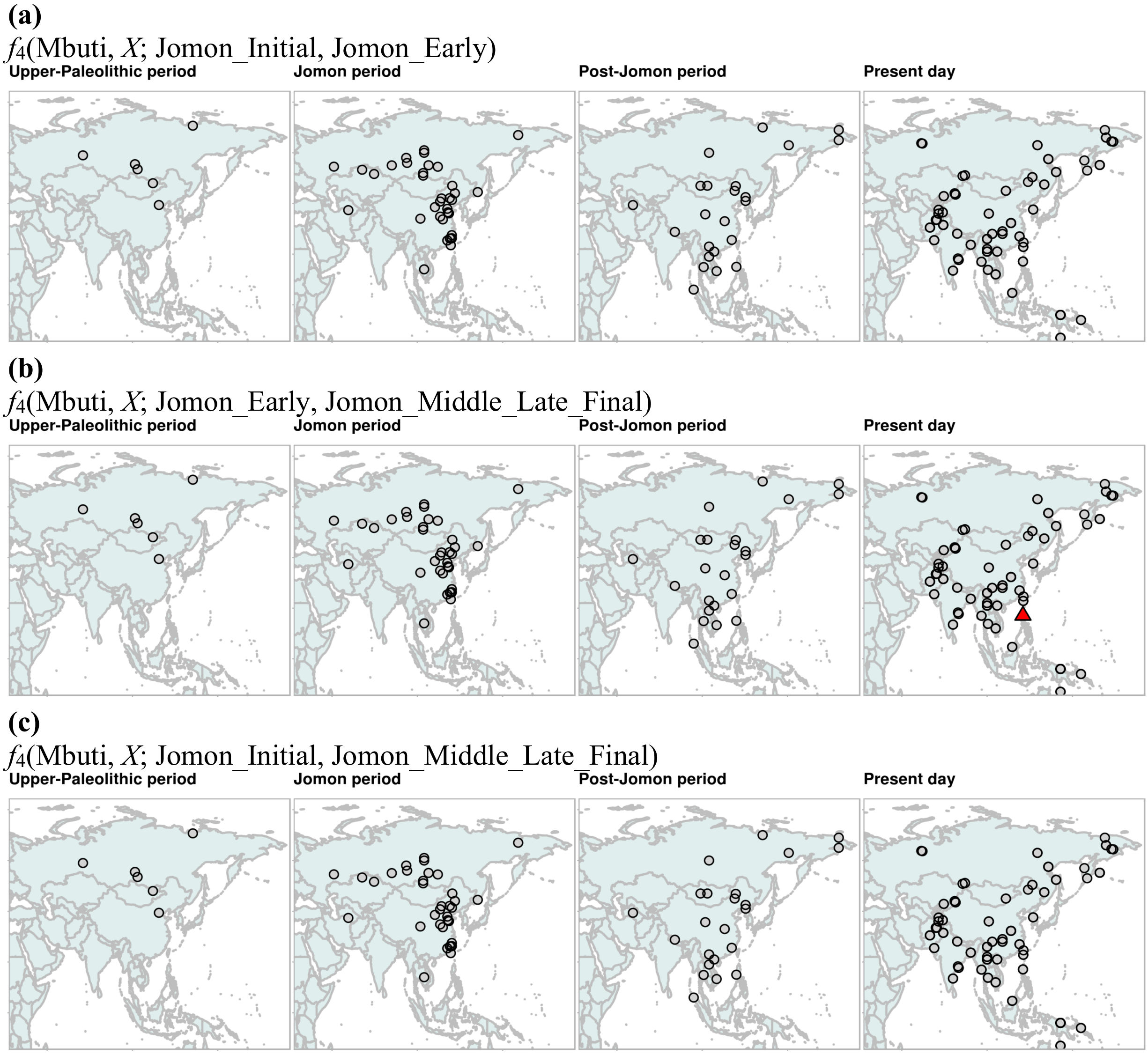

Fig. S13. Geographic and temporal display of differences in affinities between Jomon sub-periods with ancient and modern populations using f_4-statistics. We investigate potential gene flow from continental populations throughout different stages of the Jomon period using f_4(Mbuti, *X*; *sub_Jomon$_i$*, *sub_Jomon$_j$*), where *i* and *j* are any pairs of the three Jomon sub-groups. The three sub-periods are defined as follows: Initial Jomon (JpKa6906), Early Jomon (JpFu1, JpOd6, JpOd181, JpOd274, and JpOd282) and a merged group for all Middle, Late and Final Jomon (F5, F23, IK002, JpHi01, JpKo2, and JpKo13). Populations genetically closer to the later sub-period when compared to the earlier one with Z > 3.0 are designated by red triangles and those symmetrically related to both are designated by gray circles. The populations tested are split into four different periods, depending on their ages: Upper-Paleolithic (>16,000 years BP), Jomon (from 16,000 to 3,000 years BP), Post-Jomon (from 3,000 years BP to the present), and Present-day. There are almost no significant results in this analysis, suggesting that the Jomon were genetically isolated from the rest of the continent from the Initial to Final Jomon period. The Igorot population from the Philippines were found to be significantly closer to the Middle-Late-Final

Jomon subperiod when compared to Early Jomon; however this signal is not replicated with the comparison of Middle-Late-Final Jomon to Initial Jomon.

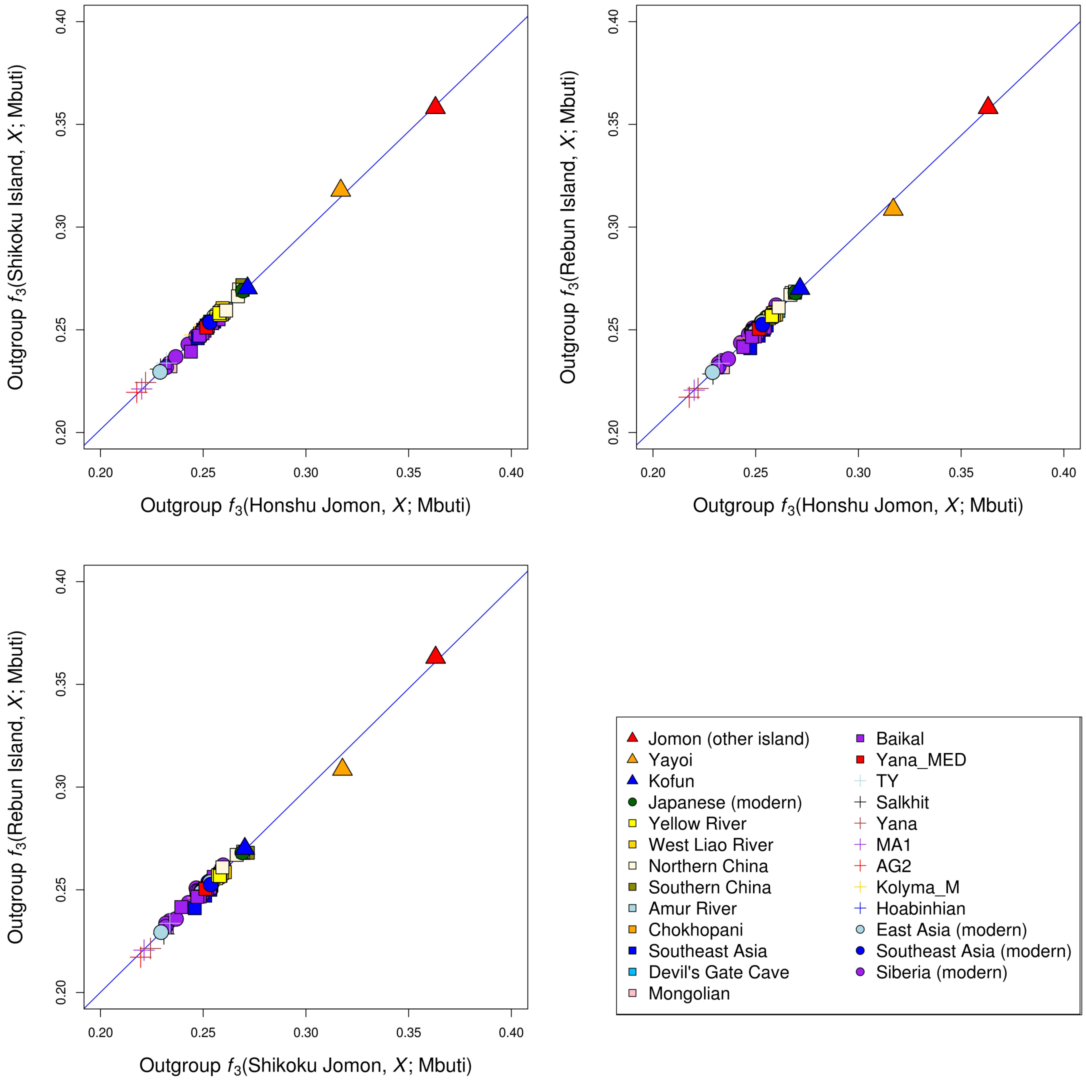

Fig. S14. A comparison of outgroup f_3 results for the Jomon dataset grouped by the island of origin measured using f_3(*Jomon_Sub-Group*, *X*; Mbuti). Jomon are separated into the three islands in the Japanese archipelago from which we have sample data: Honshu (JpFu1, JpOd6, JpOd274, JpOd282, JpOd181, IK002, JpKo2, and JpKo13), Shikoku (JpKa6904 and JpHi01) and Rebun Island (F5 and F23). Populations for *X* plotted are populations whose outgroup f_3-value is calculated using >100,000 SNP sites. The following ancients are included: the Jomon from the island not included in the comparison, Yayoi, Kofun, Yellow River (Miaozigou_MN, Shimao_LN, Upper_YR_IA, Upper_YR_LN, YR_LBIA, YR_LN, and YR_MN), West Liao River (HMMH_MN,WLR_BA, WLR_BA_o, WLR_LN, and WLR_MN), Northern China (Bianbian, Boshan, Xiaogao, Xiaojingshan, and Yumin), Southern China (Liangdao2), Amur River (AR_EN, AR_IA, and AR_Xianbei_IA), Chokhopani, Southeast Asia (McColl Group2, McColl Group3, McColl Group4, McColl Group5, McColl Group6, McColl Group3_1, and McColl Group4_1), Devil's Gate Cave, Mongolian (ARS008 and ARS026), Baikal (Kurma_EBA, Lokomotiv_EN, Shamanka_EBA, Shamanka_EN, UstBelaya_EBA, UstBelaya_MED, UstBelaya_N, Ust_Ida_EBA, and Ust_Ida_LN), Yana_MED, Tianyuan (TY), Salkhit, Yana, Mal'ta (MA1),

Afontova Gora (AG2), Kolyma_M, and Hoabinhian (McColl Group1). The results show a remarkable level of consistency in results between each pair of islands; however there appears to be a slightly greater affinity for Yayoi samples (from northern Kyushu) to Honshu and Shikoku when compared to the geographically distant Rebun Island, which is consistent with Fig. 4c.

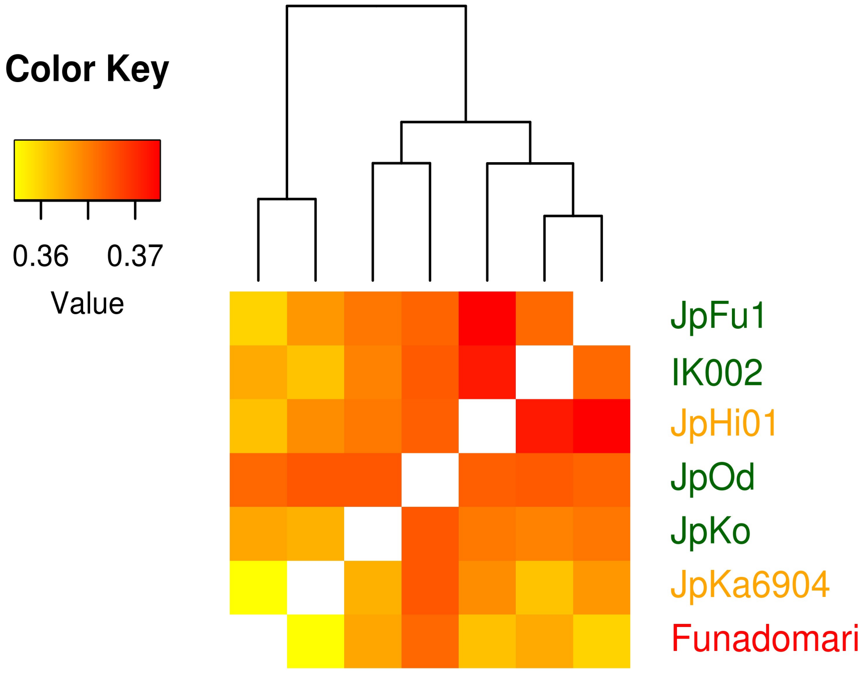

Fig. S15. A heatmap of pairwise outgroup *f*₃ comparisons between Jomon individuals grouped by archaeological site. Shared genetic drift is measured using outgroup f_3(any pair of archaeological sites; Mbuti), with multiple individuals originating from the same site grouped together: JpOd (JpOd6, JpOd181, JpOd274, and JpOd282), JpKo (JpKo2 and JoKo3) and Funadomari (F5 and F23). Site labels are colored according to the different islands on which they are located – Honshu samples are in green, Shikoku in yellow, and Rebun Island in red. There appears to be a slightly increased affinity within samples from Honshu, and the site representing the oldest individual (JpKa6904) and the most northerly site (Funadomari) separate from the others.

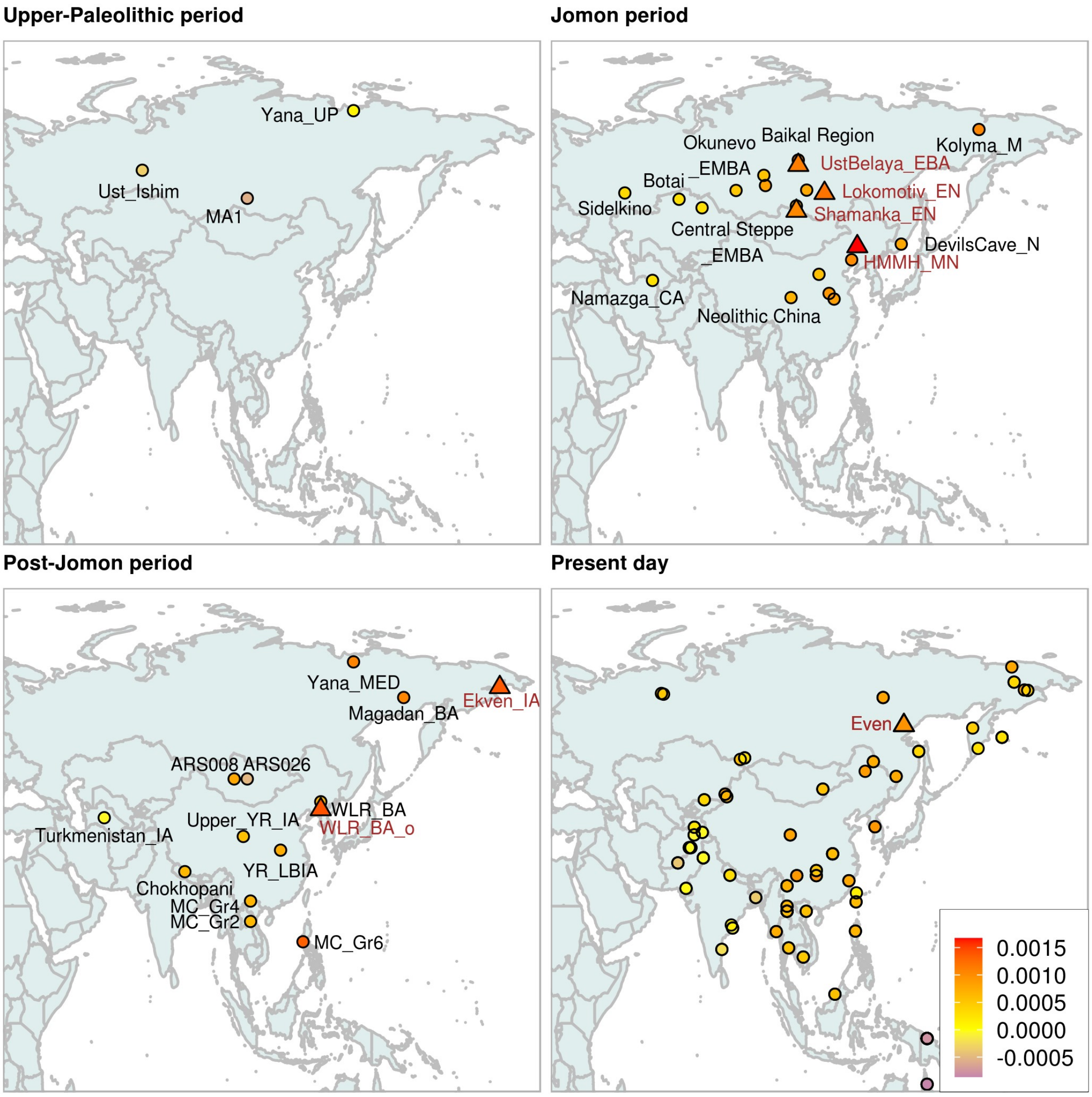

Fig. S16. Geographic and temporal display of *f*4(Mbuti, *X*; Jomon, Yayoi) results. We compare the different affinities of all ancient and modern East Eurasians to the Jomon and the Yayoi. Results are divided into four periods based on the prehistory of Japan: the Upper-Paleolithic period (38,000-16,000 years BP), the Jomon period (16,000-3,000 years BP), the post-Jomon period (3,000 years BP-present) and Present-day. Populations that are significantly closer to Yayoi are marked by a triangle and a brown label and symmetrically-related populations are marked by a circle and a black label. The color within the shapes represents *f*4-values, and only results based on >100,000 SNPs are plotted.

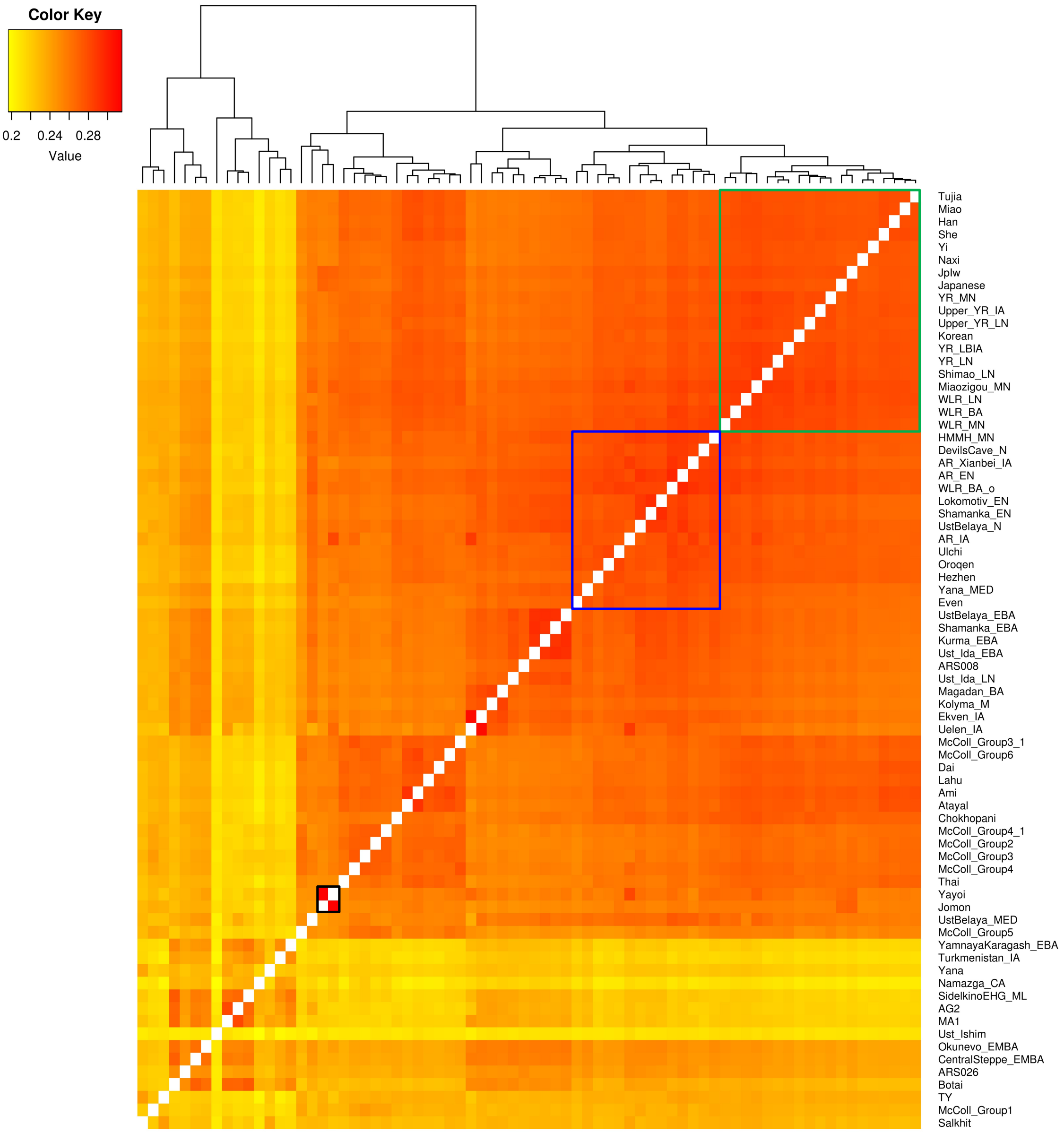

Fig. S17. A heatmap summary of outgroup f_3-statistics for all pairs of ancient and modern East Asians. Outgroup f_3-statistics are based on the form Outgroup f_3(*Pop1*, *Pop2*; Mbuti). We highlight three clusters: 1) the green square shows the high affinity of ancient Yellow River individuals and modern East Asians, 2) the blue square shows ancient and present-day individuals mainly from the Amur River basin and Baikal and 3) the black square shows the high affinity between the Jomon and Yayoi.

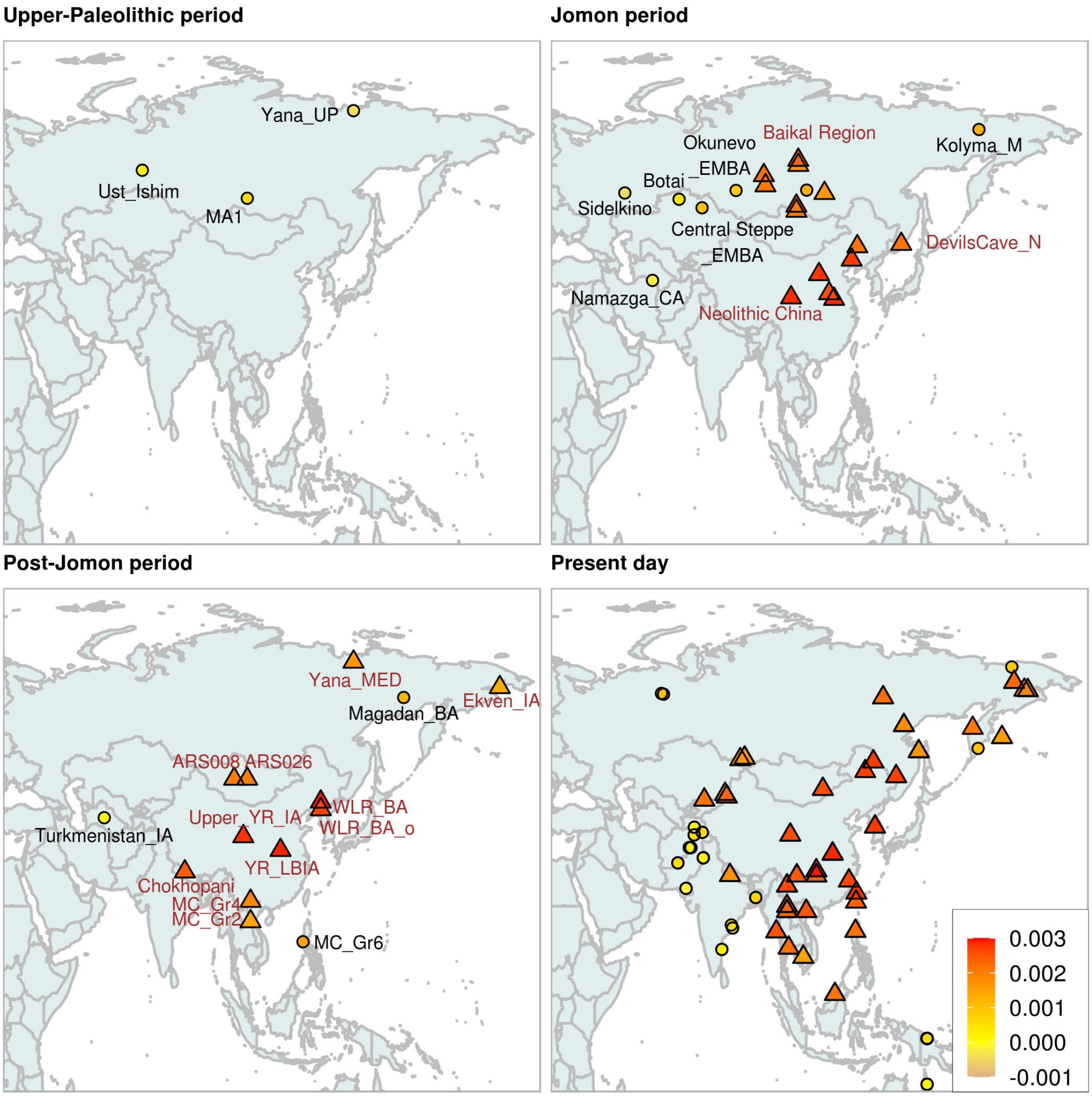

Fig. S18. Geographic and temporal display of f_4(Mbuti, *X*; Yayoi, Kofun) results. We compare the different affinities of all ancient and modern East Eurasians to the Yayoi and the Kofun. Results are divided into four periods based on the prehistory of Japan: the Upper-Paleolithic period (38,000-16,000 years BP), the Jomon period (16,000-3,000 years BP), the post-Jomon period (3,000 years BP-present) and Present-day. Populations that are significantly closer to the Kofun are marked by a triangle and a brown label and symmetrically-related populations are marked by a circle and a black label. The color within the shapes represents f_4-values, and only results based on >100,000 SNPs are plotted.

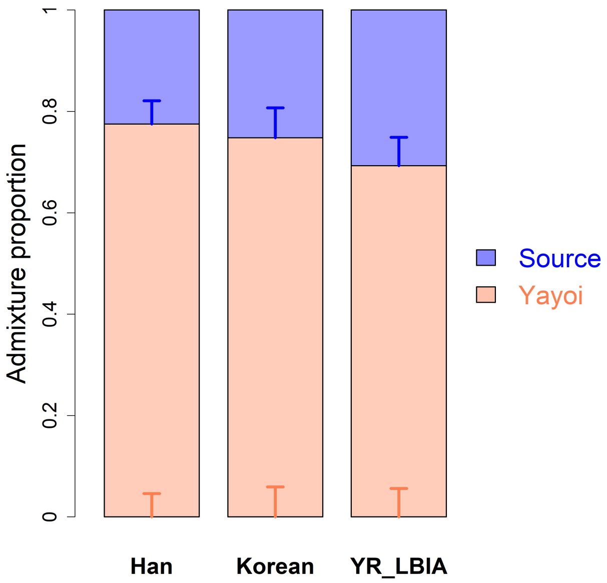

Fig. S19. Genetic ancestry of the Kofun modelled with two-way admixture. Samples from the Kofun period were modelled as two-way admixture between Yayoi and other source represented by Han, Korean, or YR_LBIA. Vertical bars represent ± 1 standard error estimated by *qpAdm*. The values of admixture proportions are shown in Table S9.

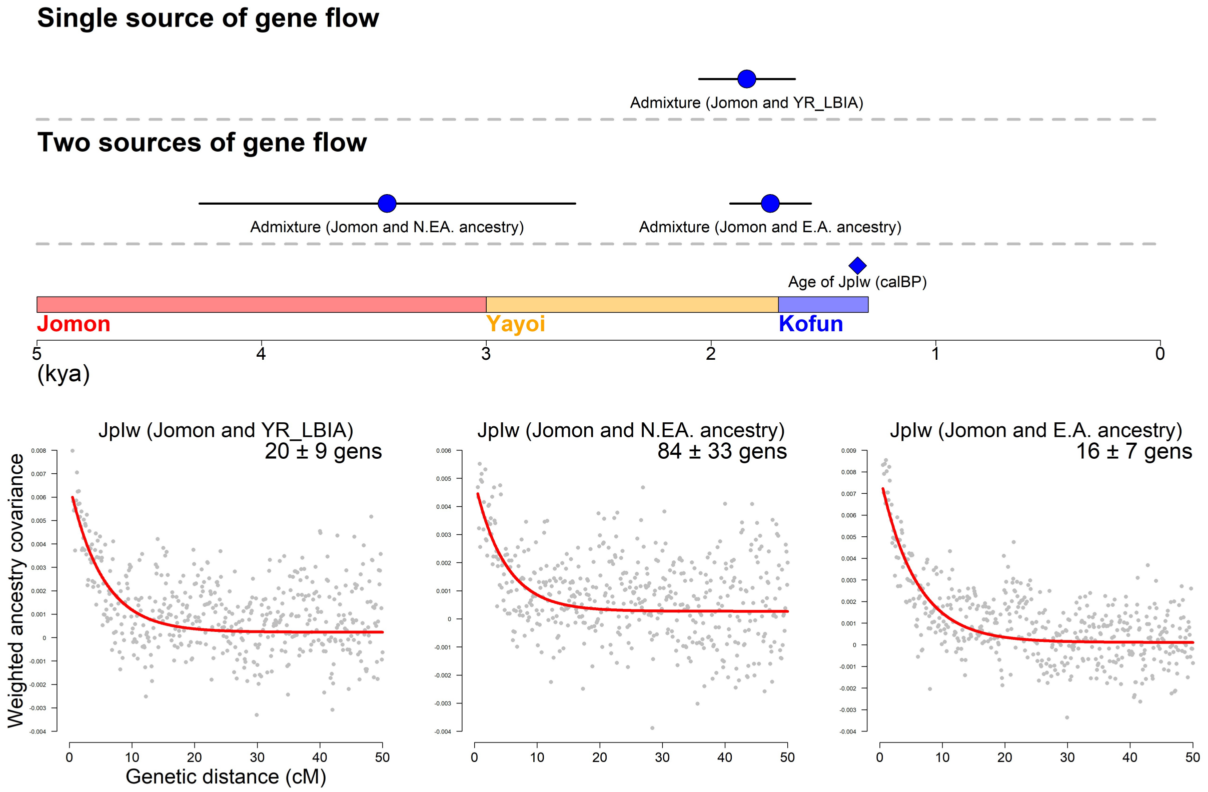

Fig. S20. Dating admixture in the Kofun individuals by the DATES program. Top: Estimated dates of admixture events (circles) and a mean calibrated age across three Kofun individuals (diamond) are plotted. Dating for two scenarios are presented: one in which the Kofun are derived from a single admixture event between Jomon and YR_LBIA, and another allowing for two separate events between the Jomon and Northeast Asian (N.EA.), and between the Jomon and East Asian (E.A.) ancestry. The estimates are converted to a number of years before present by adding the values to the mean age of samples, with an assumption of 25 years per generation. N.EA. and E.A. ancestry is represented by the West Liao River population (WLR_BA_o and HMMH_MN) or CHB in 1000 Genome Phase 3 respectively. The horizontal bars associated with the admixture dates show standard errors estimated by a weighted block jackknife method. **Bottom:** Ancestry covariances in the Kofun individuals. The plots show the exponential decay of weighted ancestry covariance (y-axis) with genetic distance (x-axis), in which a decay rate depends on the time since admixture. Fitting starts at a genetic distance of 0.45 centi Morgan (cM).

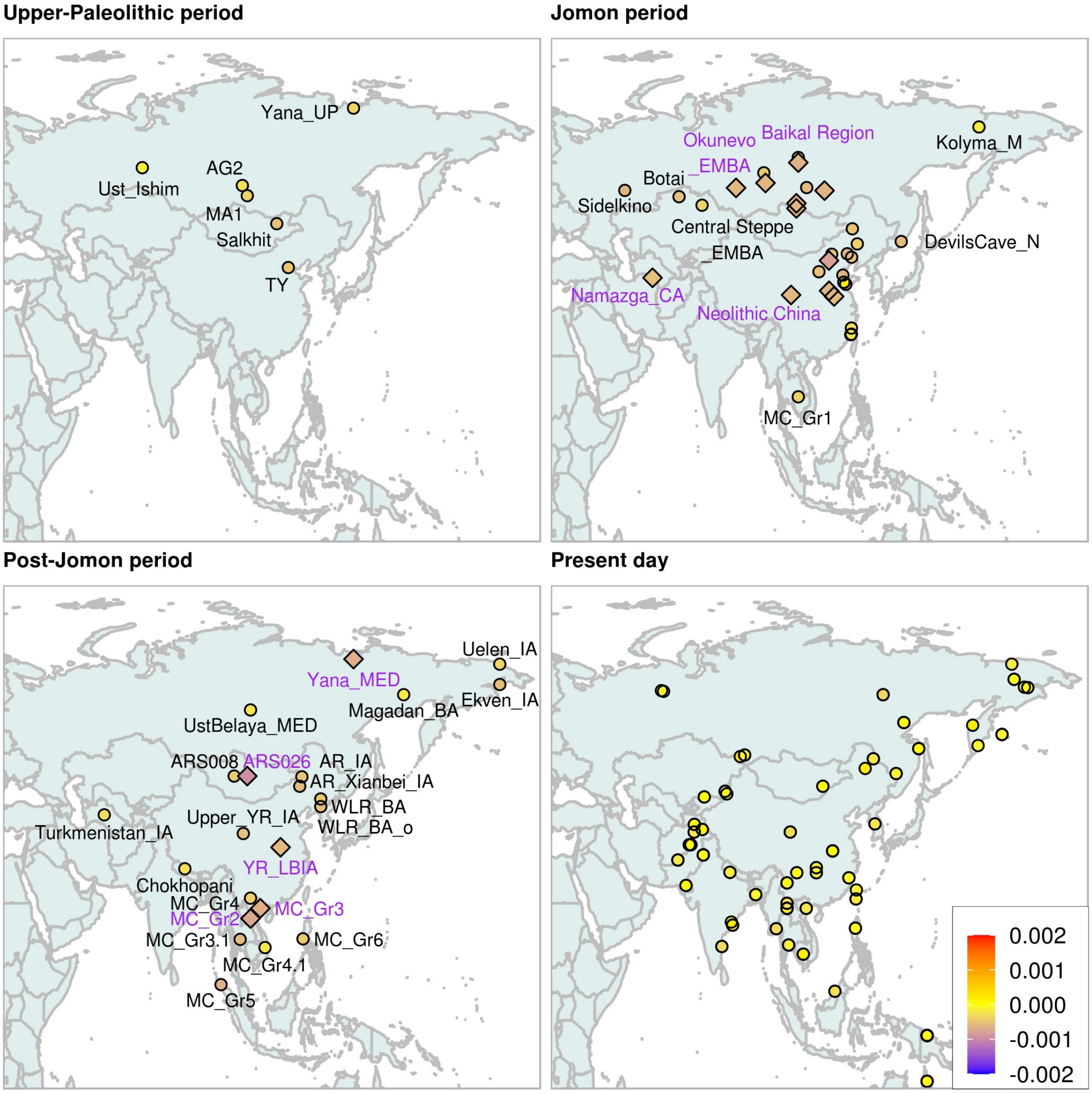

Fig. S21. Geographic and temporal display of f_4(Mbuti, *X*; Kofun, Japanese) results. We compare the different affinities of all ancient and modern East Eurasians to the Kofun and present-day Japanese. Results are divided into four periods based on the prehistory of Japan: the Upper-Paleolithic period (38,000-16,000 years BP), the Jomon period (16,000-3,000 years BP), the post-Jomon period (3,000 years BP-present) and Present-day. Populations that are significantly closer to the Japanese are marked by a diamond and a purple label and symmetrically-related populations are marked by a circle and a black label. The color within the shapes represents f_4-values, and only results based on >100,000 SNPs are plotted.

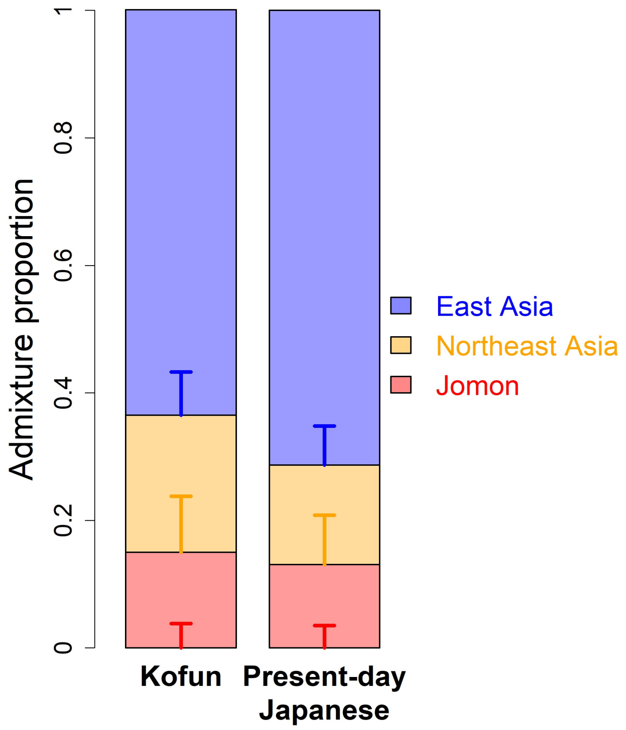

Fig. S22. Genetic ancestry of the Kofun and present-day Japanese population modelled as three-way admixture. The three-way admixture includes Jomon, Northeast Asian (represented by WLR_BA_o and HMMH_MN), and East Asian ancestry (represented by Han). Vertical bars represent ± 1 standard error estimated by *qpAdm*. The values of admixture proportions for the Kofun and Japanese are shown in Tables S10 and S17.

Table S1. Additional information on ancient Japanese samples.

Associated Culture	Sample ID	Radiocarbon dating				#Reads (BAM)	#SNPs (SGDP)	#SNPs (HOA)	Latitude	Longitude
		Lab.Code[a] (Layer or Pit No.)	Age (BC or AD) Top: uncalibrated Bottom: calibrated							
			Lower	Higher	Median					
Jomon	JpKa6904[b]	MTC-13800 (Layer 4)	5,960	6,056	6,008	441,555,803	3,672,440	593,322	33.61340	132.97460
			6,696	7,041	6,869					
	JpOd274	PLD-41178 (Layer 3)	3,425	3,477	3,451	78,075,659	2,770,870	460,505	36.72122	137.16677
			4,169	4,339	4,254					
	JpOd6	PLD-41176 (Layer 3)	3,282	3,336	3,309	6,3096,322	2,107,727	344,858	36.72122	137.16677
			3,984	4,229	4,107					
	JpOd181	PLD-41177 (Layer 3)	3,122	3,174	3,148	108,925,378	2,892,871	511,099	36.72122	137.16677
			3,801	3,967	3,884					
	JpOd282	PLD-41179 (Layer 3)	3,080	3,132	3,106	53,803,318	2,040,722	370,729	36.72122	137.16677
			3,787	3,952	3,870					
	JpFu1	PLD-39693 (Pit 1)	2,825	2,871	2,848	67,453,090	2,301,273	380,012	34.59220	133.77320
			3,528	3,640	3,584					
	JpKo2	PLD-39690 (Pit K2)	1,970	2,014	1,992	147,935,560	3,213,950	538,334	35.72088	139.96344
			2,344	2,564	2,454					
	JpKo13	PLD-39691 (Pit K13)	1,636	1,680	1,658	111,027,919	2,914,377	495,272	35.72088	139.96344
			1,897	2,028	1,963					
	JpHi01	TKA-18417 (Burial No. 3)	-	-	-	60,272,828	1,796,203	303,320	32.96353	132.56477
			1,735	1,900	1,818					
Kofun	JpIw32	PLD-39692 (Pit 3)	418	458	438	258,117,426	3,630,288	589,026	36.61895	136.70233
			541	603	572					
	JpIw31	UBA40246 (Pit 3)	468	516	492	74,816,071	2,718,997	447,147	36.61895	136.70233
			573	647	610					
	JpIw33	UBA40248	499	545	522	79,160,173	2,810,991	470,568	36.61895	136.70233

		(Pit 3)	595	655	625					

[a]Abbreviations of Lab Code are as follows: Paleo Labo Co., Ltd, Japan (PLD), Research Center for Nuclear Science and Technology, University of Tokyo, Japan (MTC), Belfast 14CHRONO Centre, Ireland (UBA).
[b]The radiocarbon dating results of JpKa6904 are referred from (*89*).
[c]The radiocarbon dating results of JpHsi01 are referred from the Annual Archaeological Report from Ainan Town 2020, where only calibrated dates were reported.

Table S2. Contamination rate estimates based on mtDNA.

Sample ID	mtDNA Contamination rates	
	All sites	All sites without molecular damage
JpKa6904	1.461	JpKa6904
JpOd274	1.131	JpOd274
JpOd6	1.546	JpOd6
JpOd181	0.909	JpOd181
JpOd282	1.377	JpOd282
JpFu1	2.152	JpFu1
JpKo2	1.436	JpKo2
JpKo13	1.495	JpKo13
JpHi01	1.446	JpHi01
JpIw32	0.405	JpIw32
JpIw31	0.625	JpIw31
JpIw33	0.748	JpIw33

Table S3. Sexing results for newly sequenced data

Sample ID	#Reads mapped to X chromosome	#Reads mapped to Y chromosome	#Total (X + Y)	Rx	Ry	SE	Assigned Sex
JpKa6904	21,685,400	122,597	21,807,997	0.994	0.006	5.02E-10	F
JpFu1	3,265,205	18,886	3,284,091	0.994	0.006	3.41E-09	F
JpKo2	7,234,163	41,638	7,275,801	0.994	0.006	1.53E-09	F
JpKo13	5,435,655	31,311	5,466,966	0.994	0.006	2.04E-09	F
JpHi01	2,907,655	16,525	2,924,180	0.994	0.006	3.77E-09	F
JpOd274	1,933,203	200,709	2,133,912	0.906	0.094	7.83E-08	M
JpOd6	3,060,597	24,441	3,085,038	0.992	0.008	4.99E-09	F
JpOd181	2,593,378	259,235	2,852,613	0.909	0.091	5.68E-08	M
JpOd282	1,275,323	130,481	1,405,804	0.907	0.093	1.17E-07	M
JpIw31	3,670,669	22,888	3,693,557	0.994	0.006	3.27E-09	F
JpIw32	6,595,047	644,474	7,239,521	0.911	0.089	2.20E-08	M
JpIw33	3,939,727	21,514	3,961,241	0.995	0.005	2.67E-09	F

Table S4. A summary of all processed published data included in this analysis.

Population Group	Individual IDs	Median calBP[a]	Removed as 1st degree relatives	#Inds included	Downloaded data format	Data type	References
-	Mal'ta (MA1)	24,157	-	1	BAM	WGS	Raghavan *et al*. (2014); (*86*)
-	Afontova Gora (AG2)	16,913	-	1	BAM	WGS	
-	Chokhopani	2,775	-	1	FASTQ	WGS	Jeong *et al*. (2016); (*91*)
-	Salkhit	34,425	-	1	BAM	Capture	Massilani *et al*. (2020); (*92*)
-	Tianyuan (TY)	40,328	-	1	BAM	Capture	Yang *et al*. (2017); (*85*)
-	USR1	11,500*	-	1	BAM	WGS	Moreno-Mayar *et al*. (2018); (*78*)
Botai	BOT14, BOT15, BOT2016	5,399 - 5,161	-	3	FASTQ	WGS	de Barros Damgaard *et al*. (2018); (*17*)
CentralSteppe_EMBA	EBA1, EBA2	4,200*	-	2	FASTQ	WGS	
Kurma_EBA	DA354, DA358, DA360	4,169 - 4,078*	-	3	FASTQ	WGS	
Lokomotiv_EN	DA340, DA341, DA357, DA359	6,713*	-	4	FASTQ	WGS	
Namazga_CA	DA379, DA380, DA381, DA383	5,263 - 5,173	DA379	3	FASTQ	WGS	
Okunevo_EMBA	RISE515, RISE516, RISE662, RISE664, RISE667, RISE670, RISE671, RISE672, RISE673, RISE674, RISE675, RISE677, RISE680, RISE681, RISE683, RISE684, RISE685, RISE718, RISE719	4,555 - 3,963	RISE516, RISE673	17	FASTQ	WGS	
Shamanka_EBA	DA334, DA335, DA336, DA337, DA338,DA339	3,871 - 3,700*	DA335, DA338	4	FASTQ	WGS	
Shamanka_EN	DA245, DA246,DA247, DA248, DA249, DA250, DA251, DA252, DA253, DA362	7,123 - 6,319*	-	10	FASTQ	WGS	
SidelkinoEHG_ML	Sidelkino	11,259	-	1	FASTQ	WGS	
Turkmenistan_IA	DA382	2,805	-	1	FASTQ	WGS	
UstIda_EBA	DA343, DA353, DA356, DA361	3,854*	-	4	FASTQ	WGS	
UstIda_LN	DA342, DA344, DA345, DA355	4,885*	-	4	FASTQ	WGS	
Yamnaya	Yamnaya	4,900*	-	1	FASTQ	WGS	
McColl_Group1 ("Hoabinhnian")	La368, Ma911	7,040 - 2,865*	-	2	FASTQ	WGS, Capture	McColl *et al*. (2018); (*12*)
McColl_Group2	La364, La727, La898, Ma912, Vt833, Vt880	~10,000 - 2,000*	-	6	FASTQ	WGS, Capture	
McColl_Group3	Vt777, Vt779, Vt781, Vt796, Vt808	2,275 - 2,242*	-	4	FASTQ	WGS	
McColl_Group3_1	Th531, Vt719	1,687 - 223*	-	1	FASTQ	WGS, Capture	

McColl_Group4	Th519, Th521, Th530, Th703	1,792 - 1,756*	-	4	FASTQ	WGS	
McColl_Group4_1	Vt778	2,549*	-	2	FASTQ	WGS	
McColl_Group5	In661, In662	2,152 - 1,917*	-	2	FASTQ	WGS, Capture	
McColl_Group6	Ma554, Ma555, Phl534	1,877 - 299*	-	3	FASTQ	WGS, Capture	
ARS008	ARS008	3,172	-	1	FASTQ	WGS	Jeong *et al*. (2018); (*18*)
ARS026	ARS026	2,962	-	1	FASTQ	WGS	
DevilsCave_N	NEO235, NEO236, NEO237, NEO238, NEO239, NEO240	7,658 - 7,515	NEO235, NEO237	4	FASTQ	WGS	Sikora et al. (2019); (*19*)
Ekven_IA	NEO241, NEO242, NEO243, NEO246, NEO247, NEO248, NEO249, NEO250, NEO251, NEO253	2,229 - 1,646	-	10	FASTQ	WGS	
Kolyma_M	Kolyma1	9,769	-	1	FASTQ	WGS	
Yana_MED	Yana_young	766	-	1	FASTQ	WGS	
Yana_UP	Yana1, Yana2	31,630	-	2	FASTQ	WGS	
Magadan_BA	M9984, M0831	3,065 - 3,000	-	2	FASTQ	WGS	
Uelen_IA	NEO234, NEO233	2,816 - 1,774	-	2	FASTQ	WGS	
UstBelaya_EBA	NEO230, NEO231, NEO232, NEO298	4,862 - 4,546	-	4	FASTQ	WGS	
UstBelaya_MED	NEO299	598	-	1	FASTQ	WGS	
UstBelaya_N	NEO229	6,579	-	1	FASTQ	WGS	
AR_EN	WQM4, ZLNR-2	7,443 -7,350	-	2	FASTQ	WGS	Ning *et al*. (2020); (*20*)
AR_IA	ZLNR-1	1,843	-	1	FASTQ	WGS	
AR_Xianbei_IA	MGS-M6, MGS-M7L, MGS-M7R	1,800*	MGS-M7L	2	FASTQ	WGS	
HMMH_MN	HMF32	5,615	-	1	FASTQ	WGS	
Miaozigou_MN	MZGM10-1, MZGM16, MZGM25-2	5,500*	-	3	FASTQ	WGS	
Shimao_LN	SM-SGDLM27, SM-SGDLM6, SM-SGDLM7X	4064 - 4,000*	-	3	FASTQ	WGS	
Upper_YR_IA	DCZ-M17IV, DCZ-M21II, DCZ-M22IV, DCZ-M6	2,000 - 1,852*	-	4	FASTQ	WGS	
Upper_YR_LN	JCKM1-1, LJM14, LJM2, LJM25, LJM3, LJM4, LJM5	4,000 - 3,800*	LJM5	6	FASTQ	WGS	
WLR_BA	91KLH11, 91KLH18	2,813 - 2,650*	-	2	FASTQ	WGS	
WLR_BA_o	91KLM2	2,650*	-	1	FASTQ	WGS	
WLR_LN	EDM124, EDM139, EDM176	4,000 - 3,554	-	3	FASTQ	WGS	
WLR_MN	BLSM27S, BLSM41, BLSM45	5,300 - 5,168	-	3	FASTQ	WGS	
YR_LBIA	HJTM115, HJTW13, JXNTM2, JXNTM23, LGM41, LGM79	3,000 - 2,155*	-	6	FASTQ	WGS	

YR_LN	HJTM107, HJTM109, PLTM310, PLTM311, PLTM312, PLTM313, WD-WT1H16, WD-WT5M2	4,110 - 3,700*	PLTM312	7	FASTQ	WGS	
YR_MN	WGH35-1, WGM20, WGM35, WGM43, WGM70, WGM76S, WGM94, XW-M1R18	6,056 - 5,180	-	8	FASTQ	WGS	
Xitoucun	L5705, L5700, L5692, L5706, L5704, L5703, L5701	4,572 - 4,329	-	7	BAM	Capture	Yang *et al.* (2020); (*21*)
Xiaojingshan	XJS1309_M7, XJS1311_M16, XJS1309_M4	7,861 - 7,797	-	3	BAM	Capture	
Tanshishan	L7415, L7417, L4698, L5694	4,472 - 4,318	-	4	BAM	Capture	
Suogang	SuogangB1, SuogangB3	4,550*	-	2	BAM	Capture	
-	Boshan	8,180	-	1	BAM	Capture	
-	Yumin	8,375	-	1	BAM	Capture	
-	Xiaogao	8,684	-	1	BAM	Capture	
-	Liangdao1	8,190	-	1	BAM	Capture	
-	Bianbian	9,513	-	1	BAM	Capture	
-	Liangdao2	7,575	-	1	BAM	Capture	
-	Chuanyun	308	-	1	BAM	Capture	
-	Qihe	8,394	-	1	BAM	Capture	

[a]The values show the most recent and oldest ages if multiple samples were sequenced from the same site in a previous study. The asterisks indicate that the age is not calibrated.

Table S5. Modelling on the genetic ancestry of the Jomon individuals as a mixture of two sources by *qpWave* and *qpAdm*.

Left populations:<br>- Jomon<br>- Hoabinhian<br>- DevilsCave_N	*qpWave*						*qpAdm* (Target: Jomon)		
	Rank 0 (Single ancestor)		Rank 1 (Two ancestors)		Rank 2 (Full model)		Tail probability	Admixture proportions (± 1 standard error)	
	Tail	Tail-diff	Tail	Tail-diff	Tail	Tail-diff		Hoabinhian	DevilsCave_N
	1.20E-169	1	2.75E-05	1.85E-168	1	2.758E-05	2.81E-05	-	-

Table S6. Results for f_4-tests with the form of f_4(Mbuti, Yana; Jomon, *X*).

Pop1	Pop2	Pop3	Pop4 (*X*)	f_4	sd	*Z*-score (bold if Z < -3.0)
Mbuti	Yana	Jomon	Ami	-0.000755	0.000263	-2.871
			**Atayal**	**-0.001147**	**0.000307**	**-3.729**
			**Dai**	**-0.000892**	**0.000244**	**-3.656**
			**Daur**	**-0.000933**	**0.000308**	**-3.032**
			**Han**	**-0.000954**	**0.000263**	**-3.628**
			**Japanese**	**-0.000804**	**0.000239**	**-3.366**
			Korean	-0.000772	0.000258	-2.998
			**Lahu**	**-0.000948**	**0.000266**	**-3.561**
			**Miao**	**-0.00087**	**0.000263**	**-3.312**
			Mongola	-0.000819	0.000293	-2.796
			Oroqen	-0.000674	0.000271	-2.49
			She	-0.000734	0.000287	-2.562
			**Tu**	**-0.001025**	**0.000263**	**-3.901**
			**Tujia**	**-0.001048**	**0.000271**	**-3.867**
			**Xibo**	**-0.000937**	**0.000261**	**-3.593**
			**Burmese**	**-0.001301**	**0.000322**	**-4.041**
			**Cambodian**	**-0.001120**	**0.000267**	**-4.195**
			**Dusun**	**-0.001059**	**0.000270**	**-3.914**
			**Kinh**	**-0.001085**	**0.000276**	**-3.931**
			**Thai**	**-0.001248**	**0.000269**	**-4.643**
			Yi	-0.00071	0.000269	-2.639
			DevilsCave_N	-0.00003	0.00026	-0.126
			Shamanka_EN	0.00038	0.00024	1.574
			Lokomotiv_EN	0.00035	0.00027	1.287

Table S7. Modelling on the genetic ancestry of the Yayoi individuals as a mixture of two sources by *qpWave* and *qpAdm*.

**Left populations:**<br>- **Yayoi**<br>- **Jomon**<br>- **Additional source**	*qpWave*						*qpAdm* **(Target: Yayoi)**		
	**Rank 0 (Single ancestor)**		**Rank 1 (Two ancestors)**		**Rank 2 (Full model)**		**Tail probability**	**Admixture proportions (± 1 standard error)**	
**Additional source**	**Tail**	**Tail-diff**	**Tail**	**Tail-diff**	**Tail**	**Tail-diff**		**Jomon**	**Additional source**
**Right populations without a subset of Jomon**									
**WLR_BA_o**	**5.39E-23**	**1**	**3.61E-01**	**4.15E-25**	**1**	**3.61E-01**	**3.64E-01**	**0.584 ± 0.076**	**0.416 ± 0.076**
**HMMH_MN**	**2.72E-17**	**1**	**2.01E-01**	**1.01E-18**	**1**	**2.01E-01**	**2.02E-01**	**0.506 ± 0.088**	**0.494 ± 0.088**
UstBelaya_EBA	2.43E-23	1	4.64E-05	1.05E-20	1	4.64E-05	0.00E+00	-	-
Ekven_IA	1.32E-27	1	1.45E-05	1.47E-24	1	1.45E-05	0.00E+00	-	-
Even	6.99E-14	1	2.77E-02	5.62E-14	1	2.77E-02	2.80E-02	-	-
**Lokomotiv_EN**	**3.15E-23**	**1**	**8.01E-02**	**2.24E-24**	**1**	**8.01E-02**	**8.10E-02**	**0.550 ± 0.101**	**0.450 ± 0.101**
Shamanka_EN	3.24E-33	1	1.86E-02	6.80E-34	1	1.86E-02	1.90E-02	-	-
**Northeast Asian ancestry (WLR_BA_o + HMMH_MN)**	**3.46E-28**	**1**	**2.64E-01**	**2.77E-30**	**1**	**2.64E-01**	**2.66E-01**	**0.613 ± 0.074**	**0.387 ± 0.074**
**Right populations with a subset of Jomon**									
**WLR_BA_o**	**0**	**1**	**4.65E-01**	**0**	**1**	**4.65E-01**	**4.67E-01**	**0.542 ± 0.021**	**0.458 ± 0.021**
**HMMH_MN**	**0**	**1**	**3.47E-01**	**0**	**1**	**3.47E-01**	**3.49E-01**	**0.491 ± 0.021**	**0.509 ± 0.021**
UstBelaya_EBA	0	1	9.02E-05	0	1	9.02E-05	0.00E+00	-	-
Ekven_IA	0	1	8.23E-07	0	1	8.23E-07	0.00E+00	-	-
Even	0	1	3.22E-02	0	1	3.22E-02	3.30E-02	-	-
**Lokomotiv_EN**	**0**	**1**	**1.62E-01**	**0**	**1**	**1.62E-01**	**1.64E-01**	**0.598 ± 0.019**	**0.402 ± 0.019**
**Shamanka_EN**	**0**	**1**	**5.19E-02**	**0**	**1**	**5.19E-02**	**5.30E-02**	**0.623 ± 0.019**	**0.377 ± 0.019**
**Northeast Asian ancestry (WLR_BA_o + HMMH_MN)**	**3.46E-28**	**0**	**1**	**3.94E-01**	**0**	**1**	**3.96E-01**	**0.587 ± 0.02**	**0.413 ± 0.02**

Note: Source population is highlighted by bold if the admixture is supported from modelling with a given set of the reference populations or by bold and red if the admixture is supported from modelling with and without a subset of Jomon included in the reference populations.

Table S8. Modelling on the genetic ancestry of the Kofun individuals as a mixture of two sources by *qpWave* and *qpAdm*.

**Left populations:**<br>- **JpIw**<br>- **Jomon**<br>- **Additional source**	***qpWave***						***qpAdm*** **(Target: JpIw)**		
	**Rank 0 (Single ancestor)**		**Rank 1 (Two ancestors)**		**Rank 2 (Full model)**		**Tail probability**	**Admixture proportions (± 1 standard error)**	
								**Jomon**	**Additional source**
**Additional source**	**Tail**	**Tail-diff**	**Tail**	**Tail-diff**	**Tail**	**Tail-diff**			
**Right populations without a subset of Jomon**									
WLR_BA_o	4.25E-108	1	1.64E-07	2.87E-104	1	1.64E-07	0.00E+00	-	-
HMMH_MN	6.21E-107	1	7.73E-14	2.20E-96	1	7.73E-14	0.00E+00	-	-
Lokomotiv_EN	8.86E-133	1	3.65E-24	8.34E-112	1	3.65E-24	0.00E+00	-	-
Northeast Asian ancestry (WLR_BA_o + HMMH_MN)	4.09E-112	1	1.42E-15	8.27E-100	1	1.42E-15	0.00E+00	-	-
**Right populations with a subset of Jomon**									
WLR_BA_o	0	1	5.26E-08	0	1	5.26E-08	0.00E+00	-	-
HMMH_MN	0	1	1.60E-17	0	1	1.60E-17	0.00E+00	-	-
Lokomotiv_EN	0	1	6.75E-53	0	1	6.75E-53	0.00E+00	-	-
Northeast Asian ancestry (WLR_BA_o + HMMH_MN)	0	1	9.55E-18	0	1	9.55E-18	0.00E+00	-	-

Table S9. Modelling on the genetic ancestry of the Kofun individuals as a mixture of two sources by *qpWave* and *qpAdm*.

**Left populations:**<br>- **JpIw**<br>- **Yayoi**<br>- **Additional source**	***qpWave***						***qpAdm* (Target: JpIw)**		
	**Rank 0 (Single ancestor)**		**Rank 1 (Two ancestors)**		**Rank 2 (Full model)**		**Tail probability**	**Admixture proportions (± 1 standard error)**	
**Additional source**	**Tail**	**Tail-diff**	**Tail**	**Tail-diff**	**Tail**	**Tail-diff**		**Yayoi**	**Additional source**
**Right populations without a subset of Jomon**									
**Han**	**2.59E-25**	**1**	**2.25E-01**	**3.42E-27**	**1**	**2.25E-01**	**2.32E-01**	**0.775 ± 0.046**	**0.225 ± 0.046**
**Korean**	**2.47E-20**	**1**	**6.01E-02**	**3.44E-21**	**1**	**6.01E-02**	**6.30E-02**	**0.748 ± 0.059**	**0.225 ± 0.059**
**YR_LBIA**	**4.43E-24**	**1**	**4.02E-01**	**2.54E-26**	**1**	**4.02E-01**	**4.06E-01**	**0.693 ± 0.056**	**0.307 ± 0.056**
Uygur	1.43E-290	1	5.20E-02	2.49E-294	1	5.20E-02	6.20E-02	1.031 ± 0.004	-0.031 ± 0.004
Burmese	2.96E-09	1	3.23E-01	1.89E-10	1	3.23E-01	3.27E-01	2.215 ± 1.328	-1.215 ± 1.328
Atayal	2.50E-51	1	1.37E-09	1.58E-44	1	1.37E-09	0.00E+00	-	-
Daur	2.26E-12	1	5.51E-03	1.49E-11	1	5.51E-03	7.00E-03	-	-
Lahu	1.62E-15	1	7.48E-05	8.16E-13	1	7.48E-05	0.00E+00	-	-
Miao	1.21E-23	1	2.94E-03	4.94E-23	1	2.94E-03	3.00E-03	-	-
Mongola	1.50E-13	1	1.21E-02	3.38E-13	1	1.21E-02	1.40E-02	-	-
Oroqen	7.65E-18	1	3.44E-05	7.13E-15	1	3.44E-05	0.00E+00	-	-
She	4.08E-26	1	7.35E-03	4.56E-26	1	7.35E-03	8.00E-03	-	-
Tu	7.38E-17	1	1.07E-05	2.55E-13	1	1.07E-05	0.00E+00	-	-
Tujia	3.17E-21	1	2.30E-02	1.37E-21	1	2.30E-02	2.50E-02	-	-
Xibo	4.20E-11	1	9.19E-03	1.84E-10	1	9.19E-03	1.00E-02	-	-
Cambodian	1.19E-33	1	7.78E-05	1.43E-31	1	7.78E-05	0.00E+00	-	-
Dusun	7.57E-51	1	9.31E-11	8.12E-43	1	9.31E-11	0.00E+00	-	-
Kinh	2.91E-36	1	1.43E-09	3.37E-29	1	1.43E-09	0.00E+00	-	-
Thai	2.04E-32	1	9.29E-10	4.41E-25	1	9.29E-10	0.00E+00	-	-
Yi	4.41E-14	1	1.08E-03	1.46E-12	1	1.08E-03	1.00E-03	-	-
Aleut	5.37E-292	1	3.52E-02	1.56E-295	1	3.52E-02	4.30E-02	-	-
Altaian	2.86E-70	1	1.35E-02	1.43E-71	1	1.35E-02	1.90E-02	-	-
Eskimo_Chaplin	1.84E-56	1	1.03E-03	3.57E-56	1	1.03E-03	2.00E-03	-	-
Eskimo_Naukan	4.26E-110	1	7.34E-03	1.40E-111	1	7.34E-03	1.00E-02	-	-
Eskimo_Sireniki	1.84E-107	1	4.27E-03	1.25E-108	1	4.27E-03	6.00E-03	-	-
Even	3.30E-75	1	6.82E-03	3.24E-76	1	6.82E-03	9.00E-03	-	-
Hezhen	1.36E-14	1	3.25E-04	1.57E-12	1	3.25E-04	0.00E+00	-	-
Itelman	3.62E-73	1	6.28E-04	6.56E-73	1	6.28E-04	1.00E-03	-	-

Tubalar	3.21E-234	1	2.19E-02	3.26E-237	1	2.19E-02	2.70E-02	-	-
Ulchi	7.99E-18	1	2.67E-04	8.26E-16	1	2.67E-04	0.00E+00	-	-
ARS008	7.96E-65	1	6.54E-04	1.87E-64	1	6.54E-04	1.00E-03	-	-
ARS026	7.70E-244	1	3.81E-02	3.37E-247	1	3.81E-02	4.80E-02	-	-
Lokomotiv_EN	1.25E-47	1	6.86E-06	1.02E-44	1	6.86E-06	0.00E+00	-	-
Shamanka_EBA	2.88E-104	1	1.16E-04	1.48E-103	1	1.16E-04	0.00E+00	-	-
Shamanka_EN	2.59E-62	1	4.79E-07	2.09E-58	1	4.79E-07	0.00E+00	-	-
Ust_Ida_EBA	3.28E-128	1	1.86E-04	5.62E-128	1	1.86E-04	0.00E+00	-	-
Ust_Ida_LN	1.95E-142	1	8.41E-04	4.36E-143	1	8.41E-04	1.00E-03	-	-
DevilsCave_N	2.65E-17	1	3.10E-08	3.40E-11	1	3.10E-08	0.00E+00	-	-
UstBelaya_N	1.86E-26	1	7.33E-06	4.68E-23	1	7.33E-06	0.00E+00	-	-
UstBelaya_EBA	8.94E-112	1	2.94E-04	1.30E-111	1	2.94E-04	0.00E+00	-	-
Ekven_IA	3.18E-185	1	7.52E-04	3.94E-186	1	7.52E-04	1.00E-03	-	-
Yana_MED	8.01E-63	1	9.30E-04	1.34E-62	1	9.30E-04	1.00E-03	-	-
McColl_Group2	3.58E-48	1	3.29E-08	8.99E-43	1	3.29E-08	0.00E+00	-	-
McColl_Group3	1.05E-39	1	1.06E-09	1.41E-32	1	1.06E-09	0.00E+00	-	-
McColl_Group4	1.29E-18	1	1.99E-02	8.81E-19	1	1.99E-02	2.20E-02	-	-
Boshan	6.25E-13	1	3.10E-04	9.03E-11	1	3.10E-04	0.00E+00	-	-
AR_EN	2.07E-19	1	3.81E-09	1.95E-12	1	3.81E-09	0.00E+00	-	-
AR_Xianbei_IA	1.81E-21	1	8.07E-08	7.08E-16	1	8.07E-08	0.00E+00	-	-
HMMH_MN	4.11E-22	1	3.87E-10	3.42E-14	1	3.87E-10	0.00E+00	-	-
WLR_MN	1.34E-17	1	2.13E-07	2.46E-12	1	2.13E-07	0.00E+00	-	-
WLR_LN	4.27E-15	1	4.75E-02	1.49E-15	1	4.75E-02	4.80E-02	-	-
WLR_BA	9.14E-14	1	3.56E-03	8.26E-13	1	3.56E-03	4.00E-03	-	-
WLR_BA_o	2.85E-15	1	1.56E-07	8.04E-10	1	1.56E-07	0.00E+00	-	-
Miaozigou_MN	1.61E-22	1	6.20E-06	6.41E-19	1	6.20E-06	0.00E+00	-	-
Shimao_LN	3.10E-19	1	3.91E-06	2.55E-15	1	3.91E-06	0.00E+00	-	-
Upper_YR_LN	1.20E-37	1	2.53E-10	7.83E-30	1	2.53E-10	0.00E+00	-	-
Upper_YR_IA	5.65E-20	1	3.64E-02	1.56E-20	1	3.64E-02	3.80E-02	-	-
YR_MN	6.12E-25	1	6.12E-06	2.08E-21	1	6.12E-06	0.00E+00	-	-
YR_LN	7.84E-27	1	4.77E-02	8.03E-28	1	4.77E-02	4.90E-02	-	-
**Right populations with a subset of Jomon**									
**Han**	**2.45E-240**	**1**	**2.54E-01**	**1.85E-245**	**1**	**2.54E-01**	**2.62E-01**	**0.736 ± 0.017**	**0.264 ± 0.017**
**Korean**	**2.37E-190**	**1**	**8.36E-02**	**2.28E-194**	**1**	**8.36E-02**	**8.70E-02**	**0.710 ± 0.020**	**0.290 ± 0.020**
**YR_LBIA**	**3.54E-214**	**1**	**5.11E-01**	**1.01E-219**	**1**	**5.11E-01**	**5.16E-01**	**0.680 ± 0.020**	**0.320 ± 0.020**

Uygur	0.00E+00	1	3.93E-93	0.00E+00	1	3.93E-93	0.00E+00	-	-
Burmese	4.59E-154	1	6.19E-05	8.17E-154	1	6.19E-05	0.00E+00	-	-
Atayal	3.73E-202	1	9.63E-33	6.28E-173	1	9.63E-33	0.00E+00	-	-
Daur	1.32E-178	1	7.16E-10	4.81E-173	1	7.16E-10	0.00E+00	-	-
Lahu	1.80E-208	1	3.72E-05	2.22E-208	1	3.72E-05	0.00E+00	-	-
Miao	8.66E-214	1	4.22E-03	3.28E-216	1	4.22E-03	5.00E-03	-	-
Mongola	6.37E-202	1	5.26E-12	3.14E-194	1	5.26E-12	0.00E+00	-	-
Oroqen	8.07E-191	1	5.65E-17	8.49E-178	1	5.65E-17	0.00E+00	-	-
She	5.35E-230	1	3.56E-03	1.98E-232	1	3.56E-03	4.00E-03	-	-
Tu	3.40E-230	1	5.34E-14	1.49E-220	1	5.34E-14	0.00E+00	-	-
Tujia	8.78E-210	1	3.60E-02	2.08E-213	1	3.60E-02	3.90E-02	-	-
Xibo	8.61E-186	1	8.16E-06	8.80E-185	1	8.16E-06	0.00E+00	-	-
Cambodian	1.33E-218	1	2.61E-32	6.58E-190	1	2.61E-32	0.00E+00	-	-
Dusun	1.26E-232	1	2.01E-30	6.14E-206	1	2.01E-30	0.00E+00	-	-
Kinh	2.36E-250	1	9.32E-15	5.06E-240	1	9.32E-15	0.00E+00	-	-
Thai	1.62E-249	1	6.45E-23	1.19E-230	1	6.45E-23	0.00E+00	-	-
Yi	3.62E-190	1	1.29E-03	8.82E-192	1	1.29E-03	1.00E-03	-	-
Aleut	0.00E+00	1	1.34E-94	0.00E+00	1	1.34E-94	0.00E+00	-	-
Altaian	8.80E-223	1	4.15E-70	7.54E-156	1	4.15E-70	0.00E+00	-	-
Eskimo_Chaplin	1.21E-217	1	1.23E-53	2.74E-167	1	1.23E-53	0.00E+00	-	-
Eskimo_Naukan	6.49E-315	1	1.61E-80	8.24E-238	1	1.61E-80	0.00E+00	-	-
Eskimo_Sireniki	4.10E-286	1	3.25E-76	2.98E-213	1	3.25E-76	0.00E+00	-	-
Even	3.83E-283	1	1.69E-59	3.95E-227	1	1.69E-59	0.00E+00	-	-
Hezhen	4.05E-192	1	5.55E-11	1.86E-185	1	5.55E-11	0.00E+00	-	-
Itelman	1.27E-225	1	3.65E-68	1.18E-160	1	3.65E-68	0.00E+00	-	-
Tubalar	0.00E+00	1	5.34E-94	0.00E+00	1	5.34E-94	0.00E+00	-	-
Ulchi	1.41E-157	1	1.73E-15	7.15E-146	1	1.73E-15	0.00E+00	-	-
ARS008	1.42E-212	1	1.61E-63	3.12E-152	1	1.61E-63	0.00E+00	-	-
ARS026	0.00E+00	1	3.55E-97	5.64E-293	1	3.55E-97	0.00E+00	-	-
Lokomotiv_EN	3.94E-225	1	5.71E-43	1.31E-185	1	5.71E-43	0.00E+00	-	-
Shamanka_EBA	1.02E-289	1	1.44E-73	1.56E-219	1	1.44E-73	0.00E+00	-	-
Shamanka_EN	8.56E-266	1	2.16E-46	5.52E-223	1	2.16E-46	0.00E+00	-	-
Ust_Ida_EBA	4.28E-296	1	4.35E-78	2.22E-221	1	4.35E-78	0.00E+00	-	-
Ust_Ida_LN	0.00E+00	1	7.61E-84	2.44E-253	1	7.61E-84	0.00E+00	-	-
DevilsCave_N	1.91E-149	1	8.45E-10	9.93E-144	1	8.45E-10	0.00E+00	-	-

UstBelaya_N	1.04E-173	1	1.73E-22	7.72E-155	1	1.73E-22	0.00E+00	-	-
UstBelaya_EBA	7.93E-304	1	3.89E-73	4.00E-234	1	3.89E-73	0.00E+00	-	-
Ekven_IA	0.00E+00	1	1.09E-85	0.00E+00	1	1.09E-85	0.00E+00	-	-
Yana_MED	9.84E-198	1	1.84E-62	2.14E-138	1	1.84E-62	0.00E+00	-	-
McColl_Group2	2.99E-239	1	1.01E-41	4.56E-201	1	1.01E-41	0.00E+00	-	-
McColl_Group3	1.33E-199	1	2.63E-31	7.88E-172	1	2.63E-31	0.00E+00	-	-
McColl_Group4	4.42E-182	1	1.33E-19	2.95E-166	1	1.33E-19	0.00E+00	-	-
Boshan	6.41E-122	1	4.22E-04	2.45E-122	1	4.22E-04	0.00E+00	-	-
AR_EN	1.00E-163	1	5.58E-13	1.02E-154	1	5.58E-13	0.00E+00	-	-
AR_Xianbei_IA	8.94E-167	1	4.32E-16	1.65E-154	1	4.32E-16	0.00E+00	-	-
HMMH_MN	1.80E-159	1	3.73E-16	4.42E-147	1	3.73E-16	0.00E+00	-	-
WLR_MN	6.59E-170	1	3.22E-07	3.42E-167	1	3.22E-07	0.00E+00	-	-
**WLR_LN**	**2.70E-163**	**1**	**7.90E-02**	**4.66E-167**	**1**	**7.90E-02**	**8.00E-02**	**0.440 ± 0.026**	**0.560 ± 0.026**
WLR_BA	1.75E-166	1	6.91E-03	7.88E-169	1	6.91E-03	7.00E-03	-	-
WLR_BA_o	5.58E-142	1	2.63E-07	6.31E-139	1	2.63E-07	0.00E+00	-	-
Miaozigou_MN	7.84E-183	1	6.67E-07	1.43E-180	1	6.67E-07	0.00E+00	-	-
Shimao_LN	1.67E-172	1	7.90E-06	2.24E-171	1	7.90E-06	0.00E+00	-	-
Upper_YR_LN	1.11E-240	1	9.54E-16	2.97E-229	1	9.54E-16	0.00E+00	-	-
**Upper_YR_IA**	**1.62E-210**	**1**	**5.73E-02**	**1.96E-214**	**1**	**5.73E-02**	**5.90E-02**	**0.662 ± 0.021**	**0.338 ± 0.021**
YR_MN	3.78E-208	1	6.28E-06	3.64E-207	1	6.28E-06	0.00E+00	-	-
YR_LN	4.83E-225	1	4.53E-02	6.56E-229	1	4.53E-02	4.70E-02	-	-

Note: Source population is highlighted by bold if the admixture is supported from modelling with a given set of the reference populations or by bold and red if the admixture is supported from modelling with and without a subset of Jomon included in the reference populations.

Table S10 Modelling on the genetic ancestry of the Kofun individuals as a mixture of three sources by *qpWave* and *qpAdm*.

Left populations: - JpIw - Jomon - Two more sources		*qpWave*					*qpAdm* (Target: JpIw)			
		Probability	Rank				Tail	Admixture proportions (± 1 standard error)		
Second source	Third source		0	1	2	3		Jomon	Second source	Third source
**Right populations without a subset of Jomon**										
Northeast Asian ancestry (WLR_BA_o + HMMH_MN)	**Han**	**Tail**	**1.20E-155**	**1.29E-19**	**3.91E-01**	**1**	**3.94E-01**	**0.150 ± 0.038**	**0.215 ± 0.088**	**0.636 ± 0.068**
		**Tail-diff**	**1**	**8.23E-141**	**2.00E-21**	**3.91E-01**				
	Korean	Tail	6.17E-138	7.78E-15	8.84E-02	1	9.00E-02	0.282 ± 0.076	-0.415 ± 0.249	1.133 ± 0.193
		Tail-diff	1	4.75E-128	1.61E-15	8.84E-02				
	YR_LBIA	Tail	1.33E-155	2.09E-13	7.82E-01	1	7.83E-01	0.216 ± 0.043	-0.306 ± 0.139	1.090 ± 0.112
		Tail-diff	1	1.45E-147	1.83E-15	7.82E-01				
**Right populations with a subset of Jomon**										
Northeast Asian ancestry (WLR_BA_o + HMMH_MN)	**Han**	**Tail**	**0**	**3.592E-27**	**4.772E-01**	**1**	**4.81E-01**	**0.139 ± 0.010**	**0.231 ± 0.071**	**0.631 ± 0.066**
		**Tail-diff**	**1**	**0**	**1.132E-29**	**4.772E-01**				
	Korean	Tail	0	1.435E-19	2.149E-02	1	2.20E-02	-	-	-
		Tail-diff	1	0	8.008E-20	2.149E-02				
	YR_LBIA	Tail	0	2.725E-18	2.286E-01	1	2.34E-01	0.134 ± 0.010	-0.167 ± 0.108	1.034 ± 0.104
		Tail-diff	1	0	7.341E-20	2.286E-01				

Note: Source population is highlighted by bold if the admixture is supported from modelling with a given set of the reference populations or by bold and red if the admixture is supported from modelling with and without a subset of Jomon included in the reference populations.

Table S11. Testing fittings of three-way versus two-way admixture to the genetic ancestry of the Kofun by *qpAdm* with the third source represented by Han.

**Left populations:**<br>**Target: JpIw**<br>**Sources:**<br>- **Jomon**<br>- **Northeast Asian ancestry (WLR_BA_o + HMMH_MN)**<br>- **Han**<br><br>**Models**	**Degree of freedom**	**Tail probability**	x^2	***P*-value for the nested model (Three-way vs. Two-way models)**
**Right populations without a subset of Jomon**				
Three-way (Jomon, Northeast Asian, and Han)	5	3.94E-1	5.186	-
Two-way (Jomon and Han)	6	9.90E-2	10.665	1.90E-2
Two-way (Northeast Asian and Han)	6	2.00E-2	20.541	8.91E-5
Two-way (Jomon and Northeast Asian)	6	2.42E-15	80.822	0.00E+00
**Right populations with a subset of Jomon**				
Three-way (Jomon, Northeast Asian, and Han)	6	4.81E-1	5.505	-
Two-way (Jomon and Han)	7	4.58E-2	14.319	2.99E-3
Two-way (Northeast Asian and Han)	7	2.05E-29	151.414	0.00E+00
Two-way (Jomon and Northeast Asian)	7	1.68E-17	94.215	0.00E+00

Table S12. Modelling on the genetic ancestry of the Kofun individuals as a mixture of two sources (Jomon and additional source) by *qpWave* and *qpAdm*.

Left populations: - JpIw - Jomon - Additional source	*qpWave*						*qpAdm* (Target: JpIw)		
	Rank 0 (Single ancestor)		Rank 1 (Two ancestors)		Rank 2 (Full model)		Tail probability	Admixture proportions (± 1 standard error)	
								Jomon	Additional source
Additional source	Tail	Tail-diff	Tail	Tail-diff	Tail	Tail-diff			
**Right populations without a subset of Jomon**									
**Han**	**3.43E-145**	**1**	**9.59E-02**	**1.80E-148**	**1**	**9.59E-02**	**9.70E-02**	**0.212 ± 0.031**	**0.788 ± 0.031**
**YR_LBIA**	**6.23E-149**	**1**	**1.41E-01**	**1.74E-152**	**1**	**1.41E-01**	**1.42E-01**	**0.149 ± 0.027**	**0.851 ± 0.027**
Burmese	4.28E-98	1	2.99E-01	1.15E-101	1	2.99E-01	3.00E-01	-0.169 ± 0.088	1.169 ± 0.099
Atayal	5.97E-157	1	3.04E-35	7.29E-125	1	3.04E-35	0.00E+00	0.304 ± 0.054	0.696 ± 0.054
Daur	8.79E-98	1	6.46E-04	7.31E-98	1	6.46E-04	1.00E-03	-0.355 ± 0.135	1.355 ± 0.135
Korean	1.92E-131	1	1.92E-02	1.16E-133	1	1.92E-02	2.00E-02	0.177 ± 0.035	0.823 ± 0.035
Lahu	5.62E-114	1	7.38E-05	3.80E-113	1	7.38E-05	0.00E+00	0.054 ± 0.048	0.946 ± 0.048
Miao	1.28E-132	1	3.31E-04	1.03E-132	1	3.31E-04	0.00E+00	0.184 ± 0.036	0.816 ± 0.036
Mongola	1.41E-101	1	1.71E-03	3.36E-102	1	1.71E-03	2.00E-03	-0.247 ± 0.076	1.247 ± 0.076
Oroqen	1.82E-104	1	2.47E-09	1.23E-98	1	2.47E-09	0.00E+00	-0.398 ± 0.116	1.398 ± 0.116
She	1.61E-132	1	6.10E-04	6.37E-133	1	6.10E-04	1.00E-03	0.243 ± 0.033	0.757 ± 0.033
Tu	1.46E-106	1	4.40E-08	4.25E-102	1	4.40E-08	0.00E+00	-0.268 ± 0.096	1.268 ± 0.096
Tujia	3.35E-123	1	1.23E-02	4.25E-125	1	1.23E-02	1.30E-02	0.191 ± 0.034	0.809 ± 0.034
Uygur	0.00E+00	1	1.23E-23	0.00E+00	1	1.23E-23	0.00E+00	1.565 ± 0.053	-0.565 ± 0.053
Xibo	1.40E-106	1	4.41E-03	9.38E-108	1	4.41E-03	5.00E-03	-0.093 ± 0.063	1.093 ± 0.063
Cambodian	4.07E-113	1	1.51E-15	7.58E-101	1	1.51E-15	0.00E+00	-0.799 ± 0.189	1.799 ± 0.189
Dusun	5.56E-159	1	1.17E-41	2.03E-120	1	1.17E-41	0.00E+00	0.177 ± 0.057	0.823 ± 0.057
Kinh	4.45E-142	1	1.47E-19	6.80E-126	1	1.47E-19	0.00E+00	0.19 ± 0.042	0.81 ± 0.042
Thai	2.38E-120	1	2.37E-20	3.48E-103	1	2.37E-20	0.00E+00	-0.286 ± 0.102	1.286 ± 0.102
Yi	7.39E-110	1	2.45E-04	1.39E-109	1	2.45E-04	0.00E+00	0.074 ± 0.042	0.926 ± 0.042
Aleut	0.00E+00	1	4.80E-25	0.00E+00	1	4.80E-25	0.00E+00	1.544 ± 0.051	-0.544 ± 0.051
Altaian	2.35E-156	1	1.52E-22	3.35E-137	1	1.52E-22	0.00E+00	3.221 ± 0.796	-2.221 ± 0.796
Eskimo_Chaplin	7.07E-141	1	9.23E-23	2.14E-121	1	9.23E-23	0.00E+00	88.014 ± 6552.437	-87.014 ± 6552.437
Eskimo_Naukan	5.20E-203	1	4.19E-24	1.58E-182	1	4.19E-24	0.00E+00	4.443 ± 1.317	-3.443 ± 1.317
Eskimo_Sireniki	3.40E-184	1	1.57E-30	4.81E-157	1	1.57E-30	0.00E+00	5.059 ± 1.801	-4.059 ± 1.801
Even	5.07E-162	1	5.12E-19	1.56E-146	1	5.12E-19	0.00E+00	-6.139 ± 3.613	7.139 ± 3.613
Hezhen	1.22E-101	1	8.29E-05	9.75E-101	1	8.29E-05	0.00E+00	-0.216 ± 0.076	1.216 ± 0.076
Itelman	7.29E-154	1	7.04E-29	3.20E-128	1	7.04E-29	0.00E+00	11.759 ± 13.954	-10.759 ± 13.954

Tubalar	1.22E-295	1	2.58E-24	2.37E-275	1	2.58E-24	0.00E+00	1.735 ± 0.078	-0.735 ± 0.078
Ulchi	1.91E-106	1	1.10E-06	1.69E-103	1	1.10E-06	0.00E+00	-0.463 ± 0.113	1.463 ± 0.113
ARS008	1.60E-142	1	3.12E-26	1.66E-119	1	3.12E-26	0.00E+00	41.448 ± 239.75	-40.448 ± 239.75
ARS026	4.31E-303	1	1.84E-24	1.11E-282	1	1.84E-24	0.00E+00	1.524 ± 0.049	-0.524 ± 0.049
Lokomotiv_EN	8.86E-133	1	3.65E-24	8.34E-112	1	3.65E-24	0.00E+00	-0.871 ± 0.205	1.871 ± 0.205
Shamanka_EBA	7.32E-176	1	9.13E-34	2.24E-145	1	9.13E-34	0.00E+00	35.296 ± 108.781	-34.296 ± 108.781
Shamanka_EN	2.12E-145	1	4.85E-41	2.15E-107	1	4.85E-41	0.00E+00	-0.987 ± 0.29	1.987 ± 0.29
Ust_Ida_EBA	1.40E-208	1	2.24E-42	1.57E-169	1	2.24E-42	0.00E+00	10.111 ± 9.893	-9.111 ± 9.893
Ust_Ida_LN	1.33E-208	1	1.83E-35	1.48E-176	1	1.83E-35	0.00E+00	3.669 ± 0.797	-2.669 ± 0.797
DevilsCave_N	2.49E-110	1	1.39E-08	2.31E-105	1	1.39E-08	0.00E+00	-0.104 ± 0.063	1.104 ± 0.063
UstBelaya_N	1.46E-112	1	5.57E-12	5.20E-104	1	5.57E-12	0.00E+00	-0.31 ± 0.074	1.31 ± 0.074
UstBelaya_EBA	1.67E-174	1	1.77E-37	3.01E-140	1	1.77E-37	0.00E+00	14 ± 15.745	-13 ± 15.745
Ekven_IA	6.14E-259	1	8.06E-42	1.17E-220	1	8.06E-42	0.00E+00	3.213 ± 0.594	-2.213 ± 0.594
Yana_MED	1.42E-146	1	1.31E-21	2.60E-128	1	1.31E-21	0.00E+00	-5.453 ± 4.083	6.453 ± 4.083
McColl_Group2	2.49E-126	1	7.88E-32	1.61E-97	1	7.88E-32	0.00E+00	-0.521 ± 0.135	1.521 ± 0.135
McColl_Group3	5.64E-126	1	2.21E-29	1.21E-99	1	2.21E-29	0.00E+00	-0.087 ± 0.034	1.087 ± 0.034
McColl_Group4	3.28E-99	1	1.35E-05	2.16E-97	1	1.35E-05	0.00E+00	-0.234 ± 0.048	1.234 ± 0.048
Boshan	1.32E-101	1	2.95E-04	2.46E-101	1	2.95E-04	0.00E+00	0.005 ± 0.014	0.995 ± 0.014
AR_EN	6.18E-109	1	3.26E-10	3.24E-102	1	3.26E-10	0.00E+00	-0.096 ± 0.034	1.096 ± 0.034
AR_Xianbei_IA	2.53E-111	1	7.78E-12	6.50E-103	1	7.78E-12	0.00E+00	-0.09 ± 0.029	1.09 ± 0.029
HMMH_MN	6.21E-107	1	7.73E-14	2.20E-96	1	7.73E-14	0.00E+00	-0.151 ± 0.071	1.151 ± 0.071
WLR_MN	5.57E-113	1	1.13E-07	5.06E-109	1	1.13E-07	0.00E+00	0.011 ± 0.05	0.989 ± 0.05
WLR_LN	8.34E-110	1	1.35E-02	1.28E-111	1	1.35E-02	1.40E-02	0.049 ± 0.017	0.951 ± 0.017
WLR_BA	1.24E-113	1	9.53E-04	4.37E-114	1	9.53E-04	1.00E-03	0.054 ± 0.03	0.946 ± 0.03
WLR_BA_o	4.25E-108	1	1.64E-07	2.87E-104	1	1.64E-07	0.00E+00	-0.017 ± 0.052	1.017 ± 0.052
Miaozigou_MN	5.55E-128	1	5.24E-09	1.00E-122	1	5.24E-09	0.00E+00	0.074 ± 0.022	0.926 ± 0.022
Shimao_LN	3.66E-119	1	8.73E-08	3.83E-115	1	8.73E-08	0.00E+00	0.104 ± 0.039	0.896 ± 0.039
Upper_YR_LN	3.25E-145	1	9.06E-22	8.85E-127	1	9.06E-22	0.00E+00	0.144 ± 0.038	0.856 ± 0.038
Upper_YR_IA	4.69E-130	1	1.03E-02	6.42E-132	1	1.03E-02	1.10E-02	0.138 ± 0.031	0.862 ± 0.031
YR_MN	2.98E-131	1	2.39E-08	9.85E-127	1	2.39E-08	0.00E+00	0.133 ± 0.034	0.867 ± 0.034
YR_LN	1.30E-149	1	4.88E-03	3.06E-151	1	4.88E-03	5.00E-03	0.173 ± 0.029	0.827 ± 0.029
**Right populations with a subset of Jomon**									
**Han**	**0.00E+00**	**1**	**4.90E-02**	**0.00E+00**	**1**	**4.90E-02**	**5.00E-02**	**0.158 ± 0.009**	**0.842 ± 0.009**
**YR_LBIA**	**0.00E+00**	**1**	**1.51E-01**	**0.00E+00**	**1**	**1.51E-01**	**1.54E-01**	**0.125 ± 0.008**	**0.875 ± 0.008**

Burmese	0.00E+00	1	1.01E-04	0.00E+00	1	1.01E-04	0.00E+00	0.143 ± 0.011	0.857 ± 0.011
Atayal	0.00E+00	1	6.44E-37	0.00E+00	1	6.44E-37	0.00E+00	0.147 ± 0.012	0.853 ± 0.012
Daur	0.00E+00	1	5.13E-11	0.00E+00	1	5.13E-11	0.00E+00	0.15 ± 0.011	0.85 ± 0.011
Korean	0.00E+00	1	2.18E-02	0.00E+00	1	2.18E-02	2.20E-02	0.141 ± 0.01	0.859 ± 0.01
Lahu	0.00E+00	1	1.61E-05	0.00E+00	1	1.61E-05	0.00E+00	0.159 ± 0.01	0.841 ± 0.01
Miao	0.00E+00	1	5.27E-04	0.00E+00	1	5.27E-04	1.00E-03	0.152 ± 0.01	0.848 ± 0.01
Mongola	0.00E+00	1	7.20E-13	0.00E+00	1	7.20E-13	0.00E+00	0.14 ± 0.01	0.86 ± 0.01
Oroqen	0.00E+00	1	1.02E-20	0.00E+00	1	1.02E-20	0.00E+00	0.121 ± 0.011	0.879 ± 0.011
She	0.00E+00	1	1.30E-04	0.00E+00	1	1.30E-04	0.00E+00	0.167 ± 0.009	0.833 ± 0.099
Tu	0.00E+00	1	7.12E-18	0.00E+00	1	7.12E-18	0.00E+00	0.158 ± 0.01	0.842 ± 0.01
Tujia	0.00E+00	1	1.39E-02	0.00E+00	1	1.39E-02	1.40E-02	0.155 ± 0.01	0.845 ± 0.01
Uygur	0.00E+00	1	9.88E-324	0.00E+00	1	9.88E-324	0.00E+00	0.152 ± 0.014	0.848 ± 0.014
Xibo	0.00E+00	1	3.15E-06	0.00E+00	1	3.15E-06	0.00E+00	0.131 ± 0.01	0.869 ± 0.01
Cambodian	0.00E+00	1	1.34E-39	0.00E+00	1	1.34E-39	0.00E+00	0.144 ± 0.011	0.856 ± 0.011
Dusun	0.00E+00	1	7.29E-41	0.00E+00	1	7.29E-41	0.00E+00	0.162 ± 0.01	0.838 ± 0.01
Kinh	0.00E+00	1	5.41E-19	0.00E+00	1	5.41E-19	0.00E+00	0.17 ± 0.009	0.83 ± 0.009
Thai	0.00E+00	1	1.27E-31	0.00E+00	1	1.27E-31	0.00E+00	0.162 ± 0.01	0.838 ± 0.01
Yi	0.00E+00	1	9.90E-05	0.00E+00	1	9.90E-05	0.00E+00	0.151 ± 0.009	0.849 ± 0.009
Aleut	0.00E+00	1	1.07E-319	0.00E+00	1	1.07E-319	0.00E+00	0.148 ± 0.017	0.852 ± 0.017
Altaian	0.00E+00	1	6.57E-80	0.00E+00	1	6.57E-80	0.00E+00	0.121 ± 0.013	0.879 ± 0.013
Eskimo_Chaplin	0.00E+00	1	4.65E-67	0.00E+00	1	4.65E-67	0.00E+00	0.118 ± 0.014	0.882 ± 0.014
Eskimo_Naukan	0.00E+00	1	3.01E-129	0.00E+00	1	3.01E-129	0.00E+00	0.107 ± 0.013	0.893 ± 0.013
Eskimo_Sireniki	0.00E+00	1	4.25E-126	0.00E+00	1	4.25E-126	0.00E+00	0.121 ± 0.013	0.879 ± 0.013
Even	0.00E+00	1	7.24E-94	0.00E+00	1	7.24E-94	0.00E+00	0.115 ± 0.011	0.885 ± 0.011
Hezhen	0.00E+00	1	4.95E-12	0.00E+00	1	4.95E-12	0.00E+00	0.123 ± 0.01	0.877 ± 0.01
Itelman	0.00E+00	1	2.53E-78	0.00E+00	1	2.53E-78	0.00E+00	0.09 ± 0.014	0.91 ± 0.014
Tubalar	0.00E+00	1	4.30E-256	0.00E+00	1	4.30E-256	0.00E+00	0.135 ± 0.015	0.865 ± 0.015
Ulchi	0.00E+00	1	3.80E-17	0.00E+00	1	3.80E-17	0.00E+00	0.07 ± 0.013	0.93 ± 0.013
ARS008	0.00E+00	1	8.13E-75	0.00E+00	1	8.13E-75	0.00E+00	0.105 ± 0.012	0.895 ± 0.012
ARS026	0.00E+00	1	4.84E-262	0.00E+00	1	4.84E-262	0.00E+00	0.103 ± 0.016	0.897 ± 0.016
Lokomotiv_EN	0.00E+00	1	6.75E-53	0.00E+00	1	6.75E-53	0.00E+00	0.087 ± 0.01	0.913 ± 0.01
Shamanka_EBA	0.00E+00	1	6.10E-117	0.00E+00	1	6.10E-117	0.00E+00	0.105 ± 0.011	0.895 ± 0.011
Shamanka_EN	0.00E+00	1	2.83E-69	0.00E+00	1	2.83E-69	0.00E+00	0.111 ± 0.01	0.889 ± 0.01
Ust_Ida_EBA	0.00E+00	1	4.90E-143	0.00E+00	1	4.90E-143	0.00E+00	0.089 ± 0.011	0.911 ± 0.011
Ust_Ida_LN	0.00E+00	1	2.83E-158	0.00E+00	1	2.83E-158	0.00E+00	0.114 ± 0.012	0.886 ± 0.012

DevilsCave_N	0.00E+00	1	1.29E-10	0.00E+00	1	1.29E-10	0.00E+00	0.073 ± 0.01	0.927 ± 0.01
UstBelaya_N	0.00E+00	1	1.94E-23	0.00E+00	1	1.94E-23	0.00E+00	0.05 ± 0.008	0.95 ± 0.008
UstBelaya_EBA	0.00E+00	1	2.64E-123	0.00E+00	1	2.64E-123	0.00E+00	0.113 ± 0.011	0.887 ± 0.011
Ekven_IA	0.00E+00	1	8.14E-203	0.00E+00	1	8.14E-203	0.00E+00	0.126 ± 0.012	0.874 ± 0.012
Yana_MED	0.00E+00	1	2.01E-68	0.00E+00	1	2.01E-68	0.00E+00	0.08 ± 0.011	0.92 ± 0.011
McColl_Group2	0.00E+00	1	2.02E-50	0.00E+00	1	2.02E-50	0.00E+00	0.095 ± 0.01	0.905 ± 0.01
McColl_Group3	0.00E+00	1	3.49E-36	0.00E+00	1	3.49E-36	0.00E+00	0.067 ± 0.005	0.933 ± 0.005
McColl_Group4	0.00E+00	1	3.40E-21	0.00E+00	1	3.40E-21	0.00E+00	0.084 ± 0.007	0.916 ± 0.007
Boshan	0.00E+00	1	3.12E-04	0.00E+00	1	3.12E-04	0.00E+00	0.024 ± 0.003	0.976 ± 0.003
AR_EN	0.00E+00	1	1.31E-15	0.00E+00	1	1.31E-15	0.00E+00	0.053 ± 0.005	0.947 ± 0.005
AR_Xianbei_IA	0.00E+00	1	8.26E-18	0.00E+00	1	8.26E-18	0.00E+00	0.04 ± 0.005	0.96 ± 0.005
HMMH_MN	0.00E+00	1	1.60E-17	0.00E+00	1	1.60E-17	0.00E+00	0.071 ± 0.01	0.929 ± 0.01
WLR_MN	0.00E+00	1	2.78E-08	0.00E+00	1	2.78E-08	0.00E+00	0.112 ± 0.01	0.888 ± 0.01
WLR_LN	0.00E+00	1	2.46E-02	0.00E+00	1	2.46E-02	2.60E-02	0.05 ± 0.004	0.95 ± 0.004
WLR_BA	0.00E+00	1	1.41E-03	0.00E+00	1	1.41E-03	1.00E-03	0.08 ± 0.007	0.92 ± 0.007
WLR_BA_o	0.00E+00	1	5.26E-08	0.00E+00	1	5.26E-08	0.00E+00	0.079 ± 0.01	0.921 ± 0.01
Miaozigou_MN	0.00E+00	1	8.37E-09	0.00E+00	1	8.37E-09	0.00E+00	0.053 ± 0.005	0.947 ± 0.005
Shimao_LN	0.00E+00	1	2.21E-07	0.00E+00	1	2.21E-07	0.00E+00	0.114 ± 0.009	0.886 ± 0.009
Upper_YR_LN	0.00E+00	1	2.12E-21	0.00E+00	1	2.12E-21	0.00E+00	0.12 ± 0.008	0.88 ± 0.008
Upper_YR_IA	0.00E+00	1	1.43E-02	0.00E+00	1	1.43E-02	1.50E-02	0.115 ± 0.008	0.885 ± 0.008
YR_MN	0.00E+00	1	6.15E-08	0.00E+00	1	6.15E-08	0.00E+00	0.119 ± 0.008	0.881 ± 0.008
YR_LN	0.00E+00	1	2.80E-03	0.00E+00	1	2.80E-03	3.00E-03	0.127 ± 0.008	0.873 ± 0.008

Note: Source population is highlighted by bold if the admixture is supported from modelling with a given set of the reference populations or by bold and red if the admixture is supported from modelling with and without a subset of Jomon included in the reference populations.

Table S13. Testing fittings of three-way versus two-way admixture to the genetic ancestry of the Kofun by *qpAdm* with the third source represented by YR_LBIA.

**Left populations:**<br>**Target: JpIw**<br>**Sources:**<br>- **Jomon**<br>- **Northeast Asian ancestry (WLR_BA_o and HMMH_MN)**<br>- **YR_LBIA**<br><br>**Models**	**Degree of freedom**	**Tail probability**	x^2	***P*-value for the nested model (Three-way vs. Two-way models)**
**Right populations without a subset of Jomon**				
Three-way<br>(Jomon, Northeast Asian, and YR_LBIA)	5	7.83E-1	2.454	-
Two-way<br>(Jomon and YR_LBIA)	6	1.46E-1	9.520	7.86E-3
Two-way<br>(Northeast Asian and YR_LBIA)	6	1.45E-6	37.430	3.34E-9
Two-way<br>(Jomon and Northeast Asian)	6	2.13E-15	81.088	0.00E+00
**Right populations with a subset of Jomon**				
Three-way<br>(Jomon, Northeast Asian, and YR_LBIA)	6	2.34E-1	8.059	-
Two-way<br>(Jomon and YR_LBIA)	7	1.49E-1	10.764	1.00E-1
Two-way<br>(Northeast Asian and HYR_LBIAan)	7	4.10E-18	97.188	0.00E+00
Two-way<br>(Jomon and Northeast Asian)	7	1.55E-17	94.391	0.00E+00

Table S14. Testing fittings of two-way admixture to the genetic ancestry of the Yayoi and Kofun by *qpAdm*.

Left populations:<br>Target: Yayoi or JpIw<br>Sources:<br>- Jomon<br>- YR_LBIA	*qpWave*						*qpAdm*		
	Rank 0 (Single ancestor)		Rank 1 (Two ancestors)		Rank 2 (Full model)		Tail probability	Admixture proportions (± 1 standard error)	
	Tail	Tail-diff	Tail	Tail-diff	Tail	Tail-diff		Jomon	YR_LBIA
**Right populations with a subset of Jomon**									
Yayoi	0	1	3.88E-01	0	1	3.88E-01	3.89E-01	0.626 ± 0.019	0.374 ± 0.019
JpIw	0	1	1.51E-01	0	1	1.51E-01	1.54E-01	0.125 ± 0.008	0.875 ± 0.008

Table S15. Testing fittings of three-way versus four-way admixture to the genetic ancestry of the Kofun by *qpAdm*.

Left populations:<br>Target: JpIw<br>Sources:<br>- Jomon<br>- Northeast Asian ancestry (WLR_BA_o + HMMH_MN)<br>- East Asian ancestry (Han)<br>- Additional source	Three-way admixture (Jomon, Northeast Asian, and East Asian ancestry)		Four-way admixture		*P*-value for nested model
**Additional source**	χ^2-value	Degree of freedom	χ^2-value	Degree of freedom	
**Right populations without a subset of Jomon**					
ARS026	5.182	5	4.936	4	0.620
Shamanka_EBA	5.197	5	3.681	4	0.218
Shamanka_EN	5.181	5	4.693	4	0.485
Ust_Ida_EBA	5.166	5	4.130	4	0.309
Namazga_CA	5.263	5	5.257	4	0.938
Okunevo_EMBA	5.245	5	4.713	4	0.466
UstBelaya_EBA	5.192	5	4.619	4	0.449
Yana_MED	5.145	5	4.807	4	0.561
McColl_Group2	5.179	5	3.216	4	0.161
McColl_Group3	5.176	5	5.191	4	1.000
Miaozigou_MN	5.164	5	3.470	4	0.193
Upper_YR_LN	5.179	5	3.911	4	0.260
YR_MN	5.205	5	2.177	4	0.082
YR_LN	5.170	5	5.216	4	1.000
YR_LBIA	5.184	5	2.416	4	0.096
**Right populations with a subset of Jomon**					
ARS026	5.527	6	5.470	5	0.811
Shamanka_EBA	5.690	6	4.852	5	0.360
Shamanka_EN*	32.111<br>(5.723)	6<br>(6)	5.843	5	0.000<br>(1.000)
Ust_Ida_EBA	5.589	6	4.985	5	0.437
Namazga_CA*	101.784<br>(9.735)	6<br>(6)	5.925	5	0.000<br>(0.051)
Okunevo_EMBA*	97.024<br>(8.482)	6<br>(6)	5.598	5	0.000<br>(0.089)
UstBelaya_EBA	6.170	6	5.419	5	0.386
Yana_MED	5.612	6	5.495	5	0.732
McColl_Group2	6.328	6	6.164	5	0.686

McColl_Group3	6.391	6	6.524	5	1.000
Miaozigou_MN	6.315	6	6.401	5	1.000
Upper_YR_LN	9.218	6	5.735	5	0.062
YR_MN	7.132	6	5.361	5	0.183
YR_LN	7.588	6	6.653	5	0.334
YR_LBIA	8.747	6	7.590	5	0.282

*These populations have an alternative model of three-way admixture shown in the parentheses that includes Jomon, East Asian ancestry, and additional source tested, instead of Jomon, Northeast Asian, and East Asian ancestry, likely due to their shared ancestry with the source population representing Northeast Asian ancestry (WLR_BA_o + HMMH_MN).

Table 16. Modelling on the genetic ancestry of modern Japanese only by Kofun ancestry (no admixture) or by a mixture of two sources by *qpAdm*.

**Left populations:**<br>**Target: Japanese**<br>**Sources:**<br>- **JpIw**<br>- **Additional source**	**No admixture (JpIw)**		**Two-way admixture (JpIw and additional source)**		***P*-value for nested model**
**Additional source**	**χ^2-value**	**Degree of freedom**	**χ^2-value**	**Degree of freedom**	
**Right populations without a subset of Jomon**					
Yayoi	7	3.929	6	2.670	0.262
Jomon	7	3.914	6	3.235	0.410
Han	7	3.922	6	2.577	0.246
Tujia	7	3.917	6	3.464	0.501
Atayal	7	3.901	6	3.895	0.938
Daur	7	3.903	6	2.627	0.259
Korean	7	3.918	6	3.428	0.484
Lahu	7	3.913	6	3.723	0.663
Miao	7	3.918	6	2.718	0.273
Mongola	7	3.916	6	2.858	0.304
Oroqen	7	3.915	6	2.125	0.181
She	7	3.916	6	3.206	0.399
Tu	7	3.918	6	2.420	0.221
Xibo	7	3.917	6	2.589	0.249
Burmese	7	3.903	6	2.381	0.217
Cambodian	7	3.914	6	3.799	0.735
Dusun	7	3.914	6	3.810	0.747
Kinh	7	3.918	6	3.697	0.638
Thai	7	3.917	6	3.930	1.000
Yi	7	3.917	6	3.872	0.832
Aleut	7	3.912	6	2.183	0.189
Altaian	7	3.905	6	2.272	0.201
Chukchi	7	3.902	6	2.390	0.219
Eskimo_Chaplin	7	3.902	6	2.155	0.186
Eskimo_Naukan	7	3.913	6	2.267	0.200
Eskimo_Sireniki	7	3.912	6	2.177	0.188
Even	7	3.920	6	2.460	0.227
Hezhen	7	3.917	6	2.999	0.338

Itelman	7	3.905	6	1.943	0.161
Mansi	7	3.910	6	2.276	0.201
Russian	7	3.905	6	2.479	0.232
Tubalar	7	3.911	6	2.344	0.211
Ulchi	7	3.915	6	2.016	0.168
**Right populations with a subset of Jomon**					
Yayoi	8	4.404	7	3.234	0.279
Jomon	8	4.397	7	3.599	0.372
Han	8	4.397	7	3.126	0.260
Tujia	8	4.390	7	3.548	0.359
Atayal	8	4.371	7	4.154	0.641
Daur	8	4.373	7	4.392	1.000
Korean	8	4.392	7	3.538	0.355
Lahu	8	4.385	7	3.752	0.426
Miao	8	4.392	7	3.165	0.268
Mongola	8	4.389	7	4.391	1.000
Oroqen	8	4.388	7	4.261	0.722
She	8	4.390	7	3.354	0.309
Tu	8	4.391	7	4.405	1.000
Xibo	8	4.391	7	4.383	0.929
Burmese	8	4.372	7	4.351	0.885
Cambodian	8	4.386	7	4.325	0.805
Dusun	8	4.386	7	3.889	0.481
Kinh	8	4.392	7	3.692	0.403
Thai	8	4.390	7	4.062	0.567
Yi	8	4.391	7	3.868	0.470
Aleut	8	4.384	7	3.413	0.324
Altaian	8	4.375	7	3.719	0.418
Chukchi	8	4.371	7	3.484	0.346
Eskimo_Chaplin	8	4.372	7	3.724	0.421
Eskimo_Naukan	8	4.386	7	3.707	0.410
Eskimo_Sireniki	8	4.384	7	3.628	0.385
Even	8	4.395	7	4.034	0.548
Hezhen	8	4.391	7	4.393	1.000
Itelman	8	4.375	7	3.509	0.352

Mansi	8	4.382	7	3.432	0.330
Russian	8	4.374	7	3.442	0.334
Tubalar	8	4.384	7	3.527	0.355
Ulchi	8	4.387	7	3.836	0.458

Table S17. Modelling on the genetic ancestry of present-day Japanese as a mixture of three sources by *qpWave* and *qpAdm*.

Left populations:<br>- Japanese<br>- Jomon<br>- Northeast Asian ancestry (WLR_BA_o + HMMH_MN)<br>- East Asian ancestry (Han)	*qpWave*					*qpAdm* (Target: Japanese)			
	Probability	Rank				Tail	Admixture proportions (± 1 standard error)		
		0	1	2	3		Jomon	Northeast Asian ancestry	East Asian ancestry
**Right populations without a subset of Jomon**	**Tail**	**1.69E-154**	**7.15E-19**	**7.68E-01**	**1**	**7.71E-01**	**0.131 ± 0.035**	**0.156 ± 0.077**	**0.713 ± 0.061**
	**Tail-diff**	**1**	**1.90E-140**	**3.36E-21**	**7.68E-01**				
**Right populations with a subset of Jomon**	**Tail**	**0**	**3.047E-26**	**8.370E-01**	**1**	**8.40E-01**	**0.128 ± 0.009**	**0.163 ± 0.065**	**0.709 ± 0.061**
	**Tail-diff**	**1**	**0**	**2.761E-29**	**8.370E-01**				

Note: Source population is highlighted by bold if the admixture is supported from modelling with a given set of the reference populations or by bold and red if the admixture is supported from modelling with and without a subset of Jomon included in the reference populations.

Table S18. Test of the genetic continuity between Kofun and present-day Japanese.

Model	Drift parameter		Log-likelihood
	t_1 (JPT)	t_2 (Kofun)	
Continuity	0.009	- (fixed as $t_2 = 0$)	-3,697,125.918
Non-continuity	0.004	0.015	-3,696,423.430

REFERENCES AND NOTES

1. K. Mizoguchi, *The Archaeology of Japan: From the Earliest Rice Farming Villages to the Rise of the State* (Cambridge Univ. Press, 2013).

2. J. Habu, *Ancient Jomon of Japan* (Cambridge Univ. Press, 2004).

3. P. U. Clark, J. D. Shakun, P. A. Baker, P. J. Bartlein, S. Brewer, E. Brook, A. E. Carlson, H. Cheng, D. S. Kaufman, Z. Liu, T. M. Marchitto, A. C. Mix, C. Morrill, B. L. Otto-Bliesner, K. Pahnke, J. M. Russell, C. Whitlock, J. F. Adkins, J. L. Blois, J. Clark, S. M. Colman, W. B. Curry, B. P. Flower, F. He, T. C. Johnson, J. Lynch-Stieglitz, V. Markgraf, J. McManus, J. X. Mitrovica, P. I. Moreno, J. W. Williams, Global climate evolution during the last deglaciation. *Proc. Natl. Acad. Sci. U.S.A.* **109**, E1134–E1142 (2012).

4. E. R. Crema, J. Habu, K. Kobayashi, M. Madella, Summed probability distribution of ^{14}C dates suggests regional divergences in the population dynamics of the Jomon period in Eastern Japan. *PLOS ONE* **11**, e0154809 (2016).

5. K. Hanihara, Dual structure model for the population history of the Japanese. *Japan Review* **2**, 1–33 (1991).

6. M. J. Hudson, S. Nakagome, J. B. Whitman, The evolving Japanese: The dual structure hypothesis at 30. *Evol. Hum. Sci.* **2**, E6 (2020).

7. Japanese Archipelago Human Population Genetics Consortium, T. Jinam, N. Nishida, M. Hirai, S. Kawamura, H. Oota, K. Umetsu, R. Kimura, J. Ohashi, A. Tajima, T. Yamamoto, H. Tanabe, S. Mano, Y. Suto, T. Kaname, K. Naritomi, K. Yanagi, N. Niikawa, K. Omoto, K. Tokunaga, N. Saitou, The history of human populations in the Japanese Archipelago inferred from genome-wide SNP data with a special reference to the Ainu and the Ryukyuan populations. *J. Hum. Genet.* **57**, 787–795 (2012).

8. S. Nakagome, T. Sato, H. Ishida, T. Hanihara, T. Yamaguchi, R. Kimura, S. Mano, H. Oota; Asian DNA Repository Consortium, Model-based verification of hypotheses on the origin of modern Japanese revisited by Bayesian inference based on genome-wide SNP data. *Mol. Biol. Evol.* **32**,

1533–1543 (2015).

9. T. Jinam, Y. Kawai, Y. Kamatani, S. Sonoda, K. Makisumi, H. Sameshima, K. Tokunaga, N. Saitou, Genome-wide SNP data of Izumo and Makurazaki populations support inner-dual structure model for origin of Yamato people. *J. Hum. Genet.* **66**, 681–687 (2021).

10. T. A. Jinam, Y. Kawai, N. Saitou, Modern human DNA analyses with special reference to the inner dual-structure model of Yaponesian. *Anthropol. Sci.* **129**, 3–11 (2021).

11. H. Kanzawa-Kiriyama, K. Kryukov, T. A. Jinam, K. Hosomichi, A. Saso, G. Suwa, S. Ueda, M. Yoneda, A. Tajima, K.-I. Shinoda, I. Inoue, N. Saitou, A partial nuclear genome of the Jomons who lived 3000 years ago in Fukushima, Japan. *J. Hum. Genet.* **62**, 213–221 (2017).

12. H. McColl, F. Racimo, L. Vinner, F. Demeter, T. Gakuhari, J. V. Moreno-Mayar, G. van Driem, U. G. Wilken, A. Seguin-Orlando, C. de la Fuente Castro, S. Wasef, R. Shoocongdej, V. Souksavatdy, T. Sayavongkhamdy, M. M. Saidin, M. E. Allentoft, T. Sato, A.-S. Malaspinas, F. A. Aghakhanian, T. Korneliussen, A. Prohaska, A. Margaryan, P. de Barros Damgaard, S. Kaewsutthi, P. Lertrit, T. M. H. Nguyen, H.-c. Hung, T. M. Tran, H. N. Truong, G. H. Nguyen, S. Shahidan, K. Wiradnyana, H. Matsumae, N. Shigehara, M. Yoneda, H. Ishida, T. Masuyama, Y. Yamada, A. Tajima, H. Shibata, A. Toyoda, T. Hanihara, S. Nakagome, T. Deviese, A.-M. Bacon, P. Duringer, J.-L. Ponche, L. Shackelford, E. Patole-Edoumba, A. T. Nguyen, B. Bellina-Pryce, J.-C. Galipaud, R. Kinaston, H. Buckley, C. Pottier, S. Rasmussen, T. Higham, R. A. Foley, M. M. Lahr, L. Orlando, M. Sikora, M. E. Phipps, H. Oota, C. Higham, D. M. Lambert, E. Willerslev, The prehistoric peopling of Southeast Asia. *Science* **361**, 88–92 (2018).

13. T. Gakuhari, S. Nakagome, S. Rasmussen, M. E. Allentoft, T. Sato, T. Korneliussen, B. N. Chuinneagáin, H. Matsumae, K. Koganebuchi, R. Schmidt, S. Mizushima, O. Kondo, N. Shigehara, M. Yoneda, R. Kimura, H. Ishida, T. Masuyama, Y. Yamada, A. Tajima, H. Shibata, A. Toyoda, T. Tsurumoto, T. Wakebe, H. Shitara, T. Hanihara, E. Willerslev, M. Sikora, H. Oota, Ancient Jomon genome sequence analysis sheds light on migration patterns of early East Asian populations. *Commun. Biol.* **3**, 437 (2020).

14. H. Kanzawa-Kiriyama, T. A. Jinam, Y. Kawai, T. Sato, K. Hosomichi, A. Tajima, N. Adachi, H.

Matsumura, K. Kryukov, N. Saitou, K.-I. Shinoda, Late Jomon male and female genome sequences from the Funadomari site in Hokkaido, Japan. *Anthropol. Sci.* **127**, 83–108 (2019).

15. K.-i. Shinoda, H. Kanzawa-Kiriyama, T. Kakuda, N. Adachi, Genetic characteristics of Yayoi people in Northwestern Kyushu. *Anthropol. Sci. (Japanese Series)* **127**, 25–43 (2019).

16. Y. Kaifu, K. Sakaue, R. T. Kono, Early Jomon and Yayoi human skeletal remains from Shimomotoyama Rock Shelter, Sasebo, Nagasaki prefecture, Japan. *Anthropol. Sci. (Japanese Series)* **125**, 25–38 (2017).

17. P. de Barros Damgaard, R. Martiniano, J. Kamm, J. V. Moreno-Mayar, G. Kroonen, M. Peyrot, G. Barjamovic, S. Rasmussen, C. Zacho, N. Baimukhanov, V. Zaibert, V. Merz, A. Biddanda, I. Merz, V. Loman, V. Evdokimov, E. Usmanova, B. Hemphill, A. Seguin-Orlando, F. E. Yediay, I. Ullah, K.-G. Sjögren, K. H. Iversen, J. Choin, C. de la Fuente, M. Ilardo, H. Schroeder, V. Moiseyev, A. Gromov, A. Polyakov, S. Omura, S. Y. Senyurt, H. Ahmad, C. McKenzie, A. Margaryan, A. Hameed, A. Samad, N. Gul, M. H. Khokhar, O. I. Goriunova, V. I. Bazaliiskii, J. Novembre, A. W. Weber, L. Orlando, M. E. Allentoft, R. Nielsen, K. Kristiansen, M. Sikora, A. K. Outram, R. Durbin, E. Willerslev, The first horse herders and the impact of early Bronze Age steppe expansions into Asia. *Science* **360**, eaar7711 (2018).

18. C. Jeong, S. Wilkin, T. Amgalantugs, A. S. Bouwman, W. T. T. Taylor, R. W. Hagan, S. Bromage, S. Tsolmon, C. Trachsel, J. Grossmann, J. Littleton, C. A. Makarewicz, J. Krigbaum, M. Burri, A. Scott, G. Davaasambuu, J. Wright, F. Irmer, E. Myagmar, N. Boivin, M. Robbeets, F. J. Rühli, J. Krause, B. Frohlich, J. Hendy, C. Warinner, Bronze Age population dynamics and the rise of dairy pastoralism on the eastern Eurasian steppe. *Proc. Natl. Acad. Sci. U.S.A.* **115**, E11248–E11255 (2018).

19. M. Sikora, V. V. Pitulko, V. C. Sousa, M. E. Allentoft, L. Vinner, S. Rasmussen, A. Margaryan, P. de Barros Damgaard, C. de la Fuente, G. Renaud, M. A. Yang, Q. Fu, I. Dupanloup, K. Giampoudakis, D. Nogués-Bravo, C. Rahbek, G. Kroonen, M. Peyrot, H. McColl, S. V. Vasilyev, E. Veselovskaya, M. Gerasimova, E. Y. Pavlova, V. G. Chasnyk, P. A. Nikolskiy, A. V. Gromov, V. I. Khartanovich, V. Moiseyev, P. S. Grebenyuk, A. Y. Fedorchenko, A. I. Lebedintsev, S. B. Slobodin, B. A. Malyarchuk, R. Martiniano, M. Meldgaard, L. Arppe, J. U. Palo, T. Sundell, K. Mannermaa, M.

Putkonen, V. Alexandersen, C. Primeau, N. Baimukhanov, R. S. Malhi, K.-G. Sjögren, K. Kristiansen, A. Wessman, A. Sajantila, M. M. Lahr, R. Durbin, R. Nielsen, D. J. Meltzer, L. Excoffier, E. Willerslev, The population history of northeastern Siberia since the Pleistocene. *Nature* **570**, 182–188 (2019).

20. C. Ning, T. Li, K. Wang, F. Zhang, T. Li, X. Wu, S. Gao, Q. Zhang, H. Zhang, M. J. Hudson, G. Dong, S. Wu, Y. Fang, C. Liu, C. Feng, W. Li, T. Han, R. Li, J. Wei, Y. Zhu, Y. Zhou, C.-C. Wang, S. Fan, Z. Xiong, Z. Sun, M. Ye, L. Sun, X. Wu, F. Liang, Y. Cao, X. Wei, H. Zhu, H. Zhou, J. Krause, M. Robbeets, C. Jeong, Y. Cui, Ancient genomes from northern China suggest links between subsistence changes and human migration. *Nat. Commun.* **11**, 2700 (2020).

21. M. A. Yang, X. Fan, B. Sun, C. Chen, J. Lang, Y.-C. Ko, C.-H. Tsang, H. Chiu, T. Wang, Q. Bao, X. Wu, M. Hajdinjak, A. M.-S. Ko, M. Ding, P. Cao, R. Yang, F. Liu, B. Nickel, Q. Dai, X. Feng, L. Zhang, C. Sun, C. Ning, W. Zeng, Y. Zhao, M. Zhang, X. Gao, Y. Cui, D. Reich, M. Stoneking, Q. Fu, Ancient DNA indicates human population shifts and admixture in northern and southern China. *Science* **369**, 282–288 (2020).

22. N. Adachi, K.-I. Shinoda, K. Umetsu, T. Kitano, H. Matsumura, R. Fujiyama, J. Sawada, M. Tanaka, Mitochondrial DNA analysis of Hokkaido Jomon skeletons: Remnants of archaic maternal lineages at the southwestern edge of former Beringia. *Am. J. Phys. Anthropol.* **146**, 346–360 (2011).

23. M. Tanaka, V. M. Cabrera, A. M. González, J. M. Larruga, T. Takeyasu, N. Fuku, L.-J. Guo, R. Hirose, Y. Fujita, M. Kurata, K.-I. Shinoda, K. Umetsu, Y. Yamada, Y. Oshida, Y. Sato, N. Hattori, Y. Mizuno, Y. Arai, N. Hirose, S. Ohta, O. Ogawa, Y. Tanaka, R. Kawamori, M. Shamoto-Nagai, W. Maruyama, H. Shimokata, R. Suzuki, H. Shimodaira, Mitochondrial genome variation in eastern Asia and the peopling of Japan. *Genome Res.* **14**, 1832–1850 (2004).

24. M. F. Hammer, T. M. Karafet, H. Park, K. Omoto, S. Harihara, M. Stoneking, S. Horai, Dual origins of the Japanese: Common ground for hunter-gatherer and farmer Y chromosomes. *J. Hum. Genet.* **51**, 47–58 (2006).

25. H.-X. Zheng, S. Yan, Z.-D. Qin, Y. Wang, J.-Z. Tan, H. Li, L. Jin, Major population expansion of East Asians began before neolithic time: Evidence of mtDNA genomes. *PLOS ONE* **6**, e25835

(2011).

26. C.-C. Wang, H. Li, Inferring human history in East Asia from Y chromosomes. *Investigative Genet.* **4**, 11 (2013).

27. S. Mallick, H. Li, M. Lipson, I. Mathieson, M. Gymrek, F. Racimo, M. Zhao, N. Chennagiri, S. Nordenfelt, A. Tandon, P. Skoglund, I. Lazaridis, S. Sankararaman, Q. Fu, N. Rohland, G. Renaud, Y. Erlich, T. Willems, C. Gallo, J. P. Spence, Y. S. Song, G. Poletti, F. Balloux, G. van Driem, P. de Knijff, I. G. Romero, A. R. Jha, D. M. Behar, C. M. Bravi, C. Capelli, T. Hervig, A. Moreno-Estrada, O. L. Posukh, E. Balanovska, O. Balanovsky, S. Karachanak-Yankova, H. Sahakyan, D. Toncheva, L. Yepiskoposyan, C. Tyler-Smith, Y. Xue, M. S. Abdullah, A. Ruiz-Linares, C. M. Beall, A. Di Rienzo, C. Jeong, E. B. Starikovskaya, E. Metspalu, J. Parik, R. Villems, B. M. Henn, U. Hodoglugil, R. Mahley, A. Sajantila, G. Stamatoyannopoulos, J. T. S. Wee, R. Khusainova, E. Khusnutdinova, S. Litvinov, G. Ayodo, D. Comas, M. F. Hammer, T. Kivisild, W. Klitz, C. A. Winkler, D. Labuda, M. Bamshad, L. B. Jorde, S. A. Tishkoff, W. S. Watkins, M. Metspalu, S. Dryomov, R. Sukernik, L. Singh, K. Thangaraj, S. Pääbo, J. Kelso, N. Patterson, D. Reich, The Simons Genome Diversity Project: 300 genomes from 142 diverse populations. *Nature* **538**, 201–206 (2016).

28. 1000 Genomes Project Consortium, A. Auton, L. D. Brooks, R. M. Durbin, E. P. Garrison, H. M. Kang, J. O. Korbel, J. L. Marchini, S. McCarthy, G. A. McVean, G. R. Abecasis, A global reference for human genetic variation. *Nature* **526**, 68–74 (2015).

29. J. K. Pickrell, J. K. Pritchard, Inference of population splits and mixtures from genome-wide allele frequency data. *PLOS Genet.* **8**, e1002967 (2012).

30. C.-C. Wang, H.-Y. Yeh, A. N. Popov, H.-Q. Zhang, H. Matsumura, K. Sirak, O. Cheronet, A. Kovalev, N. Rohland, A. M. Kim, S. Mallick, R. Bernardos, D. Tumen, J. Zhao, Y.-C. Liu, J.-Y. Liu, M. Mah, K. Wang, Z. Zhang, N. Adamski, N. Broomandkhoshbacht, K. Callan, F. Candilio, K. S. D. Carlson, B. J. Culleton, L. Eccles, S. Freilich, D. Keating, A. M. Lawson, K. Mandl, M. Michel, J. Oppenheimer, K. T. Özdoğan, K. Stewardson, S. Wen, S. Yan, F. Zalzala, R. Chuang, C.-J. Huang, H. Looh, C.-C. Shiung, Y. G. Nikitin, A. V. Tabarev, A. A. Tishkin, S. Lin, Z.-Y. Sun, X.-M. Wu, T.-L. Yang, X. Hu, L. Chen, H. Du, J. Bayarsaikhan, E. Mijiddorj, D. Erdenebaatar, T.-O. Iderkhangai, E. Myagmar, H. Kanzawa-Kiriyama, M. Nishino, K.-I. Shinoda, O. A. Shubina, J. Guo, W. Cai, Q.

Deng, L. Kang, D. Li, D. Li, R. Lin, Nini, R. Shrestha, L.-X. Wang, L. Wei, G. Xie, H. Yao, M. Zhang, G. He, X. Yang, R. Hu, M. Robbeets, S. Schiffels, D. J. Kennett, L. Jin, H. Li, J. Krause, R. Pinhasi, D. Reich, Genomic insights into the formation of human populations in East Asia. *Nature* **591**, 413–419 (2021).

31. F. C. Ceballos, P. K. Joshi, D. W. Clark, M. Ramsay, J. F. Wilson, Runs of homozygosity: Windows into population history and trait architecture. *Nat. Rev. Genet.* **19**, 220–234 (2018).

32. D. M. Fernandes, K. A. Sirak, H. Ringbauer, J. Sedig, N. Rohland, O. Cheronet, M. Mah, S. Mallick, I. Olalde, B. J. Culleton, N. Adamski, R. Bernardos, G. Bravo, N. Broomandkhoshbacht, K. Callan, F. Candilio, L. Demetz, K. S. D. Carlson, L. Eccles, S. Freilich, R. J. George, A. M. Lawson, K. Mandl, F. Marzaioli, W. C. McCool, J. Oppenheimer, K. T. Özdogan, C. Schattke, R. Schmidt, K. Stewardson, F. Terrasi, F. Zalzala, C. A. Antúnez, E. V. Canosa, R. Colten, A. Cucina, F. Genchi, C. Kraan, F. La Pastina, M. Lucci, M. V. Maggiolo, B. Marcheco-Teruel, C. T. Maria, C. Martínez, I. París, M. Pateman, T. M. Simms, C. G. Sivoli, M. Vilar, D. J. Kennett, W. F. Keegan, A. Coppa, M. Lipson, R. Pinhasi, D. Reich, A genetic history of the pre-contact Caribbean. *Nature* **590**, 103–110 (2021).

33. L. M. Cassidy, R. Ó. Maoldúin, T. Kador, A. Lynch, C. Jones, P. C. Woodman, E. Murphy, G. Ramsey, M. Dowd, A. Noonan, C. Campbell, E. R. Jones, V. Mattiangeli, D. G. Bradley, A dynastic elite in monumental Neolithic society. *Nature* **582**, 384–388 (2020).

34. J. d'Alpoim Guedes, J. Austermann, J. X. Mitrovica, Lost foraging opportunities for East Asian hunter-gatherers due to rising sea level since the Last Glacial Maximum. *Geoarchaeology* **31**, 255–266 (2016).

35. K. Lambeck, H. Rouby, A. Purcell, Y. Sun, M. Sambridge, Sea level and global ice volumes from the Last Glacial Maximum to the Holocene. *Proc. Natl. Acad. Sci. U.S.A.* **111**, 15296–15303 (2014).

36. D. Q. Fuller, L. Qin, Y. Zheng, Z. Zhao, X. Chen, L. A. Hosoya, G.-P. Sun, The domestication process and domestication rate in rice: Spikelet bases from the Lower Yangtze. *Science* **323**, 1607–1610 (2009).

37. P. Skoglund, H. Malmström, A. Omrak, M. Raghavan, C. Valdiosera, T. Günther, P. Hall, K. Tambets, J. Parik, K.-G. Sjögren, J. Apel, E. Willerslev, J. Storå, A. Götherström, M. Jakobsson, Genomic diversity and admixture differs for Stone-Age Scandinavian foragers and farmers. *Science* **344**, 747–750 (2014).

38. C. Gamba, E. R. Jones, M. D. Teasdale, R. L. McLaughlin, G. Gonzalez-Fortes, V. Mattiangeli, L. Domboróczki, I. Kővári, I. Pap, A. Anders, A. Whittle, J. Dani, P. Raczky, T. F. G. Higham, M. Hofreiter, D. G. Bradley, R. Pinhasi, Genome flux and stasis in a five millennium transect of European prehistory. *Nat. Commun.* **5**, 5257 (2014).

39. I. Lazaridis, N. Patterson, A. Mittnik, G. Renaud, S. Mallick, K. Kirsanow, P. H. Sudmant, J. G. Schraiber, S. Castellano, M. Lipson, B. Berger, C. Economou, R. Bollongino, Q. Fu, K. I. Bos, S. Nordenfelt, H. Li, C. de Filippo, K. Prüfer, S. Sawyer, C. Posth, W. Haak, F. Hallgren, E. Fornander, N. Rohland, D. Delsate, M. Francken, J.-M. Guinet, J. Wahl, G. Ayodo, H. A. Babiker, G. Bailliet, E. Balanovska, O. Balanovsky, R. Barrantes, G. Bedoya, H. Ben-Ami, J. Bene, F. Berrada, C. M. Bravi, F. Brisighelli, G. B. J. Busby, F. Cali, M. Churnosov, D. E. C. Cole, D. Corach, L. Damba, G. van Driem, S. Dryomov, J.-M. Dugoujon, S. A. Fedorova, I. G. Romero, M. Gubina, M. Hammer, B. M. Henn, T. Hervig, U. Hodoglugil, A. R. Jha, S. Karachanak-Yankova, R. Khusainova, E. Khusnutdinova, R. Kittles, T. Kivisild, W. Klitz, V. Kučinskas, A. Kushniarevich, L. Laredj, S. Litvinov, T. Loukidis, R. W. Mahley, B. Melegh, E. Metspalu, J. Molina, J. Mountain, K. Näkkäläjärvi, D. Nesheva, T. Nyambo, L. Osipova, J. Parik, F. Platonov, O. Posukh, V. Romano, F. Rothhammer, I. Rudan, R. Ruizbakiev, H. Sahakyan, A. Sajantila, A. Salas, E. B. Starikovskaya, A. Tarekegn, D. Toncheva, S. Turdikulova, I. Uktveryte, O. Utevska, R. Vasquez, M. Villena, M. Voevoda, C. A. Winkler, L. Yepiskoposyan, P. Zalloua, T. Zemunik, A. Cooper, C. Capelli, M. G. Thomas, A. Ruiz-Linares, S. A. Tishkoff, L. Singh, K. Thangaraj, R. Villems, D. Comas, R. Sukernik, M. Metspalu, M. Meyer, E. E. Eichler, J. Burger, M. Slatkin, S. Pääbo, J. Kelso, D. Reich, J. Krause, Ancient human genomes suggest three ancestral populations for present-day Europeans. *Nature* **513**, 409–413 (2014).

40. L. M. Cassidy, R. Martiniano, E. M. Murphy, M. D. Teasdale, J. Mallory, B. Hartwell, D. G. Bradley, Neolithic and Bronze Age migration to Ireland and establishment of the insular Atlantic genome. *Proc. Natl. Acad. Sci. U.S.A.* **113**, 368–373 (2016).

41. K. Miyamoto, The spread of rice agriculture during the Yayoi period: From the Shandong Peninsula to Japanese Archipelago via Korean Peninsula. *Jpn. J. Archeol.* **6**, 109–124 (2019).

42. H. Nasu, A. Momohara, The beginnings of rice and millet agriculture in prehistoric Japan. *Quat. Int.* **397**, 504–512 (2016).

43. H. Matsumura, A microevolutional history of the Japanese people from a dental characteristics perspective. *Anthropol. Sci.* **102**, 93–118 (1994).

44. Y. Kitagawa, Nonmetric morphological characters of deciduous teeth in Japan: Diachronic evidence of the past 4000 years. *Int. J. Osteoarchaeol.* **10**, 242–253 (2000).

45. V. M. Narasimhan, N. Patterson, P. Moorjani, N. Rohland, R. Bernardos, S. Mallick, I. Lazaridis, N. Nakatsuka, I. Olalde, M. Lipson, A. M. Kim, L. M. Olivieri, A. Coppa, M. Vidale, J. Mallory, V. Moiseyev, E. Kitov, J. Monge, N. Adamski, N. Alex, N. Broomandkhoshbacht, F. Candilio, K. Callan, O. Cheronet, B. J. Culleton, M. Ferry, D. Fernandes, S. Freilich, B. Gamarra, D. Gaudio, M. Hajdinjak, É. Harney, T. K. Harper, D. Keating, A. M. Lawson, M. Mah, K. Mandl, M. Michel, M. Novak, J. Oppenheimer, N. Rai, K. Sirak, V. Slon, K. Stewardson, F. Zalzala, Z. Zhang, G. Akhatov, A. N. Bagashev, A. Bagnera, B. Baitanayev, J. Bendezu-Sarmiento, A. A. Bissembaev, G. L. Bonora, T. T. Chargynov, T. Chikisheva, P. K. Dashkovskiy, A. Derevianko, M. Dobeš, K. Douka, N. Dubova, M. N. Duisengali, D. Enshin, A. Epimakhov, A. V. Fribus, D. Fuller, A. Goryachev, A. Gromov, S. P. Grushin, B. Hanks, M. Judd, E. Kazizov, A. Khokhlov, A. P. Krygin, E. Kupriyanova, P. Kuznetsov, D. Luiselli, F. Maksudov, A. M. Mamedov, T. B. Mamirov, C. Meiklejohn, D. C. Merrett, R. Micheli, O. Mochalov, S. Mustafokulov, A. Nayak, D. Pettener, R. Potts, D. Razhev, M. Rykun, S. Sarno, T. M. Savenkova, K. Sikhymbaeva, S. M. Slepchenko, O. A. Soltobaev, N. Stepanova, S. Svyatko, K. Tabaldiev, M. Teschler-Nicola, A. A. Tishkin, V. V. Tkachev, S. Vasilyev, P. Velemínský, D. Voyakin, A. Yermolayeva, M. Zahir, V. S. Zubkov, A. Zubova, V. S. Shinde, C. Lalueza-Fox, M. Meyer, D. Anthony, N. Boivin, K. Thangaraj, D. J. Kennett, M. Frachetti, R. Pinhasi, D. Reich, The formation of human populations in South and Central Asia. *Science* **365**, eaat7487 (2019).

46. J. G. Schraiber, Assessing the relationship of ancient and modern populations. *Genetics* **208**, 383–398 (2018).

47. Y. Dodo, Y. Kawakubo, Cranial affinities of the Epi-Jomon inhabitants in Hokkaido, Japan. *Anthropol. Sci.* **110**, 1–32 (2002).

48. H. Matsumura, Geographical variation of dental characteristics in the Japanese of the protohistoric Kofun period. *Anthropol. Sci.* **98**, 439–449 (1990).

49. Y. Mizoguchi, Affinities of the protohistoric Kofun people of Japan with pre- and proto-historic asian populations. *Anthropol. Sci.* **96**, 71–109 (1988).

50. J. Habu, Early sedentism in East Asia: From Late Palaeolithic to early agricultural societies in insular East Asia, in *The Cambridge World Prehistory* (Cambridge Univ. Press, 2014), vol. 3, pp. 724–741.

51. N. Matsumoto, J. Habu, A. Matsui, in *Handbook of East and Southeast Asian Archaeology*, J. Habu, P. V. Lape, J. W. Olsen, Eds. (Springer New York, 2017), pp. 437–450.

52. K. Imamura, *Prehistoric Japan: New Perspectives on Insular East Asia* (University of Hawaii Press, 1996).

53. R. Pearson, Debating Jomon social complexity. *Asian Perspect.* **46**, 361–388 (2007).

54. M. Robbeets, Proto-transeurasian: Where and when? *Man in India: Int. J. Anthropol.* **97**, 19–46 (2017).

55. D. Q. Fuller, L. Qin, Water management and labour in the origins and dispersal of Asian rice. *World Archaeol.* **41**, 88–111 (2009).

56. K. Okazaki, H. Takamuku, Y. Kawakubo, M. Hudson, J. Chen, Cranial morphometric analysis of early wet-rice farmers in the Yangtze River Delta of China. *Anthropol. Sci.*, 210325 (2021).

57. M. Shichirō, R. A. Miller, The Inariyama tumulus sword inscription. *J. Jpn. Stud.* **5**, 405–438 (1979).

58. C. B. Ramsey, Bayesian analysis of radiocarbon dates. *Radiocarbon* **51**, 337–360 (2009).

59. B. Llamas, G. Valverde, L. Fehren-Schmitz, L. S. Weyrich, A. Cooper, W. Haak, From the field to the laboratory: Controlling DNA contamination in human ancient DNA research in the high-throughput sequencing era. *Sci. Technol. Archaeol. Res.* **3**, 1–14 (2017).

60. S. Boessenkool, K. Hanghøj, H. M. Nistelberger, C. Der Sarkissian, A. T. Gondek, L. Orlando, J. H. Barrett, B. Star, Combining bleach and mild predigestion improves ancient DNA recovery from bones. *Mol. Ecol. Resour.* **17**, 742–751 (2017).

61. P. B. Damgaard, A. Margaryan, H. Schroeder, L. Orlando, E. Willerslev, M. E. Allentoft, Improving access to endogenous DNA in ancient bones and teeth. *Sci. Rep.* **5**, 11184 (2015).

62. M. Meyer, M. Kircher, Illumina sequencing library preparation for highly multiplexed target capture and sequencing. *Cold Spring Harb. Protoc.* **2010**, pdb.prot5448 (2010).

63. N. Rohland, E. Harney, S. Mallick, S. Nordenfelt, D. Reich, Partial uracil–DNA–glycosylase treatment for screening of ancient DNA. *Philos. Trans. R. Soc. Lond. B Biol. Sci.* **370**, 20130624 (2015).

64. M. Martin, Cutadapt removes adapter sequences from high-throughput sequencing reads. *EMBnet.J.* **17**, 10–12 (2011).

65. M. Schubert, S. Lindgreen, L. Orlando, AdapterRemoval v2: Rapid adapter trimming, identification, and read merging. *BMC. Res. Notes* **9**, 88 (2016).

66. H. Li, R. Durbin, Fast and accurate short read alignment with Burrows–Wheeler transform. *Bioinformatics* **25**, 1754–1760 (2009).

67. H. Li, B. Handsaker, A. Wysoker, T. Fennell, J. Ruan, N. Homer, G. Marth, G. Abecasis, R. Durbin; 1000 Genome Project Data Subgroup, The sequence alignment/map format and SAMtools. *Bioinformatics* **25**, 2078–2079 (2009).

68. H. Jónsson, A. Ginolhac, M. Schubert, P. L. F. Johnson, L. Orlando, mapDamage2.0: Fast approximate Bayesian estimates of ancient DNA damage parameters. *Bioinformatics* **29**, 1682–1684 (2013).

69. A. McKenna, M. Hanna, E. Banks, A. Sivachenko, K. Cibulskis, A. Kernytsky, K. Garimella, D. Altshuler, S. Gabriel, M. Daly, M. A. DePristo, The Genome Analysis Toolkit: A MapReduce framework for analyzing next-generation DNA sequencing data. *Genome Res.* **20**, 1297–1303 (2010).

70. S. Andrews, FastQC: A Quality Control Tool for High Throughput Sequence Data (2010); www.bioinformatics.babraham.ac.uk/projects/fastqc/.

71. D. Vianello, F. Sevini, G. Castellani, L. Lomartire, M. Capri, C. Franceschi, HAPLOFIND: A new method for high-throughput mtDNA haplogroup assignment. *Hum. Mutat.* **34**, 1189–1194 (2013).

72. P. Skoglund, J. Storå, A. Götherström, M. Jakobsson, Accurate sex identification of ancient human remains using DNA shotgun sequencing. *J. Archaeol. Sci.* **40**, 4477–4482 (2013).

73. J. M. Monroy Kuhn, M. Jakobsson, T. Günther, Estimating genetic kin relationships in prehistoric populations. *PLOS ONE* **13**, e0195491 (2018).

74. N. Patterson, A. L. Price, D. Reich, Population structure and eigenanalysis. *PLOS Genet.* **2**, e190 (2006).

75. D. H. Alexander, J. Novembre, K. Lange, Fast model-based estimation of ancestry in unrelated individuals. *Genome Res.* **19**, 1655–1664 (2009).

76. S. Purcell, B. Neale, K. Todd-Brown, L. Thomas, M. A. R. Ferreira, D. Bender, J. Maller, P. Sklar, P. I. W. de Bakker, M. J. Daly, P. C. Sham, PLINK: A tool set for whole-genome association and population-based linkage analyses. *Am. J. Hum. Genet.* **81**, 559–575 (2007).

77. N. Patterson, P. Moorjani, Y. Luo, S. Mallick, N. Rohland, Y. Zhan, T. Genschoreck, T. Webster, D. Reich, Ancient admixture in human history. *Genetics* **192**, 1065–1093 (2012).

78. J. V. Moreno-Mayar, B. A. Potter, L. Vinner, M. Steinrücken, S. Rasmussen, J. Terhorst, J. A. Kamm, A. Albrechtsen, A.-S. Malaspinas, M. Sikora, J. D. Reuther, J. D. Irish, R. S. Malhi, L. Orlando, Y. S. Song, R. Nielsen, D. J. Meltzer, E. Willerslev, Terminal Pleistocene Alaskan genome reveals first founding population of Native Americans. *Nature* **553**, 203–207 (2018).

79. P. Danecek, A. Auton, G. Abecasis, C. A. Albers, E. Banks, M. A. DePristo, R. E. Handsaker, G. Lunter, G. T. Marth, S. T. Sherry, G. McVean, R. Durbin; 1000 Genomes Project Analysis Group, The variant call format and VCFtools. *Bioinformatics* **27**, 2156–2158 (2011).

80. R. N. Gutenkunst, R. D. Hernandez, S. H. Williamson, C. D. Bustamante, Inferring the joint demographic history of multiple populations from multidimensional SNP frequency data. *PLOS Genet.* **5**, e1000695 (2009).

81. R. R. Hudson, Generating samples under a Wright–Fisher neutral model of genetic variation. *Bioinformatics* **18**, 337–338 (2002).

82. N. Osada, S. Nakagome, S. Mano, Y. Kameoka, I. Takahashi, K. Terao, Finding the factors of reduced genetic diversity on X chromosomes of *Macaca fascicularis*: Male-driven evolution, demography, and natural selection. *Genetics* **195**, 1027–1035 (2013).

83. R. E. Kass, A. E. Raftery, Bayes factors. *J. Am. Stat. Assoc.* **90**, 773–795 (1995).

84. W. Haak, I. Lazaridis, N. Patterson, N. Rohland, S. Mallick, B. Llamas, G. Brandt, S. Nordenfelt, E. Harney, K. Stewardson, Q. Fu, A. Mittnik, E. Bánffy, C. Economou, M. Francken, S. Friederich, R. G. Pena, F. Hallgren, V. Khartanovich, A. Khokhlov, M. Kunst, P. Kuznetsov, H. Meller, O. Mochalov, V. Moiseyev, N. Nicklisch, S. L. Pichler, R. Risch, M. A. Rojo Guerra, C. Roth, A. Szécsényi-Nagy, J. Wahl, M. Meyer, J. Krause, D. Brown, D. Anthony, A. Cooper, K. W. Alt, D. Reich, Massive migration from the steppe was a source for Indo-European languages in Europe. *Nature* **522**, 207–211 (2015).

85. M. A. Yang, X. Gao, C. Theunert, H. Tong, A. Aximu-Petri, B. Nickel, M. Slatkin, M. Meyer, S. Pääbo, J. Kelso, Q. Fu, 40,000-year-old individual from Asia provides insight into early population structure in Eurasia. *Curr. Biol.* **27**, 3202–3208.e9 (2017).

86. M. Raghavan, P. Skoglund, K. E. Graf, M. Metspalu, A. Albrechtsen, I. Moltke, S. Rasmussen, T. W. Stafford Jr., L. Orlando, E. Metspalu, M. Karmin, K. Tambets, S. Rootsi, R. Mägi, P. F. Campos, E. Balanovska, O. Balanovsky, E. Khusnutdinova, S. Litvinov, L. P. Osipova, S. A. Fedorova, M. I. Voevoda, M. DeGiorgio, T. Sicheritz-Ponten, S. Brunak, S. Demeshchenko, T. Kivisild, R. Villems,

R. Nielsen, M. Jakobsson, E. Willerslev, Upper Palaeolithic Siberian genome reveals dual ancestry of Native Americans. *Nature* **505**, 87–91 (2014).

87. J. Habu, C. Fawcett, Jomon archaeology and the representation of Japanese origins. *Antiquity* **73**, 587–593 (1999).

88. Y. Nakazawa, On the Pleistocene population history in the Japanese archipelago. *Curr. Anthropol.* **58**, S539–S552 (2017).

89. T. Gakuhari, H. Komiya, J. Sawada, T. Anezaki, T. Sato, K. Kobayashi, S. Itoh, K. Kobayashi, H. Matsuzaki, K. Yoshida, M. Yoneda, Radiocarbon dating of one human and two dog burials from the Kamikuroiwa rock shelter site, Ehime Prefecture. *Anthropol. Sci.* **123**, 87–94 (2015).

90. A. Scally, R. Durbin, Revising the human mutation rate: Implications for understanding human evolution. *Nat. Rev. Genet.* **13**, 745–753 (2012).

91. C. Jeong, A. T. Ozga, D. B. Witonsky, H. Malmström, H. Edlund, C. A. Hofman, R. W. Hagan, M. Jakobsson, C. M. Lewis, M. S. Aldenderfer, A. Di Rienzo, C. Warinner, Long-term genetic stability and a high-altitude East Asian origin for the peoples of the high valleys of the Himalayan arc. *Proc. Natl. Acad. Sci. U.S.A.* **113**, 7485–7490 (2016).

92. D. Massilani, L. Skov, M. Hajdinjak, B. Gunchinsuren, D. Tseveendorj, S. Yi, J. Lee, S. Nagel, B. Nickel, T. Devièse, T. Higham, M. Meyer, J. Kelso, B. M. Peter, S. Pääbo, Denisovan ancestry and population history of early East Asians. *Science* **370**, 579–583 (2020).

93. R. J. Hijmans, E. Williams, C. Vennes, Geosphere: Spherical trigonometry, R package version 1 (2016).
